# Addendum: Multipeptide vaccines for melanoma in the adjuvant setting: long-term survival outcomes and post-hoc analysis of a randomized phase II trial

**DOI:** 10.1038/s41467-025-63690-x

**Published:** 2025-09-05

**Authors:** Emily K. Ninmer, Hong Zhu, Kimberly A. Chianese-Bullock, Margaret von Mehren, Naomi B. Haas, Merrick I. Ross, Lynn T. Dengel, Craig L. Slingluff

**Affiliations:** 1 Department of Surgery/Division of Surgical Oncology and the Human Immune Therapy Center, Cancer Center, University of Virginia, Charlottesville, VA USA; 2 Department of Public Health Sciences, University of Virginia, Charlottesville, VA USA; 3 University of Virginia, School of Medicine, Cancer Center, Charlottesville, VA USA; 4 Fox Chase Cancer Center, Philadelphia, PA USA; 5 University of Pennsylvania, Philadelphia, PA USA; 6 Department of Surgical Oncology, MD Anderson Cancer Center, Houston, TX USA

Addendum to: *Nature Communications* 10.1038/s41467-024-46877-6, published online 22 March 2024

In the original version of this manuscript, we reported a post-hoc analysis of long-term clinical outcomes of a randomized multicenter clinical trial comparing two experimental melanoma vaccines, with or without low dose cyclophosphamide (Cy). The data supported prolonged long-term survival of patients treated with a vaccine comprised of 12 class I MHC-restricted peptides designed to induce CD8^+^ T cell responses (12MP) plus 6 class II MHC-restricted peptides designed to induce CD4^+^ T cell responses to melanoma antigens (6MHP), compared to vaccination with 12MP plus a tetanus toxoid peptide for activation of CD4^+^ T cells that were not melanoma-cognate.

Our team is pursuing additional studies of tumor samples collected from some of the patients on this trial, and in planning those experiments, we identified errors in our originally published source data file due to idiosyncrasies in how some follow-up dates were coded in our clinical trials database. Thus, we performed a detailed search of the clinical trials data to identify any other complexities that may not have been captured by our original data extraction process. From this audit, we identified errors in our published source data due to inadequate capture of data from the survival follow-up forms and inconsistencies in how the survival and recurrence data were reported in the database. These errors included 17 participants for whom overall survival (OS) was underestimated, 4 and 3 participants for whom recurrence-free survival (RFS) was underestimated and overestimated, respectively, and 7 participants for whom a recurrence was not originally captured. All identified errors have been corrected. A corrected source data file is now available alongside the article.

Using the corrected data, we repeated all analyses of our original manuscript and report in this Addendum the results of the corrected analyses, with revised figures and tables for those affected in the main text. A revised Supplementary Figs. and tables file is available alongside the article. The original conclusions are supported by the analysis of the corrected dataset.

From the corrected survival data, the median follow-up interval was 8.7 years for all participants and 12.5 years for participants alive at the date of last known follow-up, as of data lock in April 2023. Of the participants with corrected OS data, about half were on arm D (12MP + 6MHP+Cy), resulting in a disproportionate underestimation of OS for that group in the original report. Thus, the corrected dataset more strongly supports some of the conclusions regarding the OS benefit after vaccination with melanoma cognate help than in our original report. The corrected Kaplan-Meier survival curves for OS in the intention-to-treat population (ITT) are shown in Addendum Figure 1A–C. Consistent with our original report, there was a favorable trend to improved OS with melanoma cognate help (HR 0.64, 95% CI: 0.40–1.04, *P* = 0.07, Addendum Figure 1A; original report: HR 0.65, 95% CI: 0.40–1.05, *P* = 0.08), with delayed separation of the curves starting at 2.5 years and progressive widening of the curves such that the OS estimate for the 12MP + 6MHP regimen exceeded the upper bound of the 95% CI of the OS estimate for the 12MP+tet regimen at 8 years (Addendum Figure 1B). Also consistent with our original report, landmark analysis at 2.5 years for OS by vaccine regimen supports durable prolongation of OS after vaccination with melanoma cognate help (HR 0.51, 95% CI: 0.27–0.95, *P* = 0.04, Addendum Figure 1C; original report: HR 0.52, 95% CI: 0.27–0.97, *P* = 0.04). Similar trends as reported in our original manuscript were found on Kaplan-Meier analysis of OS by vaccine regimen and sex (Addendum Figure 2A) and vaccine regimen and disease stage (Addendum Figure 3A, C). Cox regression models for OS using the corrected dataset also support the conclusions of our original manuscript, with age, sex, and study arm (A vs. D) as significant predictors of OS on multivariable analysis; disease stage remains in the final model as originally reported but is no longer statistically significant (Addendum Table 1). Consistent with our original report, age and vaccine regimen or study arm (A vs. D) were significant predictors of OS on Cox regression models for the 2.5-year landmark; advanced disease status and Eastern Cooperative Study Group performance status (ECOG PS) remain in the final models as originally reported (Addendum Table 2).

Of the 7 additional recurrences captured in the corrected data, all occurred within 4.6–8.1 years after enrollment, including 2 cases of participant-reported new primaries (arms B and D) and 2 cases of deaths reported due to disease without prior documentation of recurrence (arms A and C). For the recurrences that were only identified by death reported due to disease, the date of death was used as the date of recurrence to allow for capture of the recurrence, though we acknowledge that the recurrences may have been detected earlier and may represent an overestimation of RFS in those cases. From the corrected RFS data, participants vaccinated with 12MP+tet and 12MP + 6MHP had median RFS of 2.0 years and 6.0 years, respectively. Consistent with the original report, there was a weak trend to improved RFS with melanoma cognate help (HR 0.75, 95% CI: 0.50–1.12, *P* = 0.16, Addendum Figure 1D; original report: HR 0.77, 95% CI: 0.51–1.18, *P* > 0.22).

Six of the additional 7 recurrences captured in the corrected data were in males. The corrected dataset still supports the original conclusions regarding RFS, including the favorable benefit from vaccination with melanoma cognate help confined to males (Addendum Figure 2B) with significantly improved RFS in males vaccinated with 12MP + 6MHP compared to 12MP+tet on landmark analysis at end of treatment (1 year) (HR 0.37, 95% CI: 0.17–0.81, *P* = 0.01, Addendum Figure 2C; original report: HR 0.35, 95% CI: 0.14–0.86, *P* = 0.02). Consistent with our original report, significant predictors of RFS on multivariable analysis included young age (≤40 years) and male sex (corrected Supplementary Tables 5 and 6); however, for the 1-year landmark, no covariates were significant predictors of RFS, though there remained a favorable trend for males (corrected Supplementary Table 7).

In summary, we identified errors in our original source data file and now provide a corrected source data file, as well as revised figures and tables reporting outcomes with the original article. Tables reporting patient demographics and clinical features are not changed. The corrected data support the conclusions previously reported in our original manuscript, with durable prolongation of OS with the addition of cognate helper peptides to vaccine for which a disproportionate benefit was found among males and in the setting of Cy pretreatment.

## Supplementary information

Addendum Figures and Tables

