# Supplementary material for: Multipeptide vaccines for melanoma in the adjuvant setting: long-term survival outcomes and post-hoc analysis of a randomized phase II trial
Source: Nat Commun. 2024 Mar 22;15:2570. doi: 10.1038/s41467-024-46877-6 (PMC10959948; doi:10.1038/s41467-024-46877-6)
Supplement: Supplementary file 1 — Supplementary Information [file 41467_2024_46877_MOESM1_ESM.pdf]

Supplemental Figures

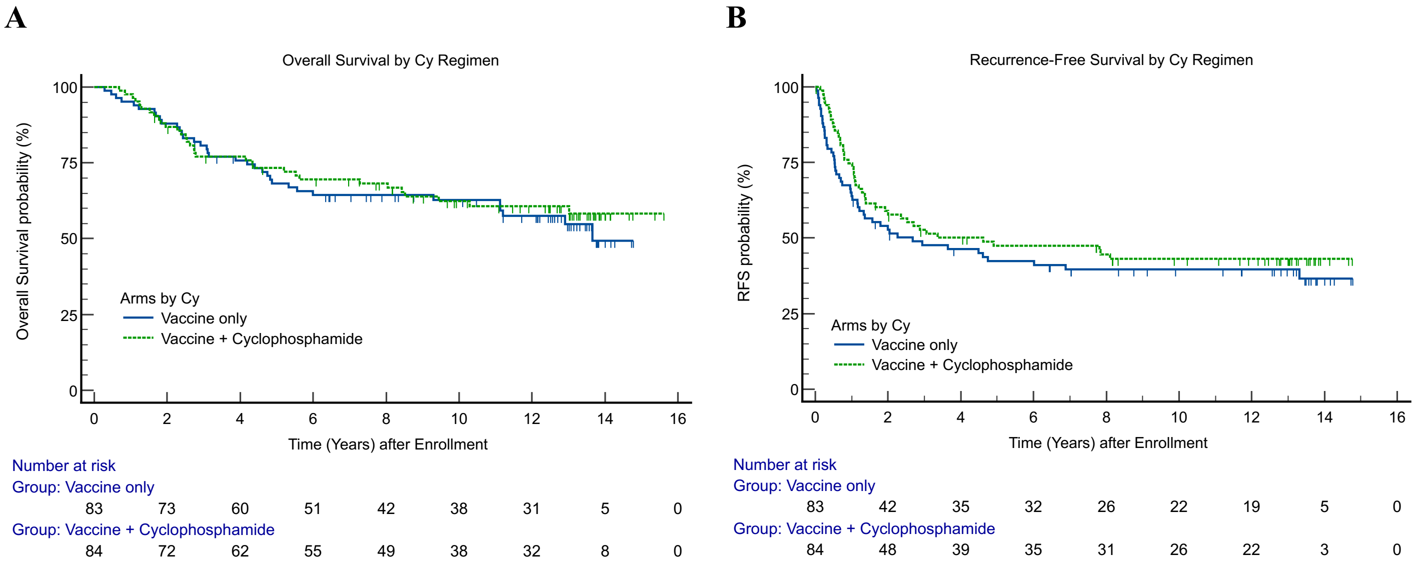

Supplemental Figure 1. Overall survival and recurrence free survival by cyclophosphamide regimen: Outcomes by Cy regimen (A+C vs B+D) (A) OS by Cy regimen (HR 0.89, 95% CI 0.55-1.43; p=0.63), (B) RFS by Cy regimen (HR 0.82, 95% CI 0.55-1.22; p=0.32). Source data are provided as a Source Data file.

A

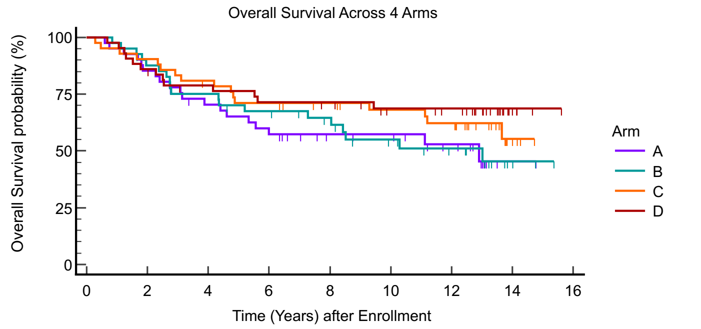

Number at risk

	0	2	4	6	8	10	12	14	16
Group: A	41	35	27	22	16	15	10	1	0
Group: B	41	35	30	26	21	15	11	3	0
Group: C	42	38	33	29	26	23	21	4	0
Group: D	43	37	32	29	28	23	21	5	0

Hazard ratios with 95% Confidence Interval

Factor	Arm B	Arm C	Arm D
Arm A	0.94<br>0.47 to 1.91	0.69<br>0.35 to 1.38	0.56<br>0.28 to 1.11
Arm B	-	0.74<br>0.37 to 1.44	0.60<br>0.30 to 1.17
Arm C	-	-	0.81<br>0.42 to 1.55

B

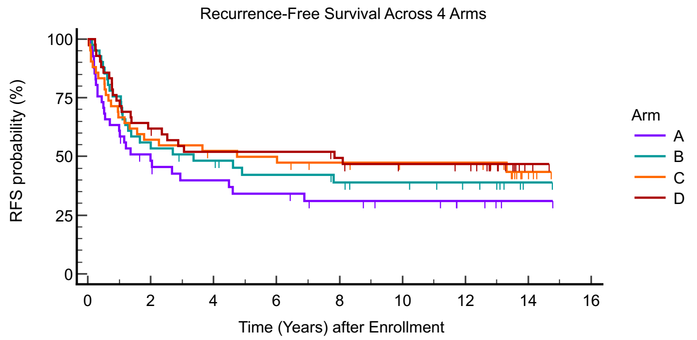

Number at risk

	0	2	4	6	8	10	12	14	16
Group: A	41	18	14	12	9	7	4	1	0
Group: B	41	22	18	14	12	10	7	1	0
Group: C	42	24	21	20	17	15	15	4	0
Group: D	43	26	21	21	19	16	15	2	0

Hazard ratios with 95% Confidence Interval

Factor	Arm B	Arm C	Arm D
Arm A	0.74<br>0.41 to 1.33	0.68<br>0.38 to 1.22	0.61<br>0.34 to 1.09
Arm B	-	0.92<br>0.53 to 1.61	0.83<br>0.48 to 1.44
Arm C	-	-	0.90<br>0.52 to 1.55

C

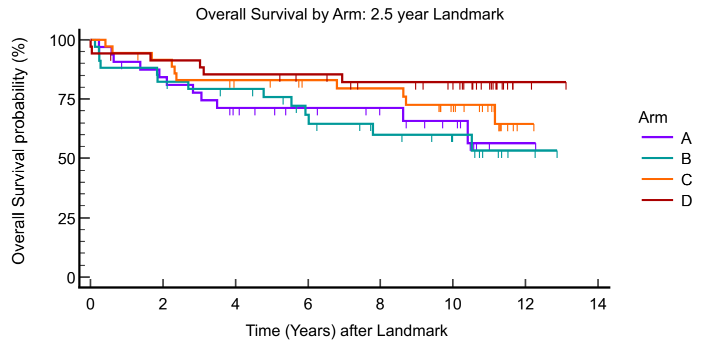

Number at risk

	0	2	4	6	8	10	12	14
Group: A	33	26	20	16	13	9	1	0
Group: B	34	28	25	18	13	9	2	0
Group: C	36	32	27	24	23	17	1	0
Group: D	35	31	29	27	23	19	2	0

Hazard ratios with 95% Confidence Interval

Factor	Arm B	Arm C	Arm D
Arm A	1.08<br>0.42 to 2.76	0.67<br>0.27 to 1.65	0.40<br>0.16 to 0.98
Arm B	-	0.62<br>0.26 to 1.49	0.37<br>0.16 to 0.89
Arm C	-	-	0.60<br>0.26 to 1.36

Supplemental Figure 2. Overall survival and recurrence-free survival across all 4 arms: (A) OS for all eligible participants (n=167) by study arm (p=0.32) (B) RFS for all eligible participants (n=167) by study arm (p=0.32), (C) Landmark analysis for OS at 2.5 years for eligible participants (n=138) by study arm, (p=0.16). Hazard ratios and 95% confidence intervals are shown for each pair of Kaplan-Meier curves. Source data are provided as a Source Data file.

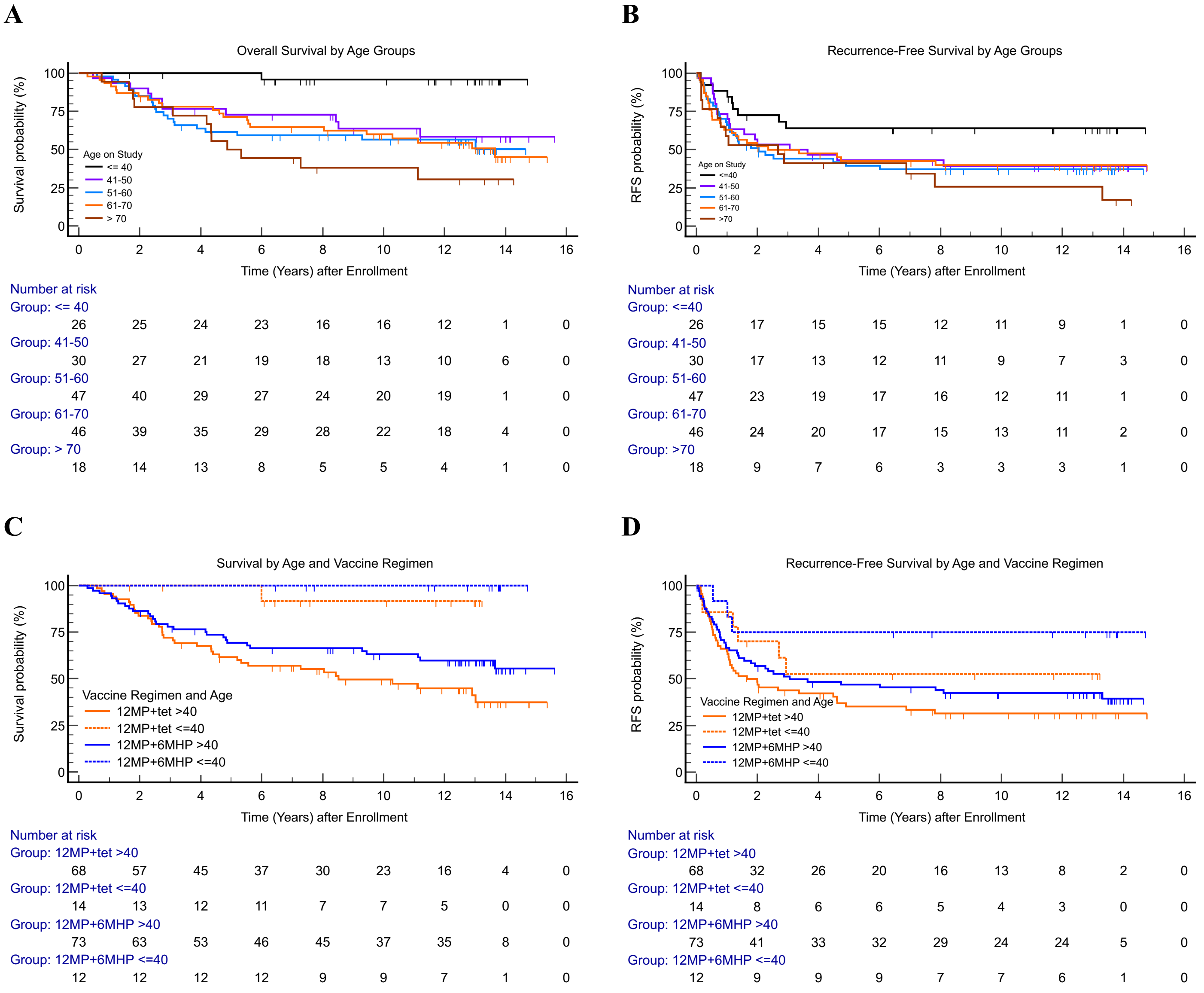

Supplemental Figure 3. Overall survival and recurrence-free survival by age groups: (A) OS for all eligible participants (n=167) by age groups ($p < 0.002$) (B) RFS for all eligible participants (n=167) by age groups ($p=0.16$ overall) (C) OS for all eligible participants (n=167) by age group categories (<40 years and >= 40 years) and by vaccine regimen ($p < 0.001$), (D) RFS for all eligible participants (n=167) by age group categories (<40 years and >= 40 years) and by vaccine regimen ($p=0.06$). Source data are provided as a Source Data file.

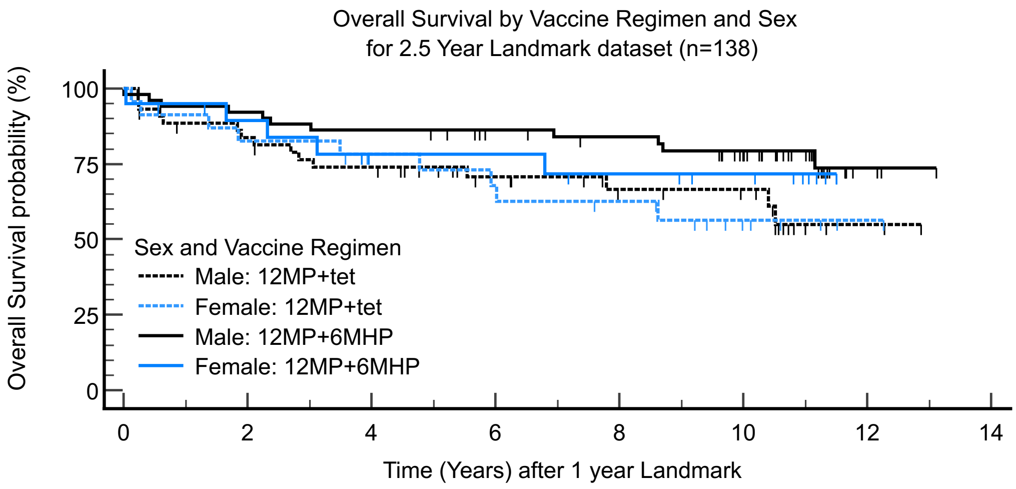

Number at risk

Group	0	2	4	6	8	10	12	14
Group: Male: 12MP+tet	44	35	30	21	15	13	2	0
Group: Female: 12MP+tet	23	19	15	13	11	5	1	0
Group: Male: 12MP+6MHP	51	47	44	39	36	28	3	0
Group: Female: 12MP+6MHP	20	16	12	12	10	8	0	0

Hazard ratios with 95% Confidence Interval

Factor	Female: 12MP+tet	Male: 12MP+6MHP	Female: 12MP+6MHP
Male: 12MP+tet	1.062<br>0.400 to 2.823	0.482<br>0.226 to 1.027	0.662<br>0.240 to 1.827
Female: 12MP+tet	-	0.454<br>0.182 to 1.133	0.623<br>0.200 to 1.945
Male: 12MP+6MHP	-	-	1.374<br>0.529 to 3.568

Supplemental Figure 4. Overall survival by vaccine regimen and sex in 2.5 year landmark analysis: OS for vaccine regimen and sex (p=0.19 overall). Source data are provided as a Source Data file.

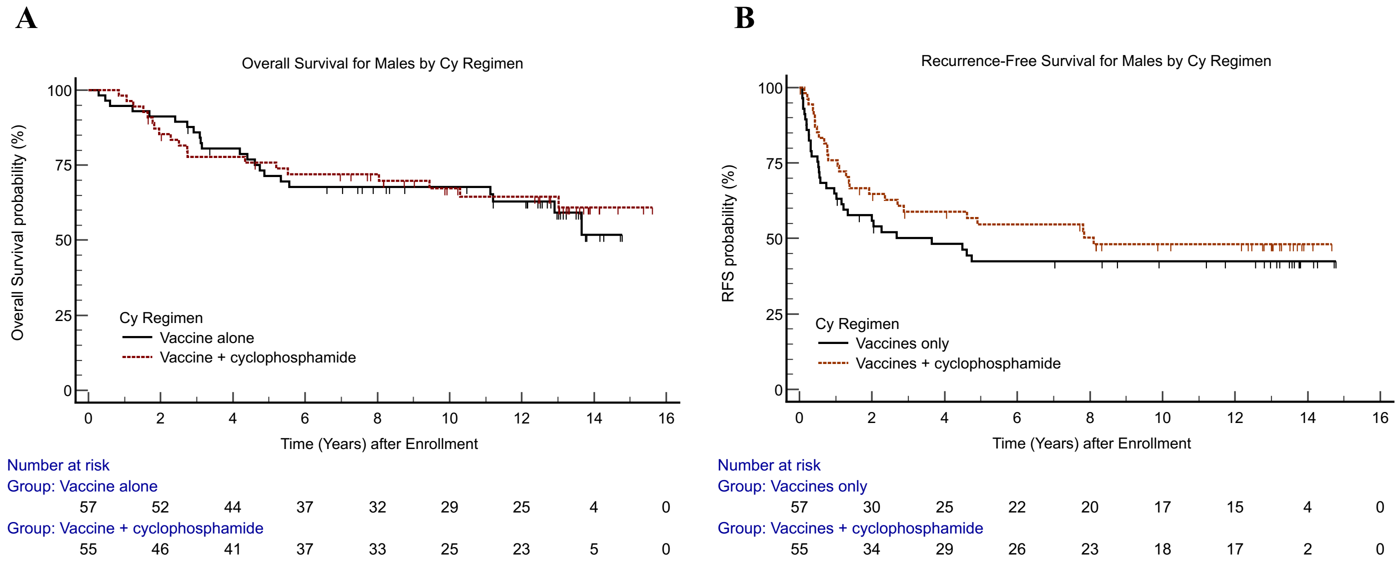

Supplemental Figure 5. Overall survival and recurrence-free survival for male participants by cyclophosphamide regimen: Outcomes for male participants (n = 112) by Cy regimen: (A) OS by Cy regimen for male participants (HR 0.92, 95% CI: 0.50-1.70; p = 0.79, (B) RFS by Cy regimen for male participants (HR 0.76, 95% CI: 0.45-1.27; p=0.29). Source data are provided as a Source Data file.

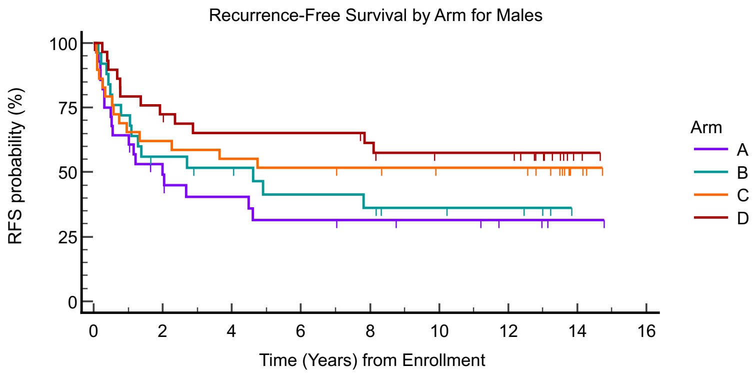

Number at risk

	0	2	4	6	8	10	12	14	16
Group: A	28	12	9	7	6	5	3	1	0
Group: B	25	13	11	8	7	5	4	0	0
Group: C	29	18	16	15	14	12	12	3	0
Group: D	30	21	18	18	16	13	13	2	0

Hazard ratios with 95% Confidence Interval

Factor	B	C	D
A	0.79<br>0.36 to 1.73	0.61<br>0.29 to 1.28	0.45<br>0.22 to 0.93
B	-	0.77<br>0.37 to 1.61	0.57<br>0.28 to 1.16
C	-	-	0.74<br>0.38 to 1.46

Supplemental Figure 6. Recurrence-free survival across all 4 arms for males: Outcomes for male participants (n=112) by study arm (p=0.15). Hazard ratios and 95% confidence intervals are shown for each pair of Kaplan-Meier curves. Source data are provided as a Source Data file.

Supplementary Table 1. Source proteins for peptides in 12MP and 6MHP vaccines

**Source protein**	**12MP (Class I MHC) NSC # 728925**	**6MHP (promiscuous for Class II MHC) NSC # 728926**
**gp100**	IMDQVPFSV (A2)<br>YLEPGPVTA (A2)<br>ALLAVGATK (A3)<br>LIYRRRLMK (A3)	WNRQLYPEWTEAQRLD
**Tyrosinase**	DAEKSDICTDEY (A1)<br>SSDYVIPIGTY (A1)<br>YMDGTMSQV (A2)	AQNILLSNAPLGPQFP<br>FLLHHAFVDSIFEQWLQRHRP
**MAGE-A1**	EADPTGHSY (A1)<br>SLFRAVITK (A3)	---
**MAGE-A1,A2,A3,A6**	---	LLKYRAREPVTKAE
**MAGE-A3**	EVDPIGHLY (A1)	TSYVKVLHHMVKISG
**MAGE-A10**	GLYDGMEHL (A2)	---
**NY-ESO-1**	ASGPGGGAPR (A3, A31, A33)	---
**MelanA/MART-1**	---	RNGYRALMDKSLHVGTQCALTRR

Tetanus toxoid derived helper peptide (AQYIKANSKFIGITEL) binds to multiple class II alleles. Peptide sequences are provided with single-letter abbreviations for each amino acid.

Supplementary Table 2. Participant features for subset analyses, by sex and vaccine regimen

	Female			Male		
	Arms A+B (12MP + tet) (n = 29)	Arms C+D (12MP + 6MHP) (n = 26)	p value*	Arms A+B (12MP + tet) (n = 53)	Arms C+D (12MP + 6MHP) (n = 59)	p value*
**Male, n (%)**	0 (0%)	0 (0%)	--	53 (100%)	59 (100%)	--
**Institution, n (%)**						
**FCCC**	3 (10%)	8 (31%)	0.08	12 (23%)	5 (8%)	0.08
**MDACC**	7 (24%)	8 (31%)		18 (34%)	19 (32%)	
**UVA**	19 (66%)	10 (39%)		23 (43%)	35 (59%)	
**Diagnosis at enrollment, n (%)**						
**Initial diagnosis**	14 (48%)	13 (50%)	0.90	23 (43%)	35 (59%)	0.16
**Recurrence**	15 (52%)	13 (50%)		30 (57%)	24 (41%)	
**AJCC v8 stage at enrollment, n (%)**						
**IIB-C**	4 (14%)	3 (12%)	0.88	5 (9%)	12 (20%)	0.15
**IIIA**	3 (10%)	4 (15%)		7 (13%)	6 (10%)	
**IIIB-D**	16 (55%)	12 (46%)		28 (53%)	32 (54%)	
**IV (M1a/b)**	3 (10%)	5 (19%)		9 (17%)	9 (15%)	
**IV (M1c/d)**	3 (10%)	2 (8%)		4 (8%)	0 (0%)	
**ECOG PS Score 0, n (%)**	27 (93%)	23 (88%)	0.66	46 (87%)	55 (93%)	0.34
**LDH at enrollment**						
**N evaluable**	28	26		53	59	
**N < ULN (%)**	28 (100%)	26 (100%)	--	49 (92%)	57 (97%)	0.42
**N < 1.5x ULN (%)**	28 (100%)	26 (100%)	--	53 (100%)	59 (100%)	--
**Median age, years (range)**	54.9 (28.8 – 80.6)	51.5 (21.4 – 77.3)	0.62	60.9 (28.9 – 81.7)	58.4 (25.8 – 75.5)	0.52
**Class I MHC allele, n (%)**						
**HLA-A1**	10 (34%)	8 (31%)	0.77	15 (28%)	19 (32%)	0.66
**HLA-A2**	16 (55%)	15 (58%)	0.85	28 (53%)	26 (44%)	0.36
**HLA-A3/31**	10 (34%)	10 (38%)	0.76	26 (49%)	31 (53%)	0.71

Abbreviations: 12MP, 12 class I MHC-restricted melanoma peptides; tet, tetanus toxoid helper peptide; 6MHP, mixture of six melanoma-specific helper peptides; FCCC, Fox Chase Cancer Center; MDACC, MD Anderson Cancer Center; UVA, University of Virginia; AJCC, American Joint Committee on Cancer; ECOG PS, Eastern Cooperative Oncology Group Performance Status; LDH, lactate dehydrogenase; MHC, major histocompatibility complex; HLA, human leukocyte antigen

*Chi-square or Fisher's exact test (two-sided) for categorical variables; Mann-Whitney test (two-sided) for age

Supplementary Table 3. Participant features for subset analyses, by AJCC stage and vaccine regimen

	Stage IIB-III			Stage IV		
	**Arms A+B (12MP + tet) (n = 63)**	**Arms C+D (12MP + 6MHP) (n = 69)**	**p value***	**Arms A+B (12MP + tet) (n = 19)**	**Arms C+D (12MP + 6MHP) (n = 16)**	**p value***
**Male, n (%)**	40 (63%)	50 (72%)	0.27	13 (68%)	9 (56%)	0.50
**Institution, n (%)**						
**FCCC**	12 (19%)	12 (17%)		3 (16%)	1 (6%)	0.80
**MDACC**	16 (25%)	18 (26%)	0.97	9 (47%)	9 (56%)	
**UVA**	35 (56%)	39 (57%)		7 (37%)	6 (38%)	
**Diagnosis at enrollment, n (%)**						
**Initial diagnosis**	33 (52%)	46 (67%)	0.10	4 (21%)	2 (13%)	0.67
**Recurrence**	30 (48%)	23 (33%)		15 (79%)	14 (88%)	
**AJCC v8 stage at enrollment, n (%)**						
**IIB-C**	9 (11%)	15 (18%)		--	--	
**IIIA**	10 (12%)	10 (12%)	0.54	--	--	0.14
**IIIB-D**	44 (54%)	44 (53%)		--	--	
**IV (M1a/b)**	--	--		12 (15%)	14 (16%)	
**IV (M1c/d)**	--	--		7 (9%)	2 (2%)	
**ECOG PS Score 0, n (%)**	55 (87%)	62 (90%)	0.65	18 (95%)	16 (100%)	1.00
**LDH at enrollment**						
**N evaluable**	62	69		19	16	
**N < ULN (%)**	58 (94%)	67 (97%)	0.42	19 (100%)	16 (100%)	--
**N < 1.5x ULN (%)**	62 (100%)	69 (100%)	--	19 (100%)	16 (100%)	--
**Median age, years (range)**	59.5 (28.8 – 80.6)	55.5 (21.4 – 75.6)	0.31	60.3 (35.6 – 81.7)	57.4 (42.3 – 77.3)	0.95
**Class I MHC allele, n (%)**						
**HLA-A1**	18 (29%)	22 (32%)	0.68	7 (37%)	5 (31%)	0.73
**HLA-A2**	36 (57%)	34 (49%)	0.37	8 (42%)	7 (44%)	0.92
**HLA-A3/31**	26 (41%)	31 (45%)	0.67	10 (53%)	10 (63%)	0.56

Abbreviations: 12MP, 12 class I MHC-restricted melanoma peptides; tet, tetanus toxoid helper peptide; 6MHP, mixture of six melanoma-specific helper peptides; FCCC, Fox Chase Cancer Center; MDACC, MD Anderson Cancer Center; UVA, University of Virginia; AJCC, American Joint Committee on Cancer; ECOG PS, Eastern Cooperative Oncology Group Performance Status; LDH, lactate dehydrogenase; ULN, upper limit of normal; MHC, major histocompatibility complex; HLA, human leukocyte antigen
*Chi-square or Fisher's exact test (two-sided) for categorical variables; Mann-Whitney test (two-sided) for age.

Supplementary Table 4. Cox regression model for overall survival for intention-to-treat population (n = 167) based on pairs of study arms (revised with corrected data)

Multivariable model for OS all eligible patients (n = 167; p < 0.0001, Chi-square 37.2: Including vaccine regimen (AB vs CD) and Cy arms (AC vs BD), but not Study Arm				
**Covariate**	***Detail***	***p value***	***HR***	***95% CI***
Age	≤ 40 vs > 40 years	**0.002***	0.046	0.006 to 0.336
Sex	Female vs male	**0.025***	1.811	1.078 to 3.043
Vaccine Regimen: 6MHP (yes/no)	12MP+tet vs. 12MP+6MHP	*0.071#*	1.585	0.962 to 2.611
Cyclophosphamide Arms: Cy (yes/no)	No Cy vs. +Cy	*0.082#*	1.554	0.946 to 2.554
Advanced disease status	Recurrence vs initial diagnosis	0.236	0.732	0.437 to 1.227
LDH level	High vs normal or not done	0.231	2.101	0.624 to 7.081
AJCC stage, v8	Stage IV vs II-III	0.208	0.638	0.317 to 1.283
ECOG PS score	1 vs 0	0.369	1.379	0.684 to 2.782
Refined model (p < 0.0001, Chi-squared 29.4): OS all eligible patients (n = 167)				
**Covariate**	***Detail***	***p value***	***HR***	***95% CI***
Age	≤ 40 vs > 40 years	**0.003***	0.049	0.007 to 0.352
Sex	Female vs male	**0.035***	1.727	1.039 to 2.872
AJCC stage, v8	Stage IV vs II-III	*0.0507#*	0.524	0.274 to 1.002
Vaccine Regimen: 6MHP (yes/no)	12MP+tet vs. 12MP+6MHP	*0.081#*	1.553	0.948 to 2.545
Cyclophosphamide Arms: Cy (yes/no)	No Cy vs. +Cy	*0.090#*	1.530	0.936 to 2.501

Abbreviations: 6MHP, 6 melanoma helper peptides; 12MP, 12 melanoma peptides restricted by Class I MHC; tet, tetanus helper peptide; HR, hazard ratio; CI, confidence interval; LDH, lactate dehydrogenase; AJCC v8, American Joint Committee on Cancer version 8; ECOG PS, Eastern Cooperative Oncology Group Performance Status.

*Significant, $p < 0.05$ (bolded); # $0.05 < p < 0.10$ (italics); Adjustments were not made for multiple comparisons

Changes in significant variables from original published data: In refined model, AJCC stage is no longer a significant covariate.

Supplementary Table 5. Cox Regression Model for RFS for intention-to-treat population (n = 167) (revised with corrected data)

Multivariable model for RFS for all eligible patients (n = 167; p = 0.013, Chi-square 14.51):				
Covariate	***Detail***	***p value***	***HR***	***95% CI of HR***
**Age**	≤40 vs. >40 years	**0.006***	0.373	0.185 to 0.756
**Sex**	Female vs Male	**0.043***	1.541	1.014 to 2.342
**Study Arm**	A (12MP+Tet) vs D (12MP+6MHP+Cy)	**0.021***	1.702	1.082 to 2.677

Abbreviations: HR, hazard ratio; CI, confidence interval. 12MP, 12 class I MHC-restricted melanoma peptides; tet, tetanus toxoid helper peptide; 6MHP, mixture of six melanoma-specific helper peptides;
* Significant, $p < 0.05$ (bolded); Adjustments were not made for multiple comparisons.

Supplementary Table 6. Cox regression model for RFS for intention-to-treat population (n = 167) based on pairs of study arms (revised with corrected data)

Multivariable model for RFS all eligible patients (n = 167; p = 0.0433, Chi-square 15.9): Including vaccine regimen (AB vs CD) and Cy arms (AC vs BD), but not Study Arm				
**Covariate**	***Detail***	***p value***	***HR***	***95% CI***
Age	≤ 40 vs > 40 years	**0.016***	0.414	0.202 to 0.845
Sex	Female vs male	*0.079#*	1.467	0.957 to 2.250
Vaccine Regimen: 6MHP (yes/no)	12MP+tet vs. 12MP+6MHP	0.234	1.281	0.852 to 1.928
Cyclophosphamide Arms: Cy (yes/no)	No Cy vs. +Cy	0.143	1.363	0.901 to 2.060
Advanced disease status	Recurrence vs initial diagnosis	0.251	1.290	0.835 to 1.993
LDH level	High vs normal or not done	0.544	1.388	0.481 to 4.000
AJCC stage, v8	Stage IV vs II-III	0.891	0.965	0.579 to 1.608
ECOG PS score	1 vs 0	0.507	1.251	0.646 to 2.423
Refined model (p = 0.0086, Chi-squared 11.669): OS all eligible patients (n = 167)				
**Covariate**	***Detail***	***p value***	***HR***	***95% CI***
Age	≤ 40 vs > 40 years	**0.009***	0.393	0.195 to 0.791
Sex	Female vs male	**0.048***	1.525	1.005 to 2.315
Cyclophosphamide Arms: Cy (yes/no)	No Cy vs. +Cy	0.139	1.357	0.905 to 2.034

Abbreviations: 6MHP, 6 melanoma helper peptides; 12MP, 12 melanoma peptides restricted by Class I MHC; tet, tetanus helper peptide; HR, hazard ratio; CI, confidence interval; LDH, lactate dehydrogenase; AJCC v8, American Joint Committee on Cancer version 8; ECOG PS, Eastern Cooperative Oncology Group Performance Status

*Significant, $p < 0.05$ (bolded); # $0.05 < p < 0.10$ (italics)

Changes in significant variables from original published data: In the original report, no covariates were significant in the refined model, though age (p=0.050), sex (p=0.075), and advanced disease status (0.092) trended to significance. In the revised refined model, age and sex are significantly associated with RFS, and Cy arms show weak trend toward significance (p=0.139).

Supplemental Table 7. Cox regression model for RFS for 1 year landmark population (n = 115) (revised with corrected data)

Initial multivariable model for RFS for all eligible patients after 1 year landmark (n = 115; p = 0.276, Chi-square 9.85)				
**Covariate**	***Detail***	***p value***	***HR***	***95% CI***
Sex	Female vs male	*0.064*#	1.817	0.966 to 3.418
Age	≤ 40 vs > 40 years	0.276	0.598	0.237 to 1.508
ECOG PS score	1 vs 0	0.165	1.937	0.763 to 4.920
LDH level	High vs normal or not done	0.694	1.369	0.286 to 6.565
AJCC stage, v8	Stage IV vs II-III	0.676	1.177	0.549 to 2.521
Advanced disease status	Initial diagnosis vs. recurrence	0.934	1.028	0.536 to 1.969
Vaccine Regimen: 6MHP (yes/no)	12MP+tet vs. 12MP+6MHP	0.233	1.452	0.786 to 2.681
Cyclophosphamide Arms: Cy (yes/no)	No Cy vs. +Cy	0.895	0.959	0.515 to 1.787
Refined multivariable model for RFS for all eligible patients after 1 year landmark (n = 115; p = 0.0498, Chi-square 6.00)				
**Covariate**	***Detail***	***p value***	***HR***	***95% CI***
Sex	Female vs male	*0.062*#	1.941	1.064 to 3.541
ECOG PS	1 vs 0	*0.084*#	2.143	0.904 to 5.081

Abbreviations: HR, hazard ratio; CI, confidence interval; LDH, lactate dehydrogenase; AJCC v8, American Joint Committee on Cancer version 8; ECOG PS, Eastern Cooperative Oncology Group Performance Status
*Significant, $p < 0.05$ (bolded); # $0.05 < p < 0.10$ (italics)
Adjustments were not made for multiple comparisons.
Changes in significant variables from original published data: In the original report, sex was a significant covariate in the initial model (HR 2.029; p = 0.038) and in the refined model (HR 1.980; p = 0.038); however, it was erroneously listed in the table as Age instead of Sex. In the revised initial and refined models, sex only trends to significance.

Supplementary Table 8. Cox Regression Model for Overall Survival for Patients receiving 12MP+6MHP vaccine (Arms C + D; n = 85) Revised with corrected data.

Multivariable model for OS for patients on arms C+D (n = 85; p = 0.14, Chi-square 10.97)				
Covariate	***Detail***	***p value***	***HR***	***95% CI of HR***
**Age**	≤43 vs. >43 years**	**0.021***	0.216	0.059 to 0.794
**Sex**	Female vs Male	**0.043***	2.495	1.031 to 6.042
Arm	Arm C vs. D	0.404	1.374	0.652 to 2.895
AJCC stage, v8	Stage IV vs. II-III	0.623	0.750	0.238 to 2.360
Advanced disease status	Recurrence vs initial diagnosis	0.316	0.624	0.249 to 1.568
LDH level	High vs. normal or not done	0.521	1.959	0.251 to 15.26
ECOG Performance Status	1 vs 0	0.700	1.253	0.398 to 3.939

Abbreviations: HR, hazard ratio; CI, confidence interval; AJCC, American Joint Committee on Cancer; LDH, lactate dehydrogenase; ECOG PS, Eastern Cooperative Oncology Group Performance Status

* Significant, $p < 0.05$ (bolded); Adjustments were not made for multiple comparisons.

**Among participants on Arms C and D, there were no mortality events among participants≤40 years old. To enable effective modeling, this analysis was done for ages≤43 years.

Supplementary Table 9. Cox Regression Model for RFS for Arms C + D (n = 85). Revised with corrected data.

Multivariable model for RFS for patients on arms C+D (n = 85) ***Initial model (p = 0.079, Chi-square 12.715)***				
Covariate	***Detail***	***p value***	***HR***	***95% CI of HR***
**Sex**	Female vs Male	**0.014***	2.291	1.183 to 4.436
Age	≤40 vs. >40 years	**0.039***	0.274	0.080 to 0.936
AJCC stage, v8	Stage IV vs II-III	0.520	1.313	0.574 to 3.004
Arm	Arm C vs. D	0.875	1.049	0.578 to 1.904
ECOG Performance Status	1 vs 0	0.609	0.727	0.214 to 2.466
Advanced disease status	Recurrence vs initial diagnosis	0.861	0.939	0.465 to 1.899
LDH	High vs. normal or not done	0.969	1.042	0.138 to 7.867
Refined model (p = 0.003, Chi-squared 11.73)				
Covariate	***Detail***	***p value***	***HR***	***95% CI of HR***
**Sex**	Female vs Male	**0.006***	2.352	1.285 to 4.307
**Age**	≤40 vs. >40 years	**0.028***	0.264	0.081 to 0.865

Abbreviations: HR, hazard ratio; CI, confidence interval; AJCC, American Joint Committee on Cancer; ECOG PS, Eastern Cooperative Oncology Group Performance Status; LDH, lactate dehydrogenase

* Significant, $p < 0.05$ (bolded); Adjustments were not made for multiple comparisons.

Changes in significant variables from original published data: In the model with all covariates, age is significantly associated with RFS for arm C+D (p = 0.039) whereas it only trended to significance in the original report (p = 0.063). In the refined model, both age and sex are significant, as in the original report.

Supplementary Table 10. Cox regression model for Overall Survival for Arms A + B (n = 82). Revised with corrected data.

Multivariable model for OS for patients on Arms A + B (n = 82; p = 0.001, Chi-square 10.56)				
**Covariate**	***Detail***	***p value***	***HR***	***95% CI***
Age	≤ 40 vs > 40 years	**0.028***	0.107	0.015 to 0.782

Abbreviations: HR, hazard ratio; CI, confidence interval; LDH, lactate dehydrogenase; AJCC v8, American Joint Committee on Cancer version 8; ECOG PS, Eastern Cooperative Oncology Group Performance Status
*Significant, $p < 0.05$ (bolded); Adjustments were not made for multiple comparisons.

Supplementary Table 11. Cox regression model for RFS for Arms A + B (n = 82). Revised with corrected data.

Multivariable model for RFS for patients on Arms A + B (n = 82; p = 0.132, Chi-square 5.62)				
**Covariate**	***Detail***	***p value***	***HR***	***95% CI***
Age	≤ 40 vs > 40 years	*0.087*#	0.468	0.196 to 1.117
Arm	Arm A vs Arm B	0.116	1.570	0.894 to 2.754
AJCC stage, v8	Stage IV vs II-III	0.260	0.679	0.346 to 1.332

Abbreviations: HR, hazard ratio; CI, confidence interval; LDH, lactate dehydrogenase; AJCC v8, American Joint Committee on Cancer version 8; ECOG PS, Eastern Cooperative Oncology Group Performance Status
*Significant, $p < 0.05$ (bolded); # $0.05 < p < 0.10$ (italics); Adjustments were not made for multiple comparisons.

Supplementary Table 12. Cox Regression Model for OS for Males only (n = 112). Revised with corrected data.

Multivariable model for OS for male patients (n = 112; p = 0.013, Chi-square 12.7)				
Covariate	***Detail***	***p value***	***HR***	***95% CI of HR***
**Vaccine Regimen**[†]	12MP+tet vs. 12MP+6MHP	**0.032***	2.041	1.065 to 3.912
**Age**	≤43 vs. >43 years	**0.034***	0.277	0.085 to 0.904
AJCC stage v8	Stage IV vs II-III	0.191	0.541	0.216 to 1.357
Advanced Disease Status	Recurrence vs Initial Diagnosis	0.273	0.689	0.353 to 1.342

Abbreviations: HR, hazard ratio; CI, confidence interval; 12MP, 12 class I MHC-restricted melanoma peptides; tet, tetanus toxoid helper peptide; 6MHP, mixture of six melanoma-specific helper peptides; AJCC, American Joint Committee on Cancer; LDH, lactate dehydrogenase; ECOG PS, Eastern Cooperative Oncology Group Performance Status

[†] Vaccine regimen 12MP + tet includes study arms A+B; vaccine regimen 12MP + 6MHP includes study arms C+D

* Significant, p < 0.05 (bolded); Adjustments were not made for multiple comparisons.

**Among male patients, there were no mortality events among participants≤40 years old. To enable effective modeling, this analysis was done for ages≤43 years.

Supplementary Table 13. Cox Regression Model for OS for Females only (n = 55). Revised with corrected data.

Multivariable model for OS for female patients (n = 55; p = 0.008, Chi-square 13.70)				
Covariate	***Detail***	***p value***	***HR***	***95% CI of HR***
**Age**	≤40 vs. >40 years	**0.013***	0.077	0.010 to 0.579
Study Arm	A (12MP+Tet) vs B (12MP+Tet+Cy)	0.102	2.363	0.843 to 6.625
	C vs. B	0.467	1.484	0.512 to 4.300
	D vs B	0.845	1.118	0.365 to 3.429

Abbreviations: HR, hazard ratio; CI, confidence interval; 12MP, 12 class I MHC-restricted melanoma peptides; tet, tetanus toxoid helper peptide; 6MHP, mixture of six melanoma-specific helper peptides;
* Significant, p < 0.05 (bolded); Adjustments were not made for multiple comparisons.

Supplementary Table 14. Cox Regression Models for OS and RFS for stage II-III patients (n = 132). Revised with corrected data.

Refined model (p < 0.0001, Chi-squared = 24.99): OS for stage II-III (n = 132)				
**Covariate**	***Detail***	***p value***	***HR***	***95% CI***
Age	≤ 40 vs > 40 years	**0.006***	0.064	0.009 to 0.460
Vaccine Regimen: 6MHP (yes/no)	12MP+tet vs. 12MP+6MHP	**0.033***	1.787	1.049 to 3.045
Refined model (p = 0.009, Chi-squared = 9.47): RFS for stage II-III (n = 132)				
**Covariate**	***Detail***	***p value***	***HR***	***95% CI***
Age	≤ 40 vs > 40 years	**0.037***	0.458	0.220 to 0.956
Vaccine Regimen: 6MHP (yes/no)	12MP+tet vs. 12MP+6MHP	**0.038***	1.634	1.028 to 2.697

Abbreviations: HR, hazard ratio; CI, confidence interval; 12MP, 12 class I MHC-restricted melanoma peptides; tet, tetanus toxoid helper peptide; 6MHP, mixture of six melanoma-specific helper peptides;
* Significant, p < 0.05 (bolded); Adjustments were not made for multiple comparisons.
Changes in significant variables from original published data: In the refined model for RFS, age is a significant covariate whereas it only trended to significance in the original report (p = 0.067).

Supplementary Note –
Study Protocol

UNIVERSITY OF VIRGINIA
HUMAN IMMUNE THERAPY CENTER

MEL 44
A MULTICENTER TRIAL TO EVALUATE THE EFFECTS OF ADMINISTRATION OF CYCLOPHOSPHAMIDE AND MELANOMA-DERIVED HELPER PEPTIDES ON THE IMMUNOGENICITY OF A CLASS I MHC-RESTRICTED PEPTIDE-BASED VACCINE IN PARTICIPANTS WITH RESECTED MELANOMA

__X__ **Amendment**

_____ Change of participant(s)
_____ Editorial, administrative changes
_____ Eligibility changes
_____ Scientific changes
_____ Therapy changes
_____ Informed consent changes
__X__ Other:

_____ **Status Change**

_____ Activation
_____ Closure
_____ Suspension
_____ Reactivation

Summary:

- Allowing for collection of tissue after participants are removed from study.
- Allowing for collection of tissue of any surgical resections of the disease, not limited to superficial metastases, with participant consent.

UNIVERSITY OF VIRGINIA
HUMAN IMMUNE THERAPY CENTER

MEL44
A MULTICENTER TRIAL TO EVALUATE THE EFFECTS OF ADMINISTRATION OF CYCLOPHOSPHAMIDE AND MELANOMA-DERIVED HELPER PEPTIDES ON THE IMMUNOGENICITY OF A CLASS I MHC-RESTRICTED PEPTIDE-BASED VACCINE IN PARTICIPANTS WITH RESECTED MELANOMA

Principal Investigator: Craig L. Slingluff, Jr., M.D.
Co-Investigators: William W. Grosh, M.D.
Anneke T. Schroen, M.D.
Biostatistician: Gina R. Petroni, Ph.D.
Protocol Development: Kimberly A. Chianese-Bullock, Ph.D.
Scott A. Boerner, M.S.

Protocol Precis

Title: A Multicenter Trial to Evaluate the Effects of Administration of Cyclophosphamide and Melanoma-Derived Helper Peptides on the Immunogenicity of a Class I MHC-Restricted Peptide-Based Vaccine in Participants with Resected Melanoma (Mel 44)

Goals:
Safety
(1) to determine whether the administration of 12 melanoma peptides comprised of class I MHC-restricted epitopes (12-MP) in conjunction with 6 melanoma-derived class II MHC-restricted helper peptides (6-MHP) is safe
(2) to determine whether the administration of cyclophosphamide (Cytoxan ®) prior to administration of a peptide-based vaccine is safe

Immunogenicity
Primary:
(1) to determine the magnitude of immune responses against the 12-MP when administered in conjunction with a tetanus toxoid-derived helper peptide or 6-MHP with and without the addition of cyclophosphamide (Group C>Group A, Group D>Group B, Group D>Group C, Group B>Group A)

Secondary:
(1) to determine the response rate and persistence of immune responses against the 12-MP when administered in conjunction with a tetanus toxoid-derived helper peptide or 6-MHP with and without the addition of cyclophosphamide
(2) to determine the magnitude of immune responses against the tetanus toxoid-derived helper peptide or 6-MHP with and without the addition of cyclophosphamide
(3) to determine the response rate and persistence of immune responses against the tetanus toxoid-derived helper peptide or 6-MHP with and without the addition of cyclophosphamide
(4) to determine DTH responses to the peptide components of the vaccine
(5) to obtain preliminary estimates of disease-free survival

Design: This is an open-label, multicenter phase I/II study of a vaccine comprised of a mixture of 12-MP administered in Montanide ISA-51. Participants will be randomized to receive the 12-MP in combination with a tetanus toxoid-derived helper peptide with or without a single pre-treatment with cyclophosphamide or to receive the 12-MP in combination with a 6-MHP mixture with or without a single pre-treatment with cyclophosphamide.

Regimen: Vaccines will be administered over a 1-year period (days 1, 8, 15, 29, 36, 43, and weeks 12, 26, 39, and 52). Participants will be randomized into one of four groups:

**Group A (12-MP + tet):**	12-MP administered with a tetanus toxoid-derived class II MHC-restricted helper peptide
**Group B (Cy + 12-MP + tet):**	Cyclophosphamide followed by vaccination with the 12-MP administered with a tetanus toxoid-derived class II MHC-restricted helper peptide
**Group C (12-MP + 6-MHP):**	12-MP administered with 6-MHP

Group D (Cy + 12-MP + 6MHP): Cyclophosphamide followed by vaccination with the 12-MP administered with 6-MHP

Cyclophosphamide (300 mg/m^2) will be administered intravenously in 250 ml of saline over 30-60 minutes 5 days prior (Day -4) to administration of the first vaccine (Day 1). Participants will receive up to 24 mg of Zofran prior to administration of cyclophosphamide.

All peptide vaccines will be administered intradermally and subcutaneously.

Population: Criteria for inclusion include age 18 years or older, expression of HLA-A1, -A2, or -A3 and HLA-DR1, -DR4, -DR11, -DR13, or -DR15, and the diagnosis of resected high-risk melanoma (stage IIB-IV). The maximum number of participants accrued will be 173.

Vaccine composition: The peptides described in Tables 1-3 will be used in the vaccines.

Table 1: 12-MP

Allele	Sequence	Epitope
HLA-A1	DAEKSDICTDEY	Tyrosinase $_{240-251}$ *
	SSDYVIPIGTY	Tyrosinase $_{146-156}$
	EADPTGHSY	MAGE-A1 $_{161-169}$
	EVDPIGHLY	MAGE-A3 $_{168-176}$
HLA-A2	YMDGTMSQV	Tyrosinase $_{369-377}$ ♦
	IMDQVPFSV	gp100 $_{209-217}$ #
	YLEPGPVTA	gp100 $_{280-288}$
	GLYDGMEHL	MAGE-A10 $_{254-262}$
HLA-A3	ALLAVGATK	gp100 $_{17-25}$
	LIYRRRLMK	gp100 $_{614-622}$
	SLFRAVITK	MAGE-A1 $_{96-104}$
	ASGPGGGAPR	NY-ESO-1 $_{53-62}$

*****(substitution of S for C at residue 244)
♦(post-translational change of N to D at residue 371)
#(209-2M, substitution of M for T at residue 210)

Table 2: 6-MHP

Allele	Sequence	Epitope
HLA-DR4	AQNILLSNAPLGPQFP	Tyrosinase $_{56-70}$#
HLA-DR15	FLLHHAFVDSIFEQWLQRHRP	Tyrosinase $_{386-406}$
HLA-DR4	RNGYRALMDKSLHVGTQCALTRR	Melan-A/MART-1$_{51-73}$
HLA-DR11	TSYVKVLHHMVKISG	MAGE-3 $_{281-295}$
HLA-DR13	LLKYRAREPVTKAE	MAGE-1,2,3,6 $_{121-134}$
HLA-DR1 & -DR4	WNRQLYPEWTEAQRLD	gp100 $_{44-59}$

An alanine residue was added to the N-terminus to prevent cyclization

Table 3: Tetanus toxoid-derived helper peptide

Allele	Sequence	Epitope
Binds to multiple class II alleles	AQYIKANSKFIGITEL	p2$_{830-844}$**

**An alanine residue was added to the N-terminus to prevent cyclization

Each group will receive the following:

Group A (12-MP + tet)

- Days 1, 8, 15, 29, 36, 43 and weeks 12, 26, 39, 52: 100 mcg each of the 12 peptides listed in Table 1 and 200 mcg of the tetanus toxoid peptide listed in Table 3 emulsified in Montanide ISA-51 adjuvant. The vaccine will be divided and administered at two sites, the primary and replicate vaccination sites, which are located on two different extremities. The vaccine will be administered subcutaneously and intradermally at both vaccination sites.

Group B (Cy + 12-MP + tet)

- Day -4: 300 mg/m^2 of cyclophosphamide administered once intravenously in 250 ml of saline over 30-60 minutes.
- Days 1, 8, 15, 29, 36, 43 and weeks 12, 26, 39, 52 : 100 mcg each of the 12 peptides listed in Table 1 and 200 mcg of the tetanus toxoid peptide listed in Table 3 emulsified in Montanide ISA-51 adjuvant. The vaccine will be divided and administered at two sites, the primary and replicate vaccination sites, which are located on two different extremities. The vaccine will be administered subcutaneously and intradermally at both vaccination sites.

Group C (12-MP + 6-MHP)

- Days 1, 8, 15, 29, 36, 43 and weeks 12, 26, 39, 52: 100 mcg each of the 12 peptides listed in Table 1 and 200 mcg of each of the 6 peptides listed in Table 2 emulsified in Montanide ISA-51 adjuvant. The vaccine will be divided and administered at two sites, the primary and replicate vaccination sites, which are located on two different extremities. The vaccine will be administered subcutaneously and intradermally at both vaccination sites.

Group D (Cy + 12-MP + 6-MHP)

- Day -4: 300 mg/m^2 of cyclophosphamide administered intravenously in 250 ml of saline over 30-60 minutes.
- Days 1, 8, 15, 29, 36, 43 and weeks 12, 26, 39, 52: 100 mcg each of the 12 peptides listed in Table 1 and 200 mcg of each of the 6 peptides listed in Table 2 emulsified in Montanide ISA-51 adjuvant. The vaccine will be divided and administered at two sites, the primary and replicate vaccination sites, which are located on two different extremities. The vaccine will be administered subcutaneously and intradermally at both vaccination sites.

Endpoints:
Safety:

1. Evaluation of the safety of administration of the vaccines as measured by the rate of dose-limiting toxicities.

Immunogenicity:
Primary

1. Cytotoxic T cell responses to the 12-MP over the first six vaccines.

Secondary

2. Proliferative responses of T cells to the tetanus helper peptide and 6-MHP over the first six vaccines.
3. DTH responses to the peptides.
4. Cytotoxic T cell responses to the 12-MP and helper T cell responses to the 6-MHP over the booster vaccines.
5. Disease-free survival.

TABLE OF CONTENTS

List of Abbreviations ... 9
1.0 INTRODUCTION ... 12
Abstract ... 12
1.1 Study Rationale ... 13
1.2 Vaccine Design ... 20
1.2.1 Antigen-Specific Immune Responses Induced by Peptide Vaccines ... 20
1.2.2 Melanoma Peptides Restricted by MHC Class I Molecules and Incorporated in the 12-Melanoma Peptide Mixture ... 21
1.2.3 Tetanus Toxoid Helper Peptide ... 24
1.2.4 Melanoma Peptides Restricted by MHC Class II Molecules and Incorporated in the 12-Melanoma Peptide Vaccine ... 25
1.2.5 Montanide ISA-51 as a Vaccine Adjuvant ... 25
1.2.6 Integration of Peptide-Based Vaccines with Interferon Therapy ... 26
1.2.7 Toxicology ... 26
1.2.8 Dosing ... 28
1.2.9 Regulatory Issues ... 29
2.0 STUDY OBJECTIVES ... 29
3.0 STUDY OUTLINE ... 30
3.1 Type of Study ... 30
4.0 PARTICIPANT SELECTION ... 30
4.1 Inclusion Criteria ... 30
4.2 Exclusion Criteria: ... 31
4.3 Demographics of Participant Population ... 33
4.4 Registration, Randomization, and Management of Participants ... 33
4.4.1 Participant Registration ... 33
4.4.2 Randomization ... 33
4.4.3 Management of Participants ... 33
5.0 STUDY MEDICATION FORMULATION, DOSAGE, AND ADMINISTRATION ... 33
5.1 Study Medication Formulation ... 33
5.1.1 Peptide Synthesis and Storage ... 33
5.1.2 Reconstitution and Vialing of Peptides ... 34
5.1.3 Storage of Vialed Peptides ... 34
5.1.4 Lot Testing ... 34
5.1.5 Labeling ... 34
5.1.6 Purchase and Storage of Cyclophosphamide ... 34
5.2 Preparation, Dosage, and Storage of Study Drug ... 34
5.2.1 Dosage and Preparation of Cyclophosphamide (Groups B and D) ... 34
5.2.2 Dosage and Preparation of Peptide Vaccine ... 35
5.2.3 Storage of Prepared Peptide Vaccines ... 35
5.3 Administration of Peptide Vaccines ... 35
5.3.1 Designation of Vaccine Sites ... 35
5.3.2 Regimen ... 35
5.3.3 Post-Vaccination Observation ... 36
5.4 Modification/Discontinuation of Treatment ... 36
5.4.1 Dose Modifications ... 36
5.4.2 Discontinuation ... 36
5.4.3 Elective Withdrawal ... 37
5.4.4 Delayed Visit for Reasons Other than Toxicity ... 37

5.5 Concomitant Medications 38
5.5.1 Non-permitted medications or treatments 39
5.5.2 Permitted medications or treatments 39
6.0 CLINICAL AND LABORATORY EVALUATION 39
6.1 Screening 39
6.2 Treatment 40
6.3 Follow-up 42
6.4 Sample Submission of Blood and Tissue for Immunologic Testing 43
6.4.1 Sample Submission Schedule 43
6.4.2 Sample Preparation Guidelines 44
6.4.3 Shipping Procedures 44
7.0 REGULATORY AND REPORTING REQUIREMENTS 44
7.1 Risks and Safety 44
7.1.1 Agent-Specific Expected Adverse Events List: 45
7.1.2 Reporting of Subject Withdrawals/Dropouts Prior to Study Completion . 46
7.2 Adverse Event Reporting: 46
7.2.1 Definitions 46
7.2.2 Reporting of AEs 49
7.3 Adverse Event Review and Monitoring 52
7.5 Responsibility 54
7.6 Endpoint Data 55
8.0 EVALUATION OF IMMUNOLOGIC RESULTS 55
8.1 ELIspot assay 55
8.2 Tetramer assay 55
8.3 Enzyme-Linked Immunosorbent Assay (ELISA) 55
8.4 Proliferation assay 55
8.5 Evaluation of tumor 55
9.0 STATISTICAL CONSIDERATIONS 55
9.1 Accrual 56
9.2 Safety 56
9.3 Immunogenicity Endpoints 57
9.4 Sample Size Determination and Hypothesis Testing 57
9.5 Analyses 58
APPENDICES 60
Appendix 1: Clinical trial flow diagram and X-page 61
Appendix 2: AJCC Staging System 63
Appendix 3: ECOG Performance Status 65
Appendix 4: New York Heart Association Disease Classification 66
Appendix 5: Lot Testing 67
Appendix 6: Vaccine Preparation 69
Appendix 7: NCI Common Terminology Criteria for Adverse Events v3.0 81
Appendix 8: Immunologic Studies 82

List of Abbreviations

Abbreviation	Full text
β–HCG	Beta Human chorionic gonadotropin (pregnancy test)
6-MHP	6 melanoma-derived class II MHC-restricted helper peptides
12-MP	12 melanoma-derived class I MHC-restricted peptides
AE	adverse event
AGC	absolute granulocyte count
AJCC	American Joint Committee on Cancer
ALT	alanine aminotransferase
ANC	absolute neutrophil count
APC	antigen presenting cell
AST	aspartate aminotransferase
BSA	body surface area
BUN	urea nitrogen
CC	Cancer Center
cc	cubic centimeter
CFR	Code of Federal Regulations
cm	centimeter
CO_2	carbon dioxide
CRF	case report form
CT	computed tomography
CTA	cancer-testis antigens
CTCAE	Common Terminology Criteria for Adverse Events
CTL	cytotoxic T lymphocyte
CTO	Clinical Trials Office
Cy	cyclophosphamide
DC	dendritic cells
dL	deciliter
DLT	dose limiting toxicity
DSMC	Data and Safety Monitoring Committee
DTH	delayed type hypersensitivity
ECOG	Eastern Cooperative Oncology Group
ELISA	enzyme linked immunosorbent assay
FACS	fluorescence activated cell sorter
FBS	fetal bovine serum
FDA	Food and Drug Administration
FITC	Fluorescein isothiocyanate
g	gram
GCRC	General Clinical Research Center
GM-CSF	granulocyte-macrophage stimulating colony
GMP	good manufacturing practice
Hgb	hemoglobin
HGBA1C	hemoglobin a1c
HITC	Human Immune Therapy Center
HIV	human immunodeficiency virus
HLA	human leukocyte antigen
HPLC	high performance liquid chromatography
IBW	ideal body weight
id	intradermal
IFN	interferon
IL-2	interleukin-2
IL-7	interleukin-7

IL-15	interleukin-15
IML	Immune Monitoring Laboratory
In.	inch
IND	investigational new drug
ip	intraperitoneal
IRB	Institutional Review Board
IU	international unit
IV	intravenous
kg	kilogram
KLH	keyhole limpet hemocyanin
LC	Langerhans cells
LDH	lactate dehydrogenase
Lf	flocculation value
m	meter
mcCi	microcurie
mcg	microgram
mcl	microliter
MDP	melanocyte differentiation proteins
mg	milligram
MHC	major histocompatability complex
mIU	million international units
ml	milliliter
mm	millimeter
MRI	magnetic resonance imaging
NBT/BCIP	Nitro blue tetrazolium chloride/5-Bromo-4-Chloro-3-Indolyl Phosphate
NCI	National Cancer Institute
NOS	not otherwise specified
NSAID	non-steroidal anti-inflammatory drug
OCS	Office of Collaborative Studies
PAP	pulmonary alveolar proteinosis
PBL	peripheral blood lymphocytes
PBS	phosphate buffered saline
PET	positron emission tomography
PHA	phytohemagglutinin
PI	principal investigator
PMA	phorbol myristate acetate
ppm	parts per million
PRC	protocol review committee
RPMI	Roswell Park Memorial Institute
sc	subcutaneous
SD	standard deviation
SIN	sentinel immunized node
TAA	tumor associated antigens
tet	tetanus helper peptide
TFA	trifluoroacetic acid
T_h	$CD4^+$ helper T cells
TIL	tumor infiltrating lymphocytes
TNF	tumor necrosis factor
TPF	Tissue Procurement Facility
TU	tuberculin units
ULN	upper limits of normal
UPCI	University of Pittsburgh Cancer Institute
USP	United States Pharmacopeia

UVA	University of Virginia
WBC	white blood cell
w/v	weight to volume
v/v	Volume to volume

1.0 INTRODUCTION

Abstract
The incidence of melanoma has been increasing steadily over the past 50 years. At the present time, approximately 1 in 70 individuals will be diagnosed with melanoma during their lifetime. Existing therapy for melanoma consists of adequate surgical resection of the primary tumor and, in some circumstances, surgical resection of isolated metastases. After surgical resection of high-risk melanoma, the only approved adjuvant therapy is high-dose interferon (IFN). Unfortunately, some patients are not candidates for, refuse, or fail IFN therapy. Even with high-dose IFN therapy, most patients with high-risk melanoma still progress and die of their disease. Thus, additional therapy for use in the management of melanoma in the adjuvant setting is needed. Immunotherapeutic approaches offer a potentially useful treatment for these patients. An important foundation for such approaches is the targeting of host immune responses against specific tumor antigens.

The majority of shared melanoma antigens identified thus far can be divided into two groups: 1) melanocyte differentiation proteins (MDP) (e.g. MART-1/MelanA, gp100/Pmel17, tyrosinase, TRP-1/gp75 and TRP-2) and 2) cancer-testis antigens (CTA) (e.g. MAGE proteins GAGE-1/2, BAGE, RAGE, and NY-ESO-1). Generally, both types of proteins are encoded by non-mutated genes, which lack tumor-specific mutations. Therefore, epitopes derived from these molecules could be useful in a vaccine for a large population of melanoma patients. Interestingly, MDP and CTA are differentially expressed in various stages of melanoma. Generally, the expression of MDP decreases in more metastatic lesions, whereas the expression of CTA increases in metastatic lesions (1-3). Thus, metastatic tumors contain a heterogeneous population of cells with respect to protein expression. Based on the emergence of metastases that have lost expression of target antigens, vaccines incorporating single melanoma derived epitopes may be inadequate in generating a complete immune response against the tumor (4,5). Ideally, a polyvalent vaccine incorporating epitopes derived from both groups of antigens should compensate for the differential display of melanoma-associated antigens.

The vast majority of peptide-based cancer vaccines initiated to date worldwide have incorporated class I MHC-restricted epitopes to activate the $CD8^+$ cytotoxic T cell arm of the host immune system. However, activation of tumor-specific $CD4^+$ helper T cells (T_h) may also be critical for the elimination of tumor. In past studies, a helper peptide derived from a tetanus toxoid protein was included in the vaccine in an effort to activate the T helper arm of the immune system. However, with the recent identification of class II MHC-restricted helper epitopes derived from melanoma-associated proteins, evaluating whether activation of a T_h subset directly against melanoma antigens augments cytotoxic T cell responses to the melanoma vaccine is important. The present study is designed to address whether melanoma-derived class II MHC-restricted helper epitopes included in the vaccine regimen augment the magnitude of $CD8^+$ T cell responses and contribute to the development of persistent cytotoxic T cell responses to the class I MHC-restricted peptides in the vaccine.

A second question being addressed is whether pre-treatment with a single dose of cyclophosphamide prior to vaccination enhances the immunogenicity of a peptide-based vaccine. When administered at a lower dose compared to that used for tumor

lytic agents, cyclophosphamide has been shown to augment immune responses. Thus, we propose to test the hypothesis that pre-treatment with low-dose cyclophosphamide will lead to persistent cytotoxic and T_h responses to the class I and class II MHC-restricted epitopes, respectively, in the vaccine.

This study is unique among peptide vaccine trials proposed to date in that (a) both class I and class II MHC-restricted peptides derived from MDP and CTA are incorporated in the vaccine, (b) it will test whether inclusion of melanoma-derived class II MHC-restricted helper peptides augments $CD8^+$ T cell responses and affects the persistence of cytotoxic T cell responses against the class I MHC-restricted peptides included in the vaccine, and (c) it will evaluate the effects of cyclophosphamide on the immunogenicity of a peptide-based vaccine.

1.1 Study Rationale

Evidence for the Role of the Immune System in Protecting Against the Development of Solid Tumors

There has long been evidence of immune responses to cancer, but evidence of impact on tumor progression has not been well demonstrated until recently. The most convincing evidence of the importance of immune surveillance in preventing the development of solid tumors is provided by recent work in murine models, in which 50-100% of knockout mice lacking STAT1 and/or IFNγ receptor function developed spontaneous solid tumors of various histologies within 12-15 months, whereas normal mice never developed malignancies during the same time period (6). These studies strongly support the role of cellular immune function in the control of cancer progression.

The Role of $CD8^+$ Cytotoxic T Lymphocytes in Anti-tumor Immune Responses

Approaches to induce tumor-specific immune responses have focused on identifying efficient ways to activate and to amplify $CD8^+$ T cell populations directed against tumor antigens. These investigations have focused on the activation of $CD8^+$ cytotoxic T lymphocytes (CTL) because of their well documented cytolytic and cytokine secreting activities. Therefore, tumor cells expressing MHC class I may be eliminated from the host following recognition by CTL. Supporting this notion is evidence anti-tumor immune responses are compromised in a murine model following the elimination of $CD8^+$ T cells (7-10). Further, $CD8^+$ T cell populations raised against tumor antigens *in vitro* are capable of eliminating tumor in a tumor-bearing host after adoptive transfer (11,12). Therefore, the identification of the antigenic targets of $CD8^+$ CTL has been of great benefit for the design and development of active specific immune therapies directed toward the *in vivo* activation of tumor-specific T cells.

Selection of Class I MHC-restricted Epitopes for Melanoma-reactive CTL

More than 50 melanoma peptides have been characterized and these peptides are presented by a variety of HLA alleles, including HLA-A, -B and -C alleles (13). Peptides derived from tumor-associated antigens (TAA) have been identified, synthesized, and used *in vitro* and *in vivo* to elicit tumor-reactive T-cell responses (14). The identification of peptide epitopes for melanoma-specific CTL provides an opportunity to create novel vaccines that can be evaluated directly in terms of the CTL response to the purified immunizing antigen. In this regard, in recent clinical trials,

epitope-specific immunological responses against peptides incorporated into a melanoma vaccine were detected at various time points post-vaccination (4,15-17).

In a recently completed clinical trial (UVA-Mel39), we assessed the immunogenicity of the 12-MP vaccine described in Table 1. The 12-MP vaccine includes 7 peptides (DAEKSDICTDEY, EADPTGHSY, EVDPIGHLY, YMDGTMSQV, IMDQVPFSV, YLEPGPVTA, ALLAVGATK) reported by us or others to be immunogenic and safe in humans (4,16,18,19), plus 5 peptides (SSDYVIPIGTY, GLYDGMEHL, LIYRRRLMK, SLFRAVITK, ASGPGGGAPR) defined as epitopes for melanoma-reactive CTL, but not previously evaluated for immunogenicity or safety in humans until UVA-Mel39 (20-23). Twenty-five participants were immunized with the 12-MP vaccine. Based on a preliminary assessment of the data, T cell responses were generated against 10 of the peptides in the mixture (DAEKSDICTDEY, EVDPIGHLY, YMDGTMSQV, YLEPGPVTA, IMDQVPFSV, GLYDGMEHL, ALLAVGATK, LIYRRRLMK, SLFRAVITK, ASGPGGGAPR) without serious, unexpected toxicity.

Because cell-mediated immunity is believed to be critical for tumor rejection, a melanoma vaccine should induce potent CTL responses specific for melanoma cells from a majority of patients at risk. Two criteria are important to consider when selecting peptides for a vaccine intended to be used in the majority of the melanoma patient population. The first is the frequency of expression of the parent protein from which the epitope is derived. The 12-MP incorporates epitopes derived from MDP and CTA, which are expressed at moderate to high frequency levels at various stages of disease. The second is the frequency of expression of the HLA alleles to which the epitopes are restricted. The proposed trial will incorporate peptide epitopes presented by HLA-A1, -A2 (A*0201), and -A3 molecules and the percentage of the melanoma patient population expressing at least one of these HLA alleles is approximately 80%.

MHC Restriction and HLA Superfamilies

Although epitopes for CTL have been defined principally in association with individual class I MHC molecules, there is growing evidence class I-restricted epitopes can bind additional MHC molecules within an HLA superfamily (24). The A3 supertype has several members including HLA-A3, -A11, -A31, -A33, and -A68. The NY-ESO-1_{53-62} epitope was identified by Wang et al. (22) and was shown to be restricted by HLA-A31. Using mass spectrometry, we found this peptide associated with HLA-A3 on both the VMM12 and VMM18 human melanoma cell lines (manuscript in preparation); therefore, it was included in the 12-MP mixture. Preliminary immunologic data from the UVA-MEL39 trial has shown this peptide is immunogenic when administered as part of the 12-MP (+ tet) vaccine in 30% of participants who express HLA-A3.

Competition Between Peptides for Binding to the Same MHC Class I Molecule

One concern regarding the effectiveness of a multi-peptide vaccine is whether competition by peptides with high to intermediate affinity for the MHC class I molecule can inhibit the generation of an immune response against a lower affinity peptide binding to the same MHC molecule. If competition interferes with the immunogenicity of a low-affinity peptide, a multivalent vaccine would need to be administered in multiple vaccination sites, with different affinity peptides restricted by the same class I allele administered in separate sites. This might be feasible for a vaccine incorporating 3- 4 peptides restricted by the same MHC class I molecule. However, the number of immunogenic epitopes derived from melanoma-associated antigens is

growing, and the idea of administering a multivalent vaccine at several sites becomes increasingly less feasible with increasing numbers of peptides. Thus, an important question to be tested directly is whether the immune response to a single peptide is increased, maintained, or decreased, when that peptide is administered in a mixture with other MHC-associated peptides, compared to administration of each peptide separately.

There is evidence competition between different affinity peptides for binding to the same MHC class I molecule may not necessarily render a low-affinity peptide non-immunogenic. First, in the case of short term pulsing, peptide-MHC complexes form independent of the binding affinity of the peptide (25). In this situation, surface antigen densities are the result of the peptide on-rate, which is similar for peptides of the same size pulsed onto APC at the same concentration (25). Surface antigen density, however, declines once the exogenous source of peptide is removed. Unlike the formation of peptide-MHC complexes, the rate of decline in antigen density is dependent on the affinity of the peptide for the class I molecule, and is defined by the off-rate for each peptide (25). These findings strongly suggest co-administration of four peptides with varied binding affinities at equimolar concentrations will result in a ¼ reduction of each type of MHC-peptide complex, assuming the peptides are added in saturating quantities. One consequence of using low affinity peptides, however, is these peptides may disassociate at a faster rate than high-affinity peptides with MHC class I molecules en route to the lymph node, prior to interacting with the appropriate T cell. Nevertheless, T-cells can recognize and respond to 100 copies of peptide per cell, or less; thus, these results suggest competition among the four peptides for binding to each MHC class I molecule may not be a factor in determining the immunogenicity of each individual peptide.

Second, we performed an *in vitro* study to identify whether an intermediate-affinity melanoma peptide (YMDGTMSQV, tyrosinase) would compete with a low-affinity melanoma peptide (YLEPGPVTA, gp100) for binding to the HLA-A2 class I molecule and prevent recognition of the low-affinity peptide by CTL. *In vitro* analyses indicated adding equimolar amounts of the intermediate-affinity peptide had no effect on the relative lytic activity of the T cell population for the YLEPGPVTA peptide-pulsed cells. Therefore, competition for MHC class I binding between the two peptides when present in equimolar concentrations did not interfere with their antigenicity *in vitro* (manuscript submitted).

Third, in ongoing and recently completed vaccine trials, participants were immunized with a mixture of four peptides including the two HLA-A2-restricted peptides YLEPGPVTA (gp100) and YMDGTMSQV (tyrosinase). At least two participants generated an immune response against the YLEPGPVTA peptide without a response against the higher affinity HLA-A2-restricted YMDGTMSQV peptide. These results suggest peptides with an intermediate binding affinity for a class I allele (e.g. YMDGTMSQV) may not interfere with the immunogenicity of peptides with a low binding affinity for the same class I allele when administered at equimolar amounts in the same vaccine mixture.

Lastly, preliminary data from UVA-Mel39 suggest the immunogenicity of low-affinity peptides is not compromised when low- and high-affinity peptides are administered as part of the same peptide mixture (12-MP vaccine).

The Role of $CD4^+$ Helper T Lymphocytes in Anti-tumor Immune Responses

Initially, the majority of cancer vaccines were designed to activate the $CD8^+$ cytotoxic T cell arm of the host immune system. However, more recent approaches target the activation of $CD4^+$ T_h cells. This is based in part on results from earlier studies which demonstrated depletion of $CD4^+$ T-cells abrogates all or part of protective immune response to vaccines (26). Furthermore, adoptive therapy with $CD4^+$ T-cells has been shown to induce tumor protection in some model systems (27). Thus, the protective immunity induced by tumor cell vaccines appears to be mediated both by $CD8^+$ T-cells and by $CD4^+$ T-cells.

Natural immune responses to pathogens consist of an integrated response including T_h responses to epitopes presented by class II MHC molecules and CTL responses to epitopes presented by class I MHC molecules (28). T_h cells can activate dendritic cells (DC) for heightened antigen presentation, causing the DC to secrete IL-2 and other cytokines that may help to direct the immune response. Furthermore, strong T_h1 help produces the proper cytokine milieu which is critical to the induction of immune-mediated tumor destruction (29,30). In addition, T_h responses are believed to be involved in the establishment of memory responses. In the current protocol we will test whether the addition of MDP- and CTA-derived class II MHC-restricted peptides and activation of T_h responses augment the persistence of CTL responses to the class I MHC-restricted peptides in the vaccine.

Selection of Class II MHC-restricted Epitopes for Melanoma-reactive T_h Cells

Until recently, no melanoma-derived class II MHC-restricted epitopes had been identified for use in peptide-based vaccines. Thus, prior peptide-based studies conducted by the UVA HITC incorporated a modified class II MHC-restricted epitope derived from a tetanus toxoid protein (AQYIKANSKFIGITEL) ((17,31) and manuscript submitted). This epitope was included to induce systemic T_h responses that would promote the development of CTL responses against the melanoma-specific class I MHC-restricted peptides in the vaccine. The AQYIKANSKFIGITEL peptide induced a T_h1-type T-helper response readily detectable following vaccination (17,31).

In the current protocol we are including class II MHC-restricted peptides derived from melanoma proteins in an effort to generate melanoma-specific T_h responses. One of the goals of the proposed study is to evaluate immune responses against the 12-MP vaccine when administered in conjunction with a tetanus toxoid-derived helper peptide or the 6-MHP. We hypothesize T_h responses to the class II MHC-restricted melanoma peptides will augment immunologic responses to the 12-MP mixture.

Like the class I MHC-restricted peptides, the melanoma specific class II MHC-restricted peptides (Table 2) are derived from MDP and CTA. The peptides chosen bind to HLA-DR1, -DR4, -DR11, -DR13, and/or -DR15, and approximately 98% of the melanoma patient population will express at least one of the required class II alleles.

The UVA HITC is currently evaluating the safety and immunogenicity of the 6-MHP mixture in a Phase I/II study (UVA-MEL41). Thus far, seven participants have been vaccinated with 200 mcg each of the peptides in the 6-MHP mixture, the same dose selected for the proposed study. Minimal toxicities have been reported and studies evaluating the immunogenicity of the 6-MHP are currently underway.

Cyclophosphamide as an Immune Modulatory Reagent
Cyclophosphamide (Cytoxan®) is a prodrug whose active product interferes with cellular activity through the alkylation of DNA. When administered at a dose inferior to that used for tumor lysis, cyclophosphamide has been shown to augment immune responses in mice and humans (32-35) (36). Multiple mechanisms may be involved in this augmentation, including the amplification of T_h1 type responses (35,37), the down-regulation of IL-10 (38), induction of type I IFN (39), and the deletion of regulatory/suppressor T cells (40-43).

Additionally, augmentation of immune responses following treatment with cyclophosphamide may result from the deletion of lymphoid subsets followed by the induction of homeostatic expansion. Vaccination during the course of homeostatic proliferation may lead to an increase in the number of vaccine-specific T cells. In a recent clinical study by Dudley et al., tumor-reactive autologous T-cell clones were adoptively transferred to participants following administration of a nonmyeloablative dose of cyclophosphamide and fludarabine, which was effective at inducing lymphopenia (44,45). Unlike prior adoptive transfer studies, T-cell clones transferred after treatment with the nonmyeloablative dose of chemotherapy expanded and persisted *in vivo* and objective clinical responses were reported in 6 of 13 participants (45). These results support the notion that vaccinating in an environment supporting T cell proliferation is beneficial for the expansion and maintenance of vaccine-specific effector cells.

Prior studies have investigated the potential immunomodulatory properties of cyclphosphamide over a wide range of doses (75 mg/m^2-1000 mg/m^2) (32,36,41,42,46). In participants with renal cell carcinoma, pre-treatment with 100 mg/m^2 of cyclophosphamide prior to administration of an autologous cell-based vaccine induced partial clinical responses in 4 of 8 participants evaluated at this dose level (36). Specifically, for participants with melanoma, pre-treatment with a dose of 300 mg/m^2 of cyclophosphamide was shown to augment delayed type hypersensitivity (DTH) responses to a model antigen, keyhole limpet hemocyanin (KLH) (33), and an autologous melanoma cell-based vaccine with minimal toxicity reported (e.g., local inflammatory response) (32).

A substantial amount of data supporting the use of a dose of 300 mg/m^2 of cyclophosphamide prior to vaccination to augment immune responses to vaccines is now available. Thus, we have chosen this dose of cyclophosphamide to be administered prior to administration of the peptide mixtures in the proposed study. While we do not expect this dose to induce severe lymphopenia, it may lead to a reduction in the number of select lymphocyte populations, such as regulatory T cells (41,47), resulting in the induction of T cell proliferation to maintain lymphoid homeostasis. Two cytokines, IL-7 and IL-15, have been shown to be important in mediating the expansion of naïve T lymphocytes and the maintenance of memory T cell populations, respectively (48-51). Thus, as part of the proposed study we will monitor levels of these two cytokines in particular to determine if reductions in lymphocyte subsets correlate with an increase in IL-7 and IL-15, and the induction of T cell expansion.

While prior studies have established 300 mg/m^2 as a dose of cyclophosphamide

augmenting immune responses to vaccine therapies, our study is unique in that we will be evaluating the effects of this dose of drug in the setting where specific immune responses against target antigens can be quantified and measured. Prior studies have relied on *in vivo* measures against crude antigens (cell-based mixtures) that are not quantitative (DTH responses) in small numbers of participants (32,33,36). Furthermore, although prior studies suggested cyclophosphamide selectively depletes suppressor T cell populations, different phenotypic markers than those currently in place were used to describe the suppressor population/regulatory population (41-43). In the proposed study, we plan to evaluate the effect of a cyclophosphamide dose of 300 mg/m^2 on the more recently characterized CD4$^+$CD25$^+$ regulatory T cell subset.

Immune Monitoring with Combinations of Functional and Molecular Assays – Assays of CD8$^+$ T-cells

An essential step in the development of effective cancer vaccines is identification of the immune response parameters that effectively measure relevant immunologic endpoints, such as immunogenicity. Ideally, these endpoints will be associated with clinical response. A number of immunologic assays have been evaluated over the years for their ability to serve as sensitive and reliable tools for immune monitoring purposes.

One method for measuring epitope-specific CTL responses is to stain T-cells directly for expression of antigen-specific T-cell receptors using tetramers of MHC-peptide complexes (52). An advantage associated with using tetramer complexes to analyze MHC-peptide specific T-cell populations is that T cells specific for an MHC-peptide complex do not need to be activated, nor do they need to proliferate, in order to be detected. Thus, T cells specific for an MHC-epitope complex can be detected, without the need for prior *in vitro* expansion and, the native functional state of the antigen-specific T cells can be assessed. However, enumeration of T cells using tetramers can include anergic T cells which may not be useful for tumor cell destruction. Notably, inactivated tumor antigen-specific T cells have been detected by tetramers in the peripheral blood of patients with metastatic melanoma (53).

Other limitations associated with use of tetramers are logistical. First, different tetramer complexes need to be synthesized for each antigen that is studied. Second, some peptides bind to their appropriate HLA molecule with a low affinity, which can interfere with tetramer synthesis or stability. Lastly, the quantitation of epitope-specific T cells is avidity dependent. Low-avidity T cells specific for the tetramer complex may not be detected, which would result in an underestimation of the responding T cell population. This is especially relevant when investigating immune responses to tumor antigens, as a majority of these antigens are self-antigens and tolerance to these epitopes could deleteriously impact the range of avidities that are available in a responding T cell population. However, the therapeutic relevance of low-avidity T cells not detected by tetramer is still under debate.

The ELIspot assay was derived to evaluate functional antigen-specific responses by permitting direct counting of T-cells reacting to antigen by production of IFNγ or other cytokines (54-57). Cytotoxic T-cells that are not anergized should secrete IFNγ after exposure to their cognate antigen, especially if they have a memory phenotype (58). ELIspot assays can reproducibly detect functional CTL responses to defined antigens at levels below 0.01% (54,56), and they do not require prolonged *in vitro* culture prior

to evaluation. However, after a single *in vitro* sensitization and 14 day culture, we have found that ELIspot assays are more sensitive and have a higher signal-to-noise ratio than ELIspot assays performed directly *ex vivo* (59,60).

We found that CTL reactive to the gp100 peptide ALLAVGATK were detected by ELIspot at significant levels when those CTL were present above 80 per 100,000 $CD8^+$ cells (0.08%) (59). We found intracellular staining for IFNγ provided results that corroborated those obtained with ELIspot. The former approach permitted dual staining with cell surface markers such as CD8, but ELIspot was more sensitive (59). However, once the frequency of reactive CTL was above a threshold level of about 0.2% of mononuclear cells, the results obtained by these two approaches were numerically comparable. Thus, we have chosen to use the ELIspot assay as the principal approach to evaluate tumor antigen-reactive CTL responding to vaccines. A comparison of ELIspot data with tetramer analysis can provide valuable information regarding the effectiveness of an immunotherapeutic approach, as the total number of T cells capable of responding can be compared to the number of cells that are activated as a result of vaccination. We have found data from both approaches tend to be corroborative (59,59). We plan to use the ELIspot and tetramer assays as the primary measures for assessing T cell responses to class I MHC restricted peptides in the proposed study.

<u>Immune Monitoring Assays for $CD4^+$ T-cells</u>
In the proposed study, we plan to include a modified tetanus peptide (Table 3) (31), which binds to multiple HLA-DR alleles, and the 6-MHP (Table 2). We have found measures of proliferation, by incorporation of tritiated thymidine, are effective for detection of helper responses to the modified tetanus peptide, and this assay will be used in the current study to evaluate T_h responses to the class II MHC-restricted peptides. Characterization of the helper T cell response may aid in detection of differences in the immunologic milieu when the vaccines are administered following a single course of cyclophosphamide. This can be achieved by measuring cytokines secreted into the media by $CD4^+$ T cells proliferating in response to antigen, using an ELISA assay (31). The T_h1/T_h2 balance can be assessed by the IFNγ/IL-5 ratio.

<u>Safety of Peptide-based Vaccines</u>
Peptide-based vaccines have been administered safely to humans in a number of clinical trials, with toxicity limited usually to local injection site reactions and transient grade I-II systemic reactions. In our experience, other systemic toxicities are attributable to cytokines added as adjuvants, but the peptides themselves appear to be very well tolerated (Woodson, et al., manuscript in press).

In previous studies conducted by the UVA-HITC, mixtures of melanoma-derived class I MHC-restricted peptides or class II MHC-restricted melanoma peptides were administered to participants. This is the first study where we are proposing to administer the melanoma-derived class I and class II MHC-restricted peptides in combination. Given the low toxicity profile associated with the individual peptide mixtures, we expect administration of the combination of peptides will be well-tolerated. The toxicity of the treatment regimen, however, will be carefully monitored.

<u>Safety of Administration of Low-dose Cyclophosphamide</u>

Minimal clinical toxicities were noted following the administration of escalating doses of cyclophosphamide (50-700 mg/m^2) once every 3 weeks; mild nausea and vomiting were reported in one of five participants at the highest dose (61). Laboratory toxicities included a decline in the average nadir for B- and T-cell lymphocytes over a range of doses, beginning at a dose of 100 mg/m^2 cyclophosphamide for B lymphocytes and 200 mg/m^2 for CD8$^+$ T lymphocytes (61). A single dose of cyclophosphamide (300 mg/m^2) has been safely administered to humans in clinical trials with limited toxicity reported (32,33). Cyclophosphosphamide has also been combined with cell-based vaccines for melanoma (300 mg/m^2) and renal cell carcinoma (100-1000 mg/m^2). Toxicities were mostly limited to local injection site reactions, mild fatigue, fever, and chills after vaccine administration (32,36,62).

Based on prior reports, serious clinical and laboratory toxicities are not expected to occur following administration of a single dose (300 mg/m^2) of cyclophosphamide. Additionally, there is a large body of experience supporting a combination of a single dose of cyclophosphamide (300 mg/m^2) and cell-based vaccines (32,36). However, the interaction of a peptide-based vaccine and this dose of cyclophosphamide could conceivably lead to unexpected toxicities or could increase the frequency and grade of toxicities compared to either agent alone. Thus, toxicity will be carefully monitored throughout the study and stopping rules have been included in Section 9 of the protocol.

<u>Summary</u>
The clinical study proposed is a multicenter phase I/II study seeking preliminary evidence to estimate (1) whether the administration of 12 melanoma peptides comprised of class I MHC-restricted epitopes (12-MP) in conjunction with 6 melanoma-derived class II MHC-restricted helper peptides (6-MHP) is safe and (2) whether the administration of cyclophosphamide (Cytoxan ®) prior to administration of a peptide-based vaccine is safe and (3) the magnitude of immune responses against the 12-MP when administered in conjunction with a tetanus toxoid-derived helper peptide or 6-MHP with and without the addition of cyclophosphamide.

Secondary goals are to obtain preliminary data on (1) the response rate and persistence of immune responses against the 12-MP when administered in conjunction with a tetanus toxoid-derived helper peptide or 6-MHP with and without the addition of cyclophosphamide, (2) the magnitude of immune responses against the tetanus toxoid-derived helper peptide or 6-MHP with and without the addition of cyclophosphamide, (3) the response rate and persistence of immune responses against the tetanus toxoid-derived helper peptide or 6-MHP with and without the addition of cyclophosphamide, (4) DTH responses to the peptide components of the vaccine, and (5) disease-free survival.

1.2 Vaccine Design

1.2.1 <u>Antigen-Specific Immune Responses Induced by Peptide Vaccines</u>

We have been successful at detecting immunological responses against a four peptide mixture of synthetic peptides derived from tyrosinase and gp100. In one of our earlier phase II clinical trials (UVA-Mel31), we evaluated the efficacy of vaccination with synthetic melanoma peptides, either pulsed on DC, or administered with GM-CSF-in-adjuvant, plus administration of systemic IL-2, in

participants with advanced melanoma. Using the same immunological assays to analyze both vaccination approaches, we detected immunological responses in 80% of participants immunized with the synthetic peptides plus GM-CSF-in-adjuvant versus 13% of participants immunized with the DC-peptide-pulsed vaccine (17). This supports continued use of the antigen delivery system in which peptides are administered in an emulsion with GM-CSF and Montanide ISA-51 adjuvant.

We also completed a trial evaluating the role of low-dose IL-2 in the generation of immunological responses against the synthetic peptides in a clinical trial (UVA-Mel36). Both in the peripheral blood and in a lymph node draining the vaccine site (sentinel immunized node, SIN), this study failed to support the hypothesis that low-dose IL-2 administered daily at 3 million units/m^2/day during the 6-week vaccine regimen would increase the CTL responses to vaccination (63). Based on these results, peptide vaccine mixtures were administered in future vaccine studies in the absence of low-dose IL-2 therapy.

In one of our more recent studies, UVA-Mel39, we expanded the number of peptides in our vaccine mixture from four to twelve (Table 1), and the peptides included were derived from both MDP and CTA. The purpose of this study was to assess whether the immunogenicity of low-affinity peptides is compromised when low- and high-affinity peptides are administered as part of the same peptide mixture. The number of immunogenic epitopes derived from melanoma-associated antigens is growing; therefore, the idea of administering a multivalent vaccine at several sites becomes increasingly less feasible with increasing numbers of peptides. Preliminary data from this study, however, suggest the immune response to a single peptide is maintained even when the peptide is administered in a mixture with other MHC-associated peptides, compared to administration of each peptide separately. Thus, these data support the continued administration of complex peptide mixtures, such as the 12-MP, in future peptide-based vaccine regimens.

One of the goals of the HITC-run UVA-Mel43 study is to determine whether or not GM-CSF administered locally changes the immunogenicity of vaccination with the12-MP in an emulsion with Montanide as an adjuvant. Preliminary data from UVA-Mel43 indicate that immune responses can be achieved in participants without the inclusion of GM-CSF in the vaccine. Since the cost of including GM-CSF is prohibitory to the successful administration of this study, we have decided to remove GM-CSF as a vaccine component. The removal of GM-CSF from the study is not expected to adversely affect participant safety.

1.2.2 <u>Melanoma Peptides Restricted by MHC Class I Molecules and Incorporated in the 12-Melanoma Peptide Mixture</u>

Antigens representing the majority of melanoma-associated antigens identified thus far can be divided into two groups. The first group consists of melanocytic differentiation proteins (MDP); such antigens are only expressed on cells of the melanocytic lineage and include MART-1/MelanA, gp100/Pmel17, tyrosinase, TRP-1/gp75 and TRP-2 proteins. The second group consists of cancer-testis antigens (CTA) such as MAGE proteins, GAGE-1/2, BAGE, RAGE, and NY-

ESO-1. These proteins are encoded by genes expressed in several tumors of different histologic types, but not in normal tissues, other than testis and placenta. Peptides derived from these proteins are recognized by CTL in the context of various HLA alleles, including the HLA-A1, -A2 (A*0201), and -A3 alleles.
Gp100 is an enzyme involved in melanin synthesis. Multiple epitopes restricted by HLA-A2, HLA-A3, and HLA-A24 have been identified from this protein (64), and we chose to incorporate $gp100_{209-217}$ IMDQVPFSV (substitution of M for T at position 210; HLA-A2), $gp100_{280-288}$ YLEPGPVTA (HLA-A2), $gp100_{17-25}$ ALLAVGATK (HLA-A3), and $gp100_{614-622}$ LIYRRRLMK (HLA-A3) into the 12-MP.

The parent $gp100_{(209-217)}$ epitope (ITDQVPFSV) has been modified to incorporate a methionine residue at position 210 of the peptide (IMDQVPFSV). This modification increases the binding affinity of the IMDQVPFSV peptide for HLA-A2 and increases its immunogenicity *in vivo* when compared to the native peptide (65,66). A second gp100 derived epitope, YLEPGPVTA, is naturally processed and presented by melanoma cells, and recognized *in vitro* by melanoma-reactive human cytotoxic T cell lines (67). The YLEPGPVTA peptide has been used in phase I (UVA-Mel16) and phase II (UVA-Mel31, UVA-Mel36, UVA-Mel39, UVA-Mel43) studies conducted by the UVA-HITC. In the Phase I study, T cell responses to the immunizing peptide 946 (YLEPGPVTA; gp100 $_{280-288}$) were observed in 3 of 21 participants (14%), and a favorable survival rate of 74% has been observed at 4.5 years (31). No toxicity was attributable to that peptide at 100 mcg doses. In the Phase II studies evaluated thus far (UVA-Mel31 and UVA-Mel36), immune responses to this peptide were observed as well ((17), (63)). A third gp100 derived epitope, ALLAVGATK, is also naturally processed and presented by melanoma cells, and holds promise for induction of HLA-A3 restricted responses *in vivo* (68). Thus far, immunological responses to the ALLAVGATK peptide have been detected in the UVA-Mel31, 36, and 39 studies ((17), (63) and manuscript in preparation). The last gp100 peptide included in this study (LIYRRRLMK) was identified by Kawakami et al. (21), and had not been tested in humans until the UVA-Mel39 and UVA-Mel43 studies, in which immunogenicity (UVA-Mel39) and safety (UVA-Mel39, UVA-Mel43) have been demonstrated (manuscripts submitted and in preparation). T-cell lines cultured from lymphocytes infiltrating human melanomas, when administered adoptively to participants with metastatic melanoma, have induced partial or complete remissions in a large subset of participants. Clinical response to this therapy has been reported to be increased when the T-cells used for therapy recognize peptides derived from Pmel-17/gp100 (69). Thus, there is substantial evidence to support the use of the gp100-derived peptides in the 12-MP.

Tyrosinase is an enzyme that is involved with melanin synthesis. Thus far, epitopes derived from tyrosinase have been identified for HLA-A1, HLA-A2, HLA-A24, and HLA-B44 class I alleles (64). In the proposed study, we are including two epitopes (DAEKSDICTDEY, SSDYVIPIGTY) restricted by HLA-A1 and one epitope (YMDGTMSQV) restricted by HLA-A2. The parent epitope of $tyrosinase_{240-251,}$ DAEKCDICTDEY, has been modified to incorporate a serine residue at position 244 of the peptide (DAEKSDICTDEY). This modification

prevents disulfide bond formation within the peptide, but does not interfere with HLA-A1 binding or with T cell recognition (70). In phase II clinical trials conducted by the UVA-HITC (UVA-Mel31, UVA-Mel36, UVA-Mel39), immunological responses against the DAEKSDICTDEY peptide were detected in the majority of participants who expressed HLA-A1 and who were immunized with a synthetic peptide mixture containing the DAEKSDICTDEY peptide ((17), (63) and manuscript in preparation). CTL cultured from the vaccine draining node (sentinel immunized node, SIN) were capable of lysing HLA-A1^{+} tumor cells naturally expressing tyrosinase (59). The second HLA-A1 restricted epitope, tyrosinase$_{146-156}$ SSDYVIPIGTY, was identified by Kawakami et al. (21), and had not been tested in humans until our current studies (UVA-Mel39 and UVA-Mel43), in which safety has been demonstrated. The naturally occurring HLA-A2 restricted epitope from tyrosinase, tyrosinase$_{369-377}$ YMDGTMSQV, contains a post-translational modification of an asparagine to aspartic acid at residue 371 (71). In phase II clinical trials conducted by the UVA-HITC (UVA-Mel31, UVA-Mel36, UVA-Mel39) immunological responses to the YMDGTMSQV peptide have been detected in the majority of stage III and IV melanoma participants who expressed HLA-A2, and were immunized with a synthetic peptide mixture containing this peptide ((17) and manuscripts in preparation and in press).

MAGE-A1, MAGE-A3, and MAGE-A10 are members of the MAGE gene family and are expressed in the testes, as well as in several different tumor types such as melanoma, breast, prostate, esophagus, colon, and lung (64). Two epitopes derived from MAGE-A1 (EADPTGHSY, SLFRAVITK) are included in the proposed study. EADPTGHSY was identified by Traversari et al. (72) and was recently incorporated into several different clinical trials (15,19,73). In the studies conducted by Hu et al. (19) and Mukherji et al. (73), participants were vaccinated with EADPTGHSY-pulsed autologous antigen presenting cells. Autologous melanoma-reactive and peptide-specific CTL were detected *in situ* at the vaccination site and in distal tumor sites in HLA-A1 participants whose melanoma cells were positive for MAGE-A1 mRNA expression (19). Furthermore, peptide-specific CTL were also detected in the circulation (73). In the study conducted by Nestle et al. (15), six HLA-A1 expressing melanoma participants were immunized with autologous dendritic cells pulsed with a mixture of HLA-A1 peptides including MAGE-A1$_{161-169}$ EADPTGHSY and MAGE-A3 $_{168-176}$ EVDPIGHLY. Delayed-type hypersensitivity reactions were detected in five out of six participants, and a partial clinical response was detected in one out of six participants. However, immunological responses against the individual epitopes were not determined. The MAGE-A1$_{96-104}$ epitope (SLFRAVITK) was identified by Chaux et al. (20), and had not yet been tested in humans until UVA-Mel39, in which immunogenicity and safety have been demonstrated (manuscript in preparation).

The MAGE-A3$_{168-176}$ epitope (EVDPIGHLY) was identified by Gaugler et al. (74) and has recently been incorporated into a DC-based vaccine (4). In the study by Thurner et al. (4), participants with melanoma were vaccinated with autologous DC pulsed with the MAGE-A3$_{168-176}$ epitope. Expansions of epitope-specific CTL precursors were induced in 8 out of 11 participants and regressions of individual metastases (skin, lymph node, lung, and liver) were

detected in 6 out of 11 participants (4). Immune responses to MAGE-$A3_{168-176}$ have also been detected in the majority of participants vaccinated under the UVA-MEL39 study (manuscript in preparation).

The MAGE-$A10_{254-262}$ epitope (GLYDGMEHL) was identified by Huang et al., and was shown to be naturally processed and presented by HLA-$A2^+$/MAGE-$A10^+$ melanoma cell lines (23). In a recent report by Valmori et al., CTL responses to this epitope were readily detected in two-thirds of participants with melanoma whose tumors tested positive for the expression of MAGE-A10 (75). However, the ability of MAGE-A10 synthetic peptide to stimulate immunologic responses in humans had not been tested until UVA-Mel39, in which immunogenicity and safety have been demonstrated (manuscript in preparation).

NY-ESO-1, like MAGE, is expressed in the testes and in several tumor types including melanoma, ovary, bladder, breast, prostate, and lung (64). One epitope derived from NY-ESO-1 has been incorporated into the proposed study, NY-ESO-1_{53-62} (ASGPGGGAPR). This epitope was identified by Wang et al. (22), and was shown to be restricted by HLA-A31, a member of the A3 supertype family. Recent studies at UVA have indicated that in addition to HLA-A31, NY-ESO-1_{53-62} associates with other members of the A3 supertype family, specifically HLA-A3 (unpublished observation). This epitope had not been tested in humans until UVA-Mel39, in which immunogenicity and safety have been demonstrated (manuscript in preparation).

1.2.3 Tetanus Toxoid Helper Peptide

Several studies have provided evidence of the importance of including T-helper epitopes when vaccinating with purified epitopes for B-cells or for CTL (76). We have selected a characterized T-helper epitope from tetanus toxoid, to which the vast majority of the population has been sensitized, to induce a T-helper response in the microenvironment of the immunizing peptides (76). One helper peptide we intend to use in the proposed study is a slightly modified form of a 15 residue peptide p2 of tetanus toxoid (QYIKANSKFIGITEL), representing amino acid residues 830-844. This peptide binds to class II MHC molecules of all participants tested, and specifically to HLA-DR1, DRw15(2), DRw18(3), DR4Dw4, DRw11(5), DRw13(w6), DR7, DRw8, DR9, DRw52a, and DRw52b, which account for 80-90% of the population. Because glutamine at the N-terminus can cyclize, giving rise to a slight change in molecular structure, we have prepared this peptide as a 16-residue species, where an alanine residue has been added to the N-terminus. Thus, the sequence of the tetanus peptide used is AQYIKANSKFIGITEL. The ability of the tetanus peptide to induce T-helper responses is not abrogated by addition of one or several residues to the N-terminus (77).

In a Phase I trial (UVA-Mel16) and in a Phase II trial (UVA-Mel31), we have detected immunological responses against this modified version of the tetanus peptide following vaccination, in the majority of participants vaccinated with peptide in adjuvant. These responses are detectable as proliferative responses of $CD4^+$ T cells in the peripheral blood. In addition, the responding

T-cells secrete the T_h1 type cytokine IFNγ, but neither of the T_h2 type cytokines IL-4 or IL-10.

1.2.4 Melanoma Peptides Restricted by MHC Class II Molecules and Incorporated in the 12-Melanoma Peptide Vaccine

Several studies have provided evidence on the importance of including T-helper epitopes when vaccinating with purified epitopes for B-cells or for CTL (76). We have found a tetanus toxoid peptide induces a systemic T_h1-type T-helper response that is readily detectable in the majority of participants. However, the current protocol will introduce peptide epitopes for T_h cells derived from melanoma proteins, and their immunogenicity will be assessed in the periphery. We hypothesize that induction of melanoma-specific class II MHC-restricted responses will be more effective at augmenting class I MHC-restricted responses than T_h responses directed against non-melanoma derived epitopes.

Like the melanoma specific class I MHC-restricted peptides, those restricted by class II MHC are derived from MDPs and from CTAs. Tyrosinase is a source of two of the peptides. The first HLA-DR-restricted peptides recognized by T-cells on melanoma were tyrosinase $_{56\text{-}70}$ and tyrosinase $_{448\text{-}462}$ (78). Both peptides require high concentrations to induce T cell responses, but the former peptide has a higher binding affinity for HLA-DR4 than the latter; therefore, tyrosinase $_{56\text{-}70}$ (QNILLSNAPLGPQFP) is chosen for use in this study. A DR15-restricted peptide, tyrosinase $_{386\text{-}406}$ was reported also to be an antigen for T_h cells and was selected for use in this study (79).
We have collaborated with the laboratory of Dr. Walter Storkus at the University of Pittsburgh Cancer Institute (UPCI) on the identification of peptides derived from MDPs and presented by class II MHC molecules. Peptides presented by HLA-DR4 from MART-1/Melan-A have been identified and we have chosen to include MELAN-A/MART-$1_{51\text{-}73}$ in this trial. This epitope is currently being tested in a clinical trial conducted by Dr. John Kirkwood at UPCI. A third MDP is gp100, from which a peptide at residues 44-59 has been identified. T-cells sensitized against this peptide can recognize melanoma cells and this epitope has been demonstrated to be naturally processed and presented in the context of HLA-DR4 (80-82). This peptide also appears to be an epitope in the context of DR1, and recent data from the laboratories of Dr. Kirkwood at UPCI and of Dr. Slingluff at UVA demonstrate this peptide binds to HLA-DR1, -DR3, -DR4, -DR7, and -DR13 (Kierstead LS, Kirkwood J, Slingluff CL Jr, Storkus WJ, et al.: unpublished observations and manuscript submitted for publication). This epitope is currently being tested in a clinical trial conducted by Dr. Steven Rosenberg at the National Cancer Institute (NCI).

The CTA to be included are from MAGE proteins. The peptide MAGE-A3 $_{281\text{-}295}$ can stimulate peptide reactive CTL *in vitro* and is strongly recognized by DR11-restricted MAGE-3 reactive CTL (83). Another MAGE peptide is homologous with MAGE-1, 2, 3, and 6, and is restricted by DR13. It represents MAGE $_{121\text{-}134}$ (84).

1.2.5 Montanide ISA-51 as a Vaccine Adjuvant

Montanide adjuvant has been effective at inducing immune responses against murine viral antigens when administered with a synthetic peptide epitope (85,86). Recently, Montanide ISA-51 has also been used in a peptide plus GM-CSF-in-adjuvant vaccine useful for enhancing both cellular and humoral immunity (87). The product consists of a mineral oil base similar to incomplete Freund's adjuvant. However, the Arlacel A emulsifying agent of incomplete Freund's, which has caused reactions in the past, has been replaced with a purified manoside monooleate called "montanide", which appears to be safer. The UVA HITC has sponsored studies where peptide-based vaccines in Montanide ISA-51 have been safely administered to more than 200 participants. Immunological responses against the immunizing peptides have been detected in most participants.

1.2.6 Integration of Peptide-Based Vaccines with Interferon Therapy

High-dose interferon (IFN) therapy is approved by the FDA for use in patients with resected stage IIB or III melanoma treated in the adjuvant setting. Approval was based largely on survival improvement observed following a one-year vaccination regimen with IFN as described in ECOG 1684 (88). However, survival improvement is modest and data from a subsequent trial, ECOG 1690, failed to reproduce differences in survival (89). Recent data from a third trial, ECOG 1694, reveal a statistically significant, but still modest, survival improvement with IFN therapy compared to a ganglioside vaccine (90). Thus, any patient with resected stage IIB or III melanoma should be offered IFN therapy, which is routine practice at UVA.

However, the toxicity of high-dose IFN therapy is substantial. Approximately 25-30% of patients withdraw from therapy because of toxicity, and approximately 60% of patients require dose reductions during treatment (88). The experience at UVA is that most patients who are candidates for therapy refuse IFN because of concerns with toxicity, despite being informed IFN therapy is the only FDA approved adjuvant therapy for melanoma and that this therapy may provide a survival advantage.

We have developed a standardized short document detailing the data on efficacy and toxicity of high-dose interferon, and in the UVA-Mel39 and UVA-Mel43 trials, have revised our consent form such that patients who are candidates for IFN must review the IFN information document, answer a short series of questions documenting understanding of several key points of fact about IFN therapy, and give informed consent to enter the peptide vaccine trial only if they refuse IFN after carefully considering the facts presented. This document has been cleared through the FDA in accord with IND 9847. We propose to use the same rigorous effort to educate patients about IFN and to require documentation of their understanding before accepting them as candidates for the present study.

1.2.7 Toxicology

There is no reason to expect direct toxicity of the melanoma peptides; they are not directly cytotoxic *in vitro*. On the other hand, because some of these peptides are identical or similar to a portion of a normal protein, risks of autoimmunity in humans are important to evaluate. Unfortunately, there is no

murine system adequately modeling the human immune response to these peptides. The most meaningful evaluation of this peptide vaccine mixture is in patients with melanoma. We intend to focus on participants with resected stage IIB/C, III, or IV melanoma. These individuals face a high risk (> 50%) of premature death, and the anticipated risk of short-term or long-term toxicity of this vaccine preparation is minimal, while the vaccine may delay or decrease the risk of morbidity and mortality due to melanoma in these patients. The potential implications of autoimmunity against cells of melanocytic lineage are illustrated by reported cases of vitiligo occurring coincident with regressions of melanoma (91). Most of these are limited, often occurring in skin surrounding the regressing melanoma, but occasionally occurring systemically. While pathogenesis of this phenomenon can only be hypothesized, it is reasonable to consider this a worst-case scenario.

The loss of skin and hair pigment can be striking in these cases, but is not a cause of morbidity or mortality. Of greater potential concern is the theoretical risk of damage to the retinal pigment epithelium; however, visual loss has not been reported either as a complication of successful immunotherapy with p946-specific CTL or spontaneous vitiligo; the NCI has reported that patients treated with TIL specific for peptide 946 have had clinical responses, and no visual/ocular toxicity in those patients has been reported (69).

Depigmentation of the retinal pigment epithelium has been observed in a small number of patients vaccinated with dendritic cells pulsed with MDP-derived peptides; however, this change was asymptomatic and was not associated with loss of visual acuity (personal communication – Frank Haluska). A careful study of the retinal pigment epithelium using monobenzyl ether of hydroquinone to induce pigment cell destruction on a biochemical basis suggests the safety of pigment cell destruction and supports immunotherapy directed against MDP as a strategy for melanoma therapy (personal communication, JM Kirkwood).

Thus far, several participants receiving peptide-based vaccines in the Phase I, Phase I/II, Pilot Phase II, or Phase II studies at UVA were diagnosed with definite vitiligo. In all cases, the vitiligo was asymptomatic. No visual/ocular toxicity was reported for these participants.

<u>Toxicities Previously Reported for Participants Receiving the 12-MP vaccine administered in GM-CSF-in-Montanide ISA-51 adjuvant</u>
Thus far, toxicity data are available for 51 participants who have been vaccinated at least six times with the 12-MP vaccine administered in GM-CSF-in-Montanide ISA-51 adjuvant. Toxicities were graded using the NCI Common Toxicity Criteria v2.0 or the NCI Common Terminology Criteria for Adverse Events v3.0 and are listed regardless of attribution. Maximum grade 2-4 toxicities experienced by at least 10% of participants included the following:

Table 4

Toxicity	Grade 2	Grade 3
Fatigue (lethargy, malaise, asthenia)	22%	-
Injection site reaction	69%	25%

Myalgia	18%	-

The grading of injection site reactions will be updated from CTCAE v2.0 to CTCAE v3.0. CTCAE v2.0 did not have an ulceration Adverse Event Term, therefore patients with ulceration in conjunction with an injection site reaction were graded as experiencing a grade 3 injection site reaction (ulceration or necrosis that is severe; operative intervention indicated). In prior HITC studies, an injection site reaction with ulceration ≤ 2 cm in maximal diameter at the injection site was an expected adverse event in a subset of patients. However, an injection site reaction that included ulceration >2 cm in diameter, or an injection site reaction requiring debridement, narcotic analgesics for pain, or surgery was not an expected toxicity and was considered a dose-limiting toxicity (DLT).

In CTCAE v3.0, ulceration is a separate Adverse Event Term; therefore, ulceration and injection site reactions can be graded separately. In the proposed study, we do not expect grade 3 injection site reactions; ulceration and injection site reaction toxicities will be expected at grade 2.

Toxicities Previously Reported for Participants Receiving the 6-MHP Vaccine Administered in GM-CSF-in-Montanide ISA-51 adjuvant

Thus far, toxicity data are available for 7 participants who have been vaccinated at least once with the 6-MHP administered in GM-CSF-in-Montanide ISA-51 adjuvant. Toxicities were graded using the NCI Common Terminology Criteria for Adverse Events v3.0. Grade 2-4 toxicities experienced by at least 10% of participants (n=1) included the following:

Table 5

Toxicity	Grade 2	Grade 3
Lymphopenia	-	14%
Fatigue (lethargy, malaise, asthenia)	14%	-
Injection site reaction	86%	14%
Lymphatics	14%	-

1.2.8 Dosing

The proposed dose of cyclophosphamide (300 mg/m^2) is less than the recommended initial dose, 40 to 50 mg/kg given intravenously in divided doses over a period of 2 to 5 days, when used as a single chemotherapeutic agent in patients with no hematologic deficiencies. The dose of 300 mg/m^2 has been used previously in combination to enhance vaccine-specific immune responses with minimal toxicity reported (32,33).

Because the peptide vaccine is sequestered locally, and the immune response occurs primarily locally and in the draining lymph nodes, the dose of the vaccine does not need to be scaled up proportionately to the size (by weight or body surface area) of the recipient, as might be done for a drug whose effect is related to its distribution in body fluid. Because direct toxicity of the peptide is not expected, dose escalation is not as meaningful as it would be with a drug with a narrow therapeutic index.

1.2.9 Regulatory Issues

The peptides for this trial have been synthesized under GMP conditions by Multiple Peptide Systems (San Diego, California) and have been vialed under GMP conditions as lyophylized preparations by Clinalfa (Merck Biosciences AG, Laufelfingen, Switzerland). Lot release testing of the final vialed peptide has also been completed by Clinalfa in accord with FDA guidelines. Stability testing will be conducted by Clinalfa and Microbiology Research Associates, Inc (Acton, MA).

Cyclophosphamide (Cytoxan®) is commercially available from MeadJohnson Oncology Products (Princeton, NJ). Montanide ISA-51 is available from Seppic, Inc. (Fairfield, NJ). A master drug file for Montanide ISA-51 is filed with the FDA.

This study will be approved by the Institutional Review Board (IRB) of the University of Virginia and of all participating institutions. This protocol will be submitted to the FDA as a new IND application and will cross-reference IND 9847 for the 12-MP and the tetanus peptide and IND 10825 for the 6-MHP. Records of all study review and approval documents will be kept on file by the Principal Investigator at each participating institution, and are subject to FDA inspection during or after completion of the study. The Institutional Review Boards will receive notification of study closure within three months of study termination or completion.

2.0 STUDY OBJECTIVES

Safety:

(1) whether the administration of 12 melanoma peptides comprised of class I MHC-restricted epitopes (12-MP) in conjunction with 6 melanoma-derived class II MHC-restricted helper peptides (6-MHP) is safe

(2) whether the administration of cyclophosphamide prior to administration of a peptide-based vaccine is safe

Immunogenicity:

Primary

(1) the magnitude of immune responses against the 12-MP when administered in conjunction with a tetanus toxoid-derived helper peptide or 6-MHP with and without the addition of cyclophosphamide (Group C>Group A, Group D>Group B, Group D>Group C, Group B>Group A).

Secondary

(1) the response rate and persistence of immune responses against the 12-MP when administered in conjunction with a tetanus toxoid-derived helper peptide or 6-MHP with and without the addition of cyclophosphamide

(2) the magnitude of immune responses against the tetanus toxoid-derived helper peptide or 6-MHP with and without the addition of cyclophosphamide

(3) the response rate and persistence of immune responses against the tetanus toxoid-derived helper peptide or 6-MHP with and without the addition of cyclophosphamide

(4) DTH responses to the peptide components of the vaccine

(5) Disease-free survival

3.0 STUDY OUTLINE

3.1 Type of Study

This is an open-label, multicenter phase I/II study of a vaccine comprised of a mixture of 12-MP administered in Montanide ISA-51. Participants will be randomized to receive the 12-MP in combination with a tetanus toxoid-derived helper peptide with or without a single pre-treatment with cyclophosphamide or to receive the 12-MP in combination with a 6-MHP mixture with or without a single pre-treatment with cyclophosphamide. Participants will be stratified by HLA type and by participating institution.

4.0 PARTICIPANT SELECTION

All questions regarding eligibility should be directed to the Director of the Office of Collaborative Studies (OCS) at (434) 982-1008 between the hours of 8:30 am and 5 pm Monday through Friday.

4.1 Inclusion Criteria

4.1.1 Participants with stage IIB, IIC, III or IV melanoma that have no clinical or radiological evidence of disease (NED) after surgical resection or stereotactic radiosurgery. These participants may have had cutaneous or mucosal primary melanoma, or an unknown primary melanoma. Staging must be confirmed by cytological or histological examination. Staging of cutaneous melanoma will be based on the revised AJCC staging system (Appendix 2) (92).

4.1.2 Participants will be required to have radiological studies to rule out radiologically evident disease. Required studies include:

- Chest x-ray or chest CT scan,
- Abdominal and pelvic CT scan, and
- Head CT scan or MRI

PET/CT fusion scan may replace scans of the chest, abdomen, and pelvis.

4.1.3 Participants who have had brain metastases will be eligible if all of the following are true:

4.1.3.1 The total number of brain metastases ever ≤ 3

4.1.3.2 Each brain metastasis must have been completely removed by surgery or each unresected brain metastasis must have been treated with stereotactic radiosurgery.

4.1.3.3 There has been no evident growth of any brain metastasis since the most recent treatment

4.1.3.4 No brain metastasis is > 2 cm in diameter at the time of randomization

4.1.4 The most recent surgical resections or gamma-knife therapy for malignant melanoma must have been completed ≥ 1 week and ≤ 6 months prior to randomization.

4.1.5 The interferon education packet must be completed satisfactorily for those who are eligible for, but refuse, interferon therapy. Participants

who are not candidates for interferon for the following reasons do NOT need to complete an interferon education packet:

- Active ischemic heart disease or cerebrovascular disease
- Anginal syndrome requiring ongoing medications or history of myocardial infarction or arrhythmia disorder
- History of treatment for depression, active depression, or other psychiatric disorder
- Autoimmune disorders
- Hypersensitivity to interferon-alpha or any component associated with interferon therapy
- Debilitating medical conditions such as severe pulmonary disease or severe diabetes mellitus
- Thyroid abnormalities, where thyroid function cannot be maintained in the normal range without medication
- Resected stage IV melanoma
- Discontinued interferon therapy due to the occurrence of a major toxicity that has been documented by the treating physician
- Experienced tumor progression while on interferon or after completing interferon therapy
- Missed the standard of care enrollment window for interferon therapy initiation

4.1.6 All participants must have:
- 4.1.6.1 ECOG performance status of 0 or 1 (Appendix 3)
- 4.1.6.2 Ability and willingness to give informed consent

4.1.7 Laboratory parameters as follows:
- 4.1.7.1 HLA-A1, -A2, or -A3 (+)
- 4.1.7.2 HLA-DR1, -DR4, -DR11, -DR13, or -DR15 (+)
- 4.1.7.3 ANC > $1000/mm^3$
- 4.1.7.4 Platelets > $100{,}000/mm^3$
- 4.1.7.5 Hgb > 9 g/dL
- 4.1.7.6 HGBA1C < 7%
- 4.1.7.7 Hepatic:
  - 4.1.7.7.1 AST and ALT ≤ 2.5 x upper limits of normal (ULN)
  - 4.1.7.7.2 Bilirubin ≦ 2.5 x ULN
  - 4.1.7.7.3 Alkaline phosphatase ≦ 2.5 x ULN
- 4.1.7.8 Renal
  - 4.1.7.8.1 Creatinine ≦ 1.5 x ULN
- 4.1.7.9 Serology (within 6 months of study entry)
  - 4.1.7.9.1 HIV negative
  - 4.1.7.9.2 Hepatitis C negative
- 4.1.7.10 LDH up to 1.5 x ULN

4.1.8 Age 18 years or older at randomization

4.1.9 Participants must have at least two intact (undissected) axillary and/or inguinal lymph node basins

4.2 <u>Exclusion Criteria:</u>

4.2.1 Participants with ocular melanoma.

4.2.2 Participants who have received the following medications or treatments at any time within 4 weeks of randomization:

- Chemotherapy

- Interferon (e.g. Intron-A®)
- Radiation therapy (Stereotactic radiotherapy, such as gamma knife, can be used ≥ 1 week and ≤ 6 months prior to randomization)
- Allergy desensitization injections
- Corticosteroids, administered transdermally, parenterally or orally. Inhaled steroids (e.g.: Advair®, Flovent®, Azmacort®) are not permitted. Topical corticosteroids are acceptable.
- Growth factors (e.g. Procrit®, Aranesp®, Neulasta®)
- Interleukins (e.g. Proleukin®)
- Any investigational medication

4.2.3 Participants who are currently receiving nitrosoureas or who have received this therapy within the preceding 6 weeks

4.2.4 Participants with known or suspected allergies to any component of the vaccine.

4.2.5 Participants may not have been vaccinated previously with any of the synthetic peptides included in this protocol.

4.2.6 Participants who have received vaccinations containing agents other than the synthetic peptides included in this protocol and have recurred during or after administration of the vaccine will be eligible to enroll 12 weeks following their last vaccination.

4.2.7 Pregnancy. Female participants of childbearing potential must have a negative pregnancy test (urinary or serum beta-HCG) obtained within 2 weeks prior to randomization. Males and females must agree, in the consent form, to use effective birth control methods during the course of vaccination.

4.2.8 Female participants must not be breastfeeding

4.2.9 Participants in whom there is a medical contraindication or potential problem in complying with the requirements of the protocol in the opinion of the investigator.

4.2.10 Participants classified according to the New York Heart Association classification as having Class III or IV heart disease (Appendix 4).

4.2.11 Participants with uncontrolled diabetes, defined as having a HGBA1C ≥ 7%.

4.2.12 Participants must not have had prior autoimmune disorders requiring cytotoxic or immunosuppressive therapy, or autoimmune disorders with visceral involvement. Participants with an active autoimmune disorder requiring these therapies are also excluded. The following will not be exclusionary:

- The presence of laboratory evidence of autoimmune disease (e.g. positive ANA titer) without symptoms
- Clinical evidence of vitiligo
- Other forms of depigmenting illness
- Mild arthritis requiring NSAID medications

4.2.13 Participants who have another cancer diagnosis, except that the following diagnoses will be allowed:

4.2.13.1 squamous cell cancer of the skin without known metastasis
4.2.13.2 basal cell cancer of the skin without known metastasis
4.2.13.3 carcinoma in situ of the breast (DCIS or LCIS)
4.2.13.4 carcinoma in situ of the cervix

4.2.13.5 any cancer without distant metastasis that has been treated successfully, without evidence of recurrence or metastasis for over 5 years

4.2.14 Participants with known addiction to alcohol or drugs who are actively taking those agents, or participants with recent (within 1 year) or ongoing illicit IV drug use.

4.2.15 Body weight < 110 pounds (without clothes) at randomization, due to the amount and frequency with which blood will be drawn.

4.3 <u>Demographics of Participant Population</u>

4.3.1 Age: 18 years or older. Both male and female participants are eligible, and participants of all races and ethnic backgrounds are eligible.

4.4 <u>Registration, Randomization, and Management of Participants</u>

4.4.1 Participant Registration

All participants must sign the consent form prior to determination of eligibility for this study. Participants eligible for interferon therapy must also satisfactorily complete the interferon education packet. All participants who meet the inclusion/exclusion criteria may be randomized. Registration information, the eligibility checklist with supporting documentation, and the on-study case report form for participants enrolled at institutions other than UVA must be received by the OCS Director.

4.4.2 Randomization

Randomization will be discussed with participants during the process of informed consent. Informed consent will be documented prior to randomization. The randomization lists are generated by the study statisticians and are held by a member of the CC CTO not affiliated with the study. The study CRC is given arm assignment from the holder of the randomization only at the time of randomization. Investigators affiliated with this study or the HITC do not have access to the randomization lists at any time. Upon receipt, a confirmation of registration and randomization information will be forwarded to the enrolling site by the HITC. Participants should receive the first scheduled DTH testing within 2 weeks post-randomization.

4.4.3 Management of Participants

This study will be conducted on an outpatient basis, with participants scheduled to be evaluated on days 1, 8, 15, 22, 29, 36, 43, 50, and weeks 12, 26, 39, and 52 and at 13 and 24 months (or more often if needed for testing or medical reasons). Participants will be off treatment follow-up at 24 months, or when another therapy is initiated, whichever occurs first. Once off treatment follow-up, participants will be followed yearly for disease-free survival.

5.0 STUDY MEDICATION FORMULATION, DOSAGE, AND ADMINISTRATION

5.1 <u>Study Medication Formulation</u>

5.1.1 <u>Peptide Synthesis and Storage</u>

All peptides were synthesized directly from amino acids by Multiple Peptide Systems (San Diego, CA). Recombinant vectors in bacteria or viruses were not used. The synthetic peptides were purified by HPLC.

The identity of the synthetic peptides has been confirmed by verifying their mass and amino acid sequences by mass spectrometry. Details of the synthesis, certificates of analysis, and technical summaries are included in the Chemistry and Manufacturing section of the IND application.

Each bulk peptide was supplied to the HITC as lyophilized powder without excipients and stored at a temperature ≤ -70°C and protected from light.

5.1.2 Reconstitution and Vialing of Peptides

Lyophilized peptides were reconstituted, mixed, and vialed under GMP conditions by Clinalfa (Merck Biosciences AG, Laufelfingen, Switzerland). Lyophilized peptides were supplied to the HITC as individual use vials. Details of the vialing are included in the Chemistry and Manufacturing section of the IND application.

5.1.3 Storage of Vialed Peptides

The vials of lyophilized peptide are stored by the HITC at a temperature ≤ -70°C and protected from light. Once thawed, the vial(s) must be used for preparation of the vaccine within 24 hours.

5.1.4 Lot Testing

Each lot of peptide vaccine is evaluated as required by the FDA for identity, sterility, general safety, purity, and pyrogenicity. In addition, studies of stability will be performed. The details of these tests are outlined in Appendix 5.

5.1.5 Labeling

The labeling of vials will occur as specified below.

Each vial of lyophilized peptide is labeled with the following information:

Short name of the product
Product number
Proper name of the product
Name and address of the vialing facility
Lot number
Date of manufacture (the date of vialing the reconstituted peptides)
Serial number
Quantity of each peptide per vial
Vial contains no preservative, store at ≤ -70°C
"Caution: New Drug – Limited by US Federal law to investigational use"

5.1.6 Purchase and Storage of Cyclophosphamide

Cyclophosphamide (Cytoxan®) is manufactured by MeadJohnson Oncology Products (Bristol-Myers Squibb Company, Princeton, NJ) as a clinical grade reagent in a form approved for human use. Cyclophosphamide will be prepared by and purchased from the institutional hospital pharmacy.

5.2 Preparation, Dosage, and Storage of Study Drug

5.2.1 Dosage and Preparation of Cyclophosphamide (Groups B and D)

Cyclophosphamide (300 mg/m^2) will be administered in 250 ml of saline over 30-60 minutes. Cyclophosphamide will be prepared by the investigational pharmacist in the hospital pharmacy in accord with the manufacturer's instructions.

Body Surface Area (BSA) should be calculated using the following

formula (93) or with a conventional hand-held nomogram:

$$\text{BSA (m}^2\text{)} = \left(\frac{ht(cm) \times wt(kg)}{3600}\right)^{1/2}$$

**Note: Participants who are morbidly obese (≥ 100 lbs. over their ideal body weight) will have their dose of cyclophosphamide adjusted. The dose administered will be based on the following adjustment: (actual body weight + ideal body weight) / 2. The calculated dose of cyclophosphamide will be rounded to the nearest 5 mg.

Ideal Body Weight (IBW) should be calculated as follows:

IBW (kg, females): $45.5 + \left[2.3 \times \left(ht(in) - 60\right)\right]$

IBW (kg, males): $50 + \left[2.3 \times \left(ht(in) - 60\right)\right]$

5.2.2 Dosage and Preparation of Peptide Vaccine
Class I MHC-restricted melanoma peptides (12-MP vaccine; 100 mcg) and the tetanus peptide (Peptide-tet; 200 mcg) or class II MHC-restricted melanoma peptides (6-MHP; 200 mcg) in aqueous solution are mixed 1/1 with Montanide ISA-51 to form water-in-oil emulsions (Appendix 6).

5.2.3 Storage of Prepared Peptide Vaccines
The prepared peptide vaccines will be stored in a plastic syringe and delivered to the clinicians in a plastic bag. This bag with the syringe will be stored at room temperature until the vaccine is administered. Ideally, the vaccine should be administered 1-2 hours after mixing. If the vaccine is not administered within 4 hours after mixing, it should be discarded.

5.3 Administration of Peptide Vaccines

5.3.1 Designation of Vaccine Sites
Evidence suggests nodes proximal to a tumor site may be relatively immunosuppressed; therefore, the vaccination sites will be distant from the primary tumor. In general, participants will be vaccinated in an upper arm (primary vaccine site) and thigh (replicate vaccine site) with intact draining nodes. Vaccines will be administered at the designated site(s). Chronic inflammatory reactions are expected to occur in all patients at their vaccine sites. Induration may persist for months, but is not expected to require additional therapy. Sterile abscesses may occur in some patients. These are not a basis for discontinuation of the vaccines. However, if the inflammatory reactions are severe, subsequent vaccines may be administered in adjacent skin (< 2 cm from prior injection site) rather than in the same location(s), to minimize additional morbidity. In the event the vaccine cannot be administered within 2 cm of the designated site(s), vaccination site(s) will not be reassigned and no further vaccines will be given.

5.3.2 Regimen

Group A (12-MP + tet)

- Days 1, 8, 15, 29, 36, 43 and weeks 12, 26, 39, 52: 100 mcg each of the 12 peptides (Table 1), 200 mcg of the tetanus toxoid peptide (Table 3) emulsified in Montanide ISA-51 adjuvant. The vaccine will be divided and administered at two sites, the primary and replicate vaccination sites, which are located on two different extremities. The vaccine will be administered subcutaneously and intradermally at both vaccination sites.

Group B (Cy + 12-MP + tet)

- Day -4: 300 mg/m^2 of cyclophosphamide administered intravenously in 250 ml of saline over 30-60 minutes.
- Days 1, 8, 15, 29, 36, 43 and weeks 12, 26, 39, 52: 100 mcg each of the 12 peptides (Table 1), 200 mcg of the tetanus toxoid peptide (Table 3) emulsified in Montanide ISA-51 adjuvant. The vaccine will be divided and administered at two sites, the primary and replicate vaccination sites, which are located on two different extremities. The vaccine will be administered subcutaneously and intradermally at both vaccination sites.

Group C (12-MP + 6-MHP)

- Days 1, 8, 15, 29, 36, 43 and weeks 12, 26, 39, 52: 100 mcg each of the 12 peptides (Table 1), 200 mcg of each the 6 peptides (Table 2) emulsified in Montanide ISA-51 adjuvant. The vaccine will be divided and administered at two sites, the primary and replicate vaccination sites, which are located on two different extremities. The vaccine will be administered subcutaneously and intradermally at both vaccination sites.

Group D (Cy + 12-MP + 6MHP)

- Day -4: 300 mg/m^2 of cyclophosphamide administered once intravenously in 250 ml of saline over 30-60 minutes.
- Days 1, 8, 15, 29, 36, 43 and weeks 12, 26, 39, 52: 100 mcg each of the 12 peptides (Table 1), 200 mcg of each the 6 peptides (Table 2) emulsified in Montanide ISA-51 adjuvant. The vaccine will be divided and administered at two sites, the primary and replicate vaccination sites, which are located on two different extremities. The vaccine will be administered subcutaneously and intradermally at both vaccination sites.

5.3.3 Post-Vaccination Observation

All participants will be closely observed for adverse events for at least 20 minutes following each vaccination. Any time thereafter, participants should report any adverse events to the research coordinator or research clinician.

5.4 Modification/Discontinuation of Treatment

5.4.1 Dose Modifications

There will be no dose modifications of the vaccine components.

5.4.2 Discontinuation

Protocol treatment will be discontinued for any of the following reasons:

5.4.2.1 Any dose-limiting toxicity as defined in Section 7.4.

5.4.2.2 In circumstances where assessment of an AE is limited, such as by intercurrent illness, or when laboratory studies are required to assess for other causes of toxicity, the vaccine schedule may be interrupted for up to 7 days. Delay of one vaccine administration by up to 7 days will not be considered a protocol violation if due to an AE, regardless of attribution. If more than one vaccine is delayed by 7 days due to an AE, regardless of attribution, treatment must be discontinued.

5.4.2.3 Disease progression requiring other therapy (e.g. surgery under general anesthesia, radiation, chemotherapy, or steroid therapy). The appearance of small metastases or recurrent tumor deposits will not be a basis for discontinuing the vaccinations. Biopsy to determine the nature of new lesions, or minor surgical procedures to excise a new lesion, will not be a basis for discontinuing vaccinations.

5.4.2.4 Initiation of cytotoxic chemotherapy, radiation therapy, surgery for resection of disease, steroid therapy, or other immunosuppressive therapy.

5.4.2.5 Any other potential adverse reaction deemed sufficiently serious to warrant discontinuation of therapy by the Principal Investigator or one of the Associate Investigators.

5.4.2.6 Noncompliance with the requirements of the study.

5.4.2.7 Therapy may be discontinued at the participant's request.

5.4.2.8 Therapy may be discontinued at the discretion of an Investigator.

5.4.2.9 Pregnancy. Pregnant participants will continue to be followed for the duration of the pregnancy.

5.4.3 Elective Withdrawal

A participant who is enrolled but receives no study drug (including DTH testing) will be replaced. A participant who receives any amount of study drug will not be replaced, but will be counted as having experienced a DLT with regard to the evaluation of safety. Every attempt will be made to evaluate any data from these participants for endpoint assessment. If a participant withdraws and no evaluable data are available, the participant will be considered a failure for tumor-reactive immune response.

5.4.4 Delayed Visit for Reasons Other than Toxicity

A schedule for return visits should be established at the first visit. If a participant misses a treatment, the missed treatment will be administered as soon as possible, and treatment will be continued for an additional time period so that the subsequent vaccinations are given in the appropriate intervals. Participants who are vaccinated outside of the established schedule should return to the original schedule as soon as possible.

The table below defines what constitutes a delayed visit, whether the participant should continue to be treated, and whether a protocol

violation should be reported and recorded. The range of days is counted from the original scheduled date.

**Delayed Visit for Reasons other than Toxicity**			
**Treatment Period**	**Range of Days**	**Participant Treatment**	**Protocol Deviation**
DTH Testing – 1st set			
Day -8	0	DTH placed	No
Days -7, -6	0	DTH read	No
	+/- 1 day	DTH read	Yes
DTH Testing – 2nd set			
Day 85 (Week 12)	+/- 7 day	DTH placed	No
	+/- 8 days	DTH placed	Yes
Day 86, 87	0***	DTH read	No
	+/- 1 day***	DTH read	Yes
Chemotherapy			
Day -4	+/-1 day	Labs/Chemotherapy	No
*Vaccines 1-3**			
Days 1, 8, 15	± 2 days	Vaccine/Labs	No
	± 3 to 7 days	Vaccine/Labs	Yes
	± 8 or more days	Labs	Yes
Assessment			
Day 22	± 2 days	Labs	No
	± 3 to 7 days	Labs	Yes
	± 8 or more days	Labs	Yes
*Vaccines 4-6**			
Days 29, 36, 43	± 2 days	Vaccine/Labs	No
	± 3 to 7 days	Vaccine/Labs	Yes
	± 8 or more days	Labs	Yes
Assessment			
Day 50 (Week 7)	± 2 days	Labs	No
	± 3 to 7 days	Labs	Yes
	± 8 or more days	Labs	Yes
*Vaccines 7-10**			
Weeks 12, 26, 39, 52	± 7 days	Vaccine/Labs/Scans**	No
	± 8 to 14 days	Vaccine/Labs/Scans	Yes
	± 15 or more days	Labs/Scans	Yes
Follow-up			
Months 13, 24	± 14 days	Labs/Scans**	No
	± 15 or more days	Labs/Scans	Yes

* A participant will be taken off protocol treatment if more than one vaccination is delayed [vaccines 1-6: ± 3 to 7 days, vaccines 7-10: ± 8 to 14 days] during the treatment period.

** Chest x-ray or CT on weeks 12, 26, 39, 52 and months 13 and 24 should also be completed within these time limits.

*** DTH must be read 24 and 48 hours after being placed. If the DTH placement scheduled for Day 85 is adjusted by one day, the subsequent readings must also be adjusted by one day.

5.5 Concomitant Medications

Medications taken in the month prior to randomization should be recorded on the baseline case report form. This includes prescription medications, over-the-counter medications, injected medications, biological products, blood products, imported drugs, or street drugs. Participants should be maintained

on drugs that they were taking prior to entry unless a change in regimen is medically indicated.

5.5.1 Non-permitted medications or treatments

5.5.1.1 Chemotherapy
5.5.1.2 Interferon therapy (e.g. Intron-A®)
5.5.1.3 Radiation therapy
5.5.1.4 Nitrosoureas
5.5.1.5 Allergy desensitization injections
5.5.1.6 Corticosteroids, administered parenterally, orally, or by inhalation (e.g.: Advair®, Flovent®, Azmacort®)
5.5.1.7 Growth factors (e.g. Procrit®, Aranesp®, Neulasta®)
5.5.1.8 Interleukins (e.g. Proleukin®)
5.5.1.9 Other investigational medications
5.5.1.10 Street drugs

5.5.2 Permitted medications or treatments

5.5.2.1 Nonsteroidal anti-inflammatory agents
5.5.2.2 Anti-histamines (e.g. Claritin®, Allegra®)
5.5.2.3 Topical corticosteroids
5.5.2.4 Short-term therapy for acute conditions not specifically related to melanoma
5.5.2.5 Chronic medications except those listed in section 5.5.1
5.5.2.6 Influenza vaccines are permitted, but should be administered at least 2 weeks prior to or at least 2 weeks after a study vaccine.
5.5.2.7 To minimize infusion-related adverse events, participants may receive up to 24 mg of zofran as a routine oral or intravenous (infused over 15 minutes) pre-medication 30-60 minutes prior to the administration of cyclophosphamide. Investigators are not limited to the use of this medication to minimize infusion-related adverse events; other medications may be substituted or the listed dosage may be altered as necessary.

6.0 CLINICAL AND LABORATORY EVALUATION

6.1 Screening

The following studies should be completed within 6 weeks prior to randomization unless otherwise noted.

6.1.1 Class I and Class II HLA-typing (any time prior to randomization)
6.1.2 Review of pathology (any time prior to randomization)
6.1.3 CBC with differential, including automated lymphocyte count if available (0.3 ml)
6.1.4 Comprehensive chemistry panel to include sodium, potassium, creatinine, fasting glucose, calcium, total bilirubin, AST, ALT, and alkaline phosphatase (0.9 ml)
6.1.5 Lactate Dehydrogenase (LDH) (0.3 ml)
6.1.6 Urinalysis
6.1.7 β-HCG for women of childbearing potential (within two weeks prior to randomization)
6.1.8 HGBA1C
6.1.9 HIV and HCV screening (within 6 months prior to randomization)
6.1.10 Chest x-ray or chest CT, abdominal and pelvic CT, and head CT or

MRI. PET/CT fusion scan may replace scans of the chest, abdomen, and pelvis.

6.1.11 Complete history and physical examination

- Vital signs
- Weight
- Performance Status
- Neurological function
- Assessment of skin and nodal basins for evidence of disease recurrence or metastasis
- Assessment of skin for vitiligo
- Hair and eye color noted
- Standard ophthalmoscopic, visual acuity exam using a Snellen chart, and color vision exams
- Designation of vaccination sites

6.1.12 Pre-study blood collection for Immunologic Testing
These samples will be processed by the Tissue Procurement Facility (TPF) and stored by the HITC Immune Monitoring Laboratory (IML).

- 120 cc heparinized green top tubes for lymphocytes
- 20 cc red top tubes for serum

6.2 Treatment

6.2.1 Day -8:

6.2.1.1 Baseline history and physical examination prior to placement of delayed type hypersensitivity test (DTH). Note: history and physical examination scheduled for Day -8 is not required if one has been performed ≤ 7 days prior.

- Vital signs
- Weight
- Performance status
- Neurologic function
- Assessment of skin for vitiligo
- Standard ophthalmoscopic, visual acuity exam using a Snellen chart, and color vision exams
- Note hair and eye color
- Review of adverse events
- Distribute new toxicity diary

6.2.1.2 CBC with differential, including automated lymphocyte count if available (0.3 ml). Note: CBC with differential scheduled for Day -8 is not required if one has been performed ≤ 7 days prior.

6.2.1.3 Comprehensive chemistry panel to include sodium, potassium, creatinine, glucose, calcium, total bilirubin, AST, ALT, and alkaline phosphatase (0.9 ml). Note: comprehensive chemistry panel scheduled for Day -8 is not required if one has been performed ≤ 7 days prior.

6.2.1.4 LDH (0.3 ml). Note: LDH scheduled for Day -8 is not required if one has been performed ≤ 7 days prior.

6.2.1.5 Anti-nuclear antibody and rheumatoid factor (4 ml total). Note: anti-nuclear antibody and rheumatoid factor scheduled for Day -8 may be performed at any time ≤ 7 days prior.

6.2.1.6 Delayed type hypersensitivity test is placed

6.2.2 Days -7 and -6: Delayed type hypersensitivity test is read by a research clinician

6.2.3 Day -4 (Groups B and D):
The following evaluations will be performed on an outpatient basis. Blood for these evaluations should be obtained prior to the administration of cyclophosphamide.

6.2.3.1 Evaluation prior to administration of cyclophosphamide
- Vital signs
- Weight

6.2.3.2 CBC with differential, including automated lymphocyte count if available (0.3 ml)

6.2.3.3 Comprehensive chemistry panel to include sodium, potassium, creatinine, glucose, calcium, total bilirubin, AST, ALT, and alkaline phosphatase (0.9 ml)

6.2.3.4 Review of participant toxicity diary and distribute new toxicity diary

6.2.4 Days 1, 8, 15, 22, 29, 36, 43, 50 and weeks 12, 26, 39, 52
The following evaluations will be performed on an outpatient basis on each of the listed days unless otherwise noted. Blood for these evaluations should be obtained prior to the vaccine injection if a vaccine is scheduled to be administered.

6.2.4.1 Interval history and physical examination directed at new signs and symptoms prior to administration of vaccine
- Vital signs
- Weight
- Performance status
- Neurologic function
- Assessment of skin for vitiligo
- Standard ophthalmoscopic, visual acuity exam using a Snellen chart, and color vision exams
- Note hair and eye color
- Review of adverse events
- Review of participant toxicity diary and distribute new toxicity diary

6.2.4.2 CBC with differential, including automated lymphocyte count if available (0.3 ml) (days 1, 8, 15, 22, 29 and weeks 12, 52).

6.2.4.3 Comprehensive chemistry panel to include sodium, potassium, creatinine, glucose, calcium, total bilirubin, AST, ALT, and alkaline phosphatase (0.9 ml) (days 1, 29, weeks 12 and 52)

6.2.4.4 AST and ALT (weeks 26 and 39)

6.2.4.5 LDH (0.3 ml) (days 1, 29 and weeks 12, 26, 39, and 52)

6.2.4.6 Anti-nuclear antibody and rheumatoid factor (4 ml total) (days 29 and week 52)

6.2.4.7 Chest x-ray or chest CT (week 12, 26, 39, and 52)

6.2.4.8 Abdominal and pelvic CT, and head CT or MRI. PET/CT fusion scan may replace scans of the chest, abdomen, and pelvis (Week 52)

6.2.4.9 Delayed type hypersensitivity placed (day 85)

6.2.4.10 Delayed type hypersensitivity read by a research clinician (days 86, 87)

6.2.4.11 Blood collection for immunologic testing (days 1, 8, 15, 22, 36, 50 and weeks 26, 52)
Samples will be processed by TPF and stored by the HITC IML.
- 75 cc heparinized green top tubes for lymphocytes
- 20 cc red top tubes for serum

6.2.4.12 Tissue collection
If during the study, participants develop metastases or recurrences, these may be removed, and following receipt by pathology, may be evaluated by the HITC. Tissue samples may be screened for antigen expression or protein profiles using tests such as Western blots, immunohistochemistry, PCR, flow cytometry or gene chip analysis. Tumor escape mechanisms may also be evaluated. Specimens will be used in immunological assays to assess T cell function or antibody response. Assays generally used for this type of testing include, but are not limited to, ELIspot assays, ELISAs, chromium-release assays, proliferation assays and intracellular cytokine staining. Specimens may be used to study the immunologic aspects of the tumor microenvironment or as targets or controls in laboratory assays. Specimens may be used to establish cell lines for long-term studies.
This tissue may also be compared to lesions resected prior to enrollment, which will be requested from the pathology department of each institution as paraffin-embedded tissue samples, and these tissues may be banked for use in future studies. If participants are removed from the study or progress during or after follow-up, tissue may be collected for use as part of this study, as described above, or banked for use in future studies.

6.2.4.13 New abnormalities of AST, ALT, or LDH levels, or new nodules on chest CT or chest x-ray will warrant more complete staging with CT scans of the chest, abdomen and pelvis and with CT or MRI of the brain as designated.
Participants who did not have elevated (> ULN) levels of LDH, AST, and/or ALT at baseline and experience levels > ULN during the study will be scanned.
Participants who did have elevated (> ULN) levels of LDH, AST, and/or ALT at baseline will be scanned if AST and/or ALT reach levels > 2.5 ULN or LDH reach levels > 1.5 ULN.

6.3 <u>Follow-up</u>
The following studies should be completed at months 13 and 24 unless otherwise noted.

6.3.1 Interval history and physical examination directed at new signs and symptoms
- Vital signs
- Weight
- Performance status

- Neurologic function
- Assessment of skin for vitiligo
- Standard ophthalmoscopic, visual acuity exam using a Snellen chart, and color vision exams
- Note hair and eye color
- Review of adverse events
- Review of participant toxicity diary (month 13)

6.3.2 LDH (0.3 ml) (month 24)

6.3.3 AST and ALT (month 24)

6.3.4 Chest x-ray or chest CT (month 24)

6.3.5 Abdominal and pelvic CT, and head CT or MRI. PET/CT fusion scan may replace scans of the chest, abdomen, and pelvis (month 24)

6.3.6 Blood collection for immunologic testing (month 24)
These samples will be processed by TPF and stored by the HITC IML.

- 75 cc heparinized green top tubes for lymphocytes
- 20 cc red top tubes for serum

6.3.7 Tissue Collection
If during follow-up, participants develop metastases or recurrences, these may be removed, and following receipt by pathology, may be evaluated by the HITC. Tissue samples may be screened for antigen expression or protein profiles using tests such as Western blots, immunohistochemistry, PCR, flow cytometry or gene chip analysis. Tumor escape mechanisms may also be evaluated. Specimens will be used in immunological assays to assess T cell function or antibody response. Assays generally used for this type of testing include, but are not limited to, ELIspot assays, ELISAs, chromium-release assays, proliferation assays and intracellular cytokine staining. Specimens may be used to study the immunologic aspects of the tumor microenvironment or as targets or controls in laboratory assays. Specimens may be used to establish cell lines for long-term studies.
This tissue may also be compared to lesions resected prior to enrollment, which will be requested from the pathology department of each institution as paraffin-embedded tissue samples, and these tissues may be banked for use in future studies. If participants are removed from the study or progress during or after follow-up, tissue may be collected for use as part of this study, as described above, or banked for use in future studies.

6.3.8 New abnormalities of AST, ALT, or LDH levels, or new nodules on chest CT or chest x-ray will warrant more complete staging with CT scans of the chest, abdomen and pelvis and with CT or MRI of the brain as designated.
Participants who did not have elevated (> ULN) levels of LDH, AST, and/or ALT at baseline and experience levels above ULN during follow-up will be scanned.
Participants who did have elevated (> ULN) levels of LDH, AST, and/or ALT at baseline will be scanned if AST and/or ALT reach levels > 2.5 ULN or LDH reach levels > 1.5 ULN.

6.4 <u>Sample Submission of Blood and Tissue for Immunologic Testing</u>

6.4.1 <u>Sample Submission Schedule</u>
Refer to protocol x-page for precise sampling dates.

The shipping kits will be provided to each institution upon request. To obtain shipping kits, notify the UVA HITC by fax (434-982-3276). Please allow two weeks for delivery of the shipping kits. Please make note that overnight shipping is NOT possible for delivery of shipping kits.

6.4.2 Sample Preparation Guidelines

Specimens should be sent immediately after procurement by overnight express mail on Monday to Thursday only; call ahead with regard to holidays. The specimens must be marked for morning delivery.

For the pre-vaccination screening, please submit:

- 10 cc green top tubes (total 120 cc blood needed; usually 14-16 tubes)
- 10 cc red top tubes (total 20 cc blood needed; usually 2-3 tubes)

At each time point after screening, please submit:

- 10 cc green top tubes (total 75 cc blood needed; usually 10-11 tubes)
- 10 cc red top tubes (total 20 cc blood needed; usually 2-3 tubes)

Each tube must be clearly labeled, including:
Protocol # Mel 44
Participant's initials
UVA registration number
HITC Laboratory number
Institution name
Date and time drawn
**Any questions can be directed to the OCS Director at (434) 982-1008.

6.4.3 Shipping Procedures

Blood collected into appropriate tubes should be placed in the mailing containers provided by the UVA HITC and shipped to the laboratory. Pre-addressed shipping labels are provided with the shipping kits. Ship by overnight express mail Monday through Thursday. Please phone ahead with regard to holidays.

University of Virginia
Human Immune Therapy Center
400 Lane Road
MR4 Building, Room 3038
Charlottesville, VA 22908
Tel: (434) 924-2129

Institutions MUST notify the UVA HITC prior to shipping samples by faxing a notice with the tracking number to (434) 982-3276. This will allow the HITC to track packages if there are any problems in delivery. If you are unable to get through to the laboratory by fax, telephone the OCS Director at (434) 982-1008.

**** NOTE: FOR MORE DETAILS ON SAMPLE SUBMISSION, PLEASE REFER TO THE STUDY OPERATIONS MANUAL**

7.0 REGULATORY AND REPORTING REQUIREMENTS

7.1 Risks and Safety

7.1.1 Agent-Specific Expected Adverse Events List:
AEs that do not require expedited reporting are noted below. These AEs are required to be reported in routine study data submissions to the UVA CC CTO. Any AE not on this list will be considered an unexpected AE.

This list of expected AEs is based on the following:
(a) Aggregate data from prior melanoma vaccine trials (UVA-Mel39, UVA-Mel43) conducted by the UVA HITC in which participants received a peptide vaccine with GM-CSF in adjuvant. Toxicities are listed only if observed in ≥ 4% of treated participants.
(b) Toxicities associated with the administration of cyclophosphamide were listed based on those described in the Cytoxan® drug package insert and through conversations with clinicians familiar with the administration of cyclophosphamide.

Grade 2:

**CTCAE Category**	**Description**	**Attribution**
Blood/bone marrow	Hemoglobin	Cyclophosphamide
Cardiac General	Pericarditis	Cyclophosphamide
Cardiac General	Myocarditis	Cyclophosphamide
Constitutional Symptoms	Fever (in the absence of neutropenia)	Peptide Vaccine
Constitutional Symptoms	Rigors/Chills	Peptide Vaccine
Dermatology/Skin	Injection site reaction	Peptide Vaccine
Dermatology/Skin	Nail changes	Cyclophosphamide
Dermatology/Skin	Pruritus	Cyclophosphamide
Dermatology/Skin	Rash: erythema multiforme	Cyclophosphamide
Dermatology/Skin	Ulceration	Peptide Vaccine
Gastrointestinal	Anorexia	Cyclophosphamide/Peptide Vaccine
Gastrointestinal	Nausea	Cyclophosphamide/Peptide Vaccine
Gastrointestinal	Colitis	Cyclophosphamide
Gastrointestinal	Diarrhea	Cyclophosphamide
Gastrointestinal	Mucositis/stomatitis (clinical exam) – oral cavity	Cyclophosphamide
Gastrointestinal	Vomiting	Cyclophosphamide
Infection	Febrile neutropenia	Cyclophosphamide
Infection	Infection with unknown ANC – lung (pneumonia), peritoneal cavity, urinary tract, upper airway, skin (cellulites)	Cyclophosphamide
Infection	Infection documented clinically or microbiologically with Grade 3 or 4 neutrophils – lung (pneumonia), peritoneal cavity, urinary tract, upper airway, skin (cellulites)	Cyclophosphamide
Infection	Infection with normal ANC or Grade 1 or 2 neutrophils -- lung (pneumonia), peritoneal cavity, urinary tract, upper airway, skin	Cyclophosphamide

	(cellulites)	
Metabolic/laboratory	Bilirubin (hyperbilirubinemia)	Cyclophosphamide
Metabolic/laboratory	Creatinine	Peptide Vaccine
Metabolic/laboratory	Hyperglycemia	Peptide Vaccine
Ocular	Watery eye	Cyclophosphamide
Pain	Pain - Muscle pain	Peptide Vaccine
Pain	Pain - Joint pain	Peptide Vaccine
Pain	Pain - headache	Peptide Vaccine
Pain	Pain – abdomen NOS	Cyclophosphamide
Pain	Pain -- sinus	Cyclophosphamide
Pulmonary	Cough	Peptide Vaccine
Pulmonary	Dyspnea (shortness of breath)	Peptide Vaccine
Pulmonary	Pneumonitis/pulmonary infiltrates	Cyclophosphamide
Renal/Genitourinary	Cystitis	Cyclophosphamide
Sexual/Reproductive Function	Infertility/sterility	Cyclophosphamide
Syndromes	Flu-like syndrome	Peptide Vaccine

Grade 3:

CTCAE Category	Description	Attribution
Allergy/Immunology	Allergic Reaction/hypersensitivity reaction (including drug reaction)	Cyclophosphamide
Blood/bone marrow	Leukocytes (total WBC)	Cyclophosphamide
Blood/bone marrow	Lymphocytes	Cyclophosphamide
Blood/bone marrow	Neutrophils/granulocytes (ANC/AGC)	Cyclophosphamide
Blood/bone marrow	Platelets	Cyclophosphamide
Constitutional Symptoms	Fatigue (lethargy, malaise, asthenia)	Cyclophosphamide/Peptide Vaccine
Dermatology/Skin	Rash/desquamation	Cyclophosphamide

*NOTE: Hyperglycemia ≥ Grade 3 is not an expected event. If a non-fasting hyperglycemia adverse event occurs at a level ≥ Grade 3, a fasting serum glucose test will be ordered and the event will be re-graded appropriately. The fasting serum glucose measurement will be used for the purpose of determining reporting requirements.

7.1.2 Reporting of Subject Withdrawals/Dropouts Prior to Study Completion
Subjects who withdraw consent and those dropping out of the study secondary to an AE will be reported to the UVA IRB yearly on the IRB continuation form. A copy of this form will be forwarded to the CC Data and Safety Monitoring Committee (DSMC) for review.

7.2 <u>Adverse Event Reporting:</u>

7.2.1 Definitions

7.2.1.1 Adverse event (AE) – Any unfavorable and unintended sign (including an abnormal laboratory finding), symptom, or disease temporally associated with the use of a medical treatment or procedure regardless of whether it is considered related to the medical treatment or procedure (attribution of unrelated, unlikely, possible, probable, or definite). Medical conditions or diseases present before starting the investigational drug will be considered as treatment-related AEs if they worsen after starting study treatment.

7.2.1.2 Unexpected AE – Any adverse event not listed in Section 7.1.1

7.2.1.3 Serious AE – Any AE requiring 24 hour reporting according to section 7.2.2.3.

7.2.1.4 Attribution – The determination of whether an adverse event is related to a medical treatment or procedure. The attribution groups are:

Definite – Applies to those adverse events which, the Investigator feels are incontrovertibly related to the vaccine. An adverse event may be assigned an attribution of definitely related if or when (must have all of the following):

- It follows a reasonable temporal sequence from administration of the test drug.
- It could not be reasonably explained by the known characteristics of the subject's clinical state, environmental or toxic factors, or other modes of therapy administered to the subject.
- It disappears or decreases on cessation or reduction in dose with re-exposure to drug. (Note: this is not to be constructed as requiring re-exposure of the subject; however, the group of definitely related can only be used when a recurrence is observed.)
- It follows a known pattern of response to the test drug.

Probable – Applies to those adverse events for which, after careful consideration at the time they are evaluated, are felt with a high degree of certainty to be related to the test drug. An adverse event may be considered probably related if or when (must have three of the following):

- It follows a reasonable temporal sequence from administration of the test drug.
- It could not be reasonably explained by the known characteristics of the subject's clinical state, environmental or toxic factors, or other modes of therapy administered to the subject.
- It disappears or decreases on cessation or reduction in dose. There are important exceptions when an adverse event does not disappear upon discontinuation of the drug, yet drug-relatedness clearly exists (e.g. bone marrow depression, fixed drug eruptions, tardive dyskinesia).
- It follows a known pattern of response to the test drug.

Possible – Applies to those adverse events for which, after careful consideration at the time they are evaluated, a connection with the test drug administration appears unlikely but cannot be ruled out with certainty. An adverse event may be considered possibly related if or when (must have two of the following):

- It follows a reasonable temporal sequence from administration of the test drug.

- It could not readily have been produced by the subject's clinical stated, environmental or toxic factors, or other modes of therapy administered to the subject.
- It follows a known pattern of response to the test drug.

Unlikely – Applies to those adverse events for which, after careful consideration at the time they are evaluated, are judged to be unrelated to the test drug. An adverse event may be considered unlikely if or when (must have two of the following):

- It does not follow a reasonable temporal sequence from administration of the test drug.
- It could readily have been produced by the subject's clinical state, environmental or toxic factors, or other modes of therapy administered to the subject.
- It does not follow a known pattern of response to the test drug.
- It does not reappear or worsen when the drug is re-administered.

Unrelated – Applies to those adverse events, which after careful consideration, are clearly and incontrovertibly due to extraneous causes (disease, environment, etc.).

7.2.1.5 Adverse Event Classification -- The determination of under which adverse event classification an AE is reported. The adverse event classifications are:

Hematologic – Any AE coded under one of the following CTCAE v3.0 categories should be reported under the Hematologic adverse event classification:

- Blood/bone marrow
- Metabolic/laboratory

Non-hematologic – Any AE not reported under Hematologic, Ocular, Allergic, or Hospitalization should be reported under the Non-hematologic adverse event classification.

Ocular – Any AE coded under one of the following CTCAE v3.0 Adverse Event Terms should be reported under the Ocular adverse event classification:

1) A single experience of the following adverse events will be classified as a DLT:
   - Ocular/visual: Night blindness (nyctalopia)
   - Ocular/visual: Nystagmus
   - Ocular/visual: Ophthalmoplegia/diplopia (double vision)
   - Ocular/visual: Optic disc edema

- Ocular/visual: Retinopathy
- Ocular/visual: Scleral necrosis/melt

Participants will be referred for an ophthalmologic exam if any of these ocular adverse events occur.

2) A prolonged experience (e.g., lasting > 5 days) of the following non-severe adverse events will be classified as a DLT:
   - Ocular/visual: Vision – blurred vision
   - Ocular/visual: Vision – flashing lights/floaters

Participants will be referred for an ophthalmologic exam if any of these ocular adverse events occur.

3) Other AEs coded under Ocular/visual should be reported under the Non-hematologic adverse event classification (e.g. dry eye syndrome).

<u>Allergic</u> – Only AEs coded as Allergy/immunology: Allergic reaction/hypersensitivity (including drug fever) should be reported under the Allergic adverse event classification. Other AEs coded under Allergy/immunology should be reported under the Non-hematologic adverse event classification (e.g. autoimmune reaction).

<u>Hospitalization</u> – Hospitalization or prolongation of hospitalization associated with Grade 3, 4, or 5 events, unexpected and expected, and regardless of attribution. Hospitalization for expedited AE reporting purposes is defined as an inpatient hospital stay equal to or greater than 24 hours. Hospitalization is used as an indicator of the seriousness of the adverse event and should be reserved for situations where the adverse event truly fits this definition and not for hospitalizations associated with less serious events. For example, a hospital visit where a patient is admitted for observation or minor treatment (e.g., hydration) and released in less than 24 hours. Furthermore, hospitalization for pharmacokinetic sampling, is not an AE, and therefore is not to be reported either as a routine AE or in an expedited report.

7.2.2 Reporting of AEs

7.2.2.1 Reporting of AEs will begin when the participant is administered the study drug (e.g. DTH testing).

7.2.2.2 Events occurring in each subject will be reported to the HITC until 30 days post administration of the last vaccine regardless of attribution. Adverse events that are possibly, probably, or definitely related to the vaccine will be recorded until the participant completes treatment follow-up (month 24). If,

during treatment follow-up, the participant receives additional treatment, participants will be off treatment follow-up and will be followed yearly for disease progression and survival.

7.2.2.3 Reporting requirements

The process of reporting AEs will occur as follows:

A. Identify the type of event using the NCI CTCAE v3.0 (Appendix 7). The CTCAE provides descriptive terminology and a grading scale for each adverse event listed.
B. Grade the event using the NCI CTCAE v3.0.
C. Determine whether the AE is related to the vaccine. Attribution groups are as follows: unrelated, unlikely, possible, probable, and definite. Definitions for each attribution group can be found above.
D. Determine the prior experience of the AE. Expected events are those that have been previously identified as resulting from administration of the vaccine. An AE is expected when it is listed above.
E. Determine the adverse event classification of the AE. Adverse event classifications are as follows: non-hematologic, hematologic, ocular, allergic, and hospitalization. Definitions for each adverse event classification can be found above.
F. Review the tables below to determine if expedited reporting is required for the AE. Definitions for each adverse event classification can be found above.
G. AEs determined to require expedited reporting to the UVA HITC OCS must also be reported to the institution according to the local policy and procedures. The HITC will forward reported AEs to the FDA as described below.

Reporting to the UVA HITC OCS and the local IRB:
Use the tables below to determine when an AE should be reported.

24h	Report the AE by phone/fax to the HITC OCS within 24 hours, followed by a written report within 5 working days
5d	Submit a written report of the AE to the HITC OCS within 5 working days
	Report AEs on CRF bi-weekly to the HITC OCS

Attribution of Possible, Probable, Definite

Adverse event classifications	Grade 1		Grade2		Grade 3		Grade 4		Death*		Late Death	
	E	U	E	U	E	U	E	U	E	U	E	U
Hematologic				5d		24h	5d	24h	5d	24h	5d	24h
Non-Hematologic				5d		24h	5d	24h	5d	24h	5d	24h
Ocular		24h		24h		24h	5d	24h	5d	24h	5d	24h
Allergic				24h		24h	5d	24h	5d	24h	5d	24h
Hospitalization				5d	5d	24h	5d	24h	5d	24h	5d	24h

Attribution of Unrelated or Unlikely

Adverse event classifications	Grade 1		Grade2		Grade 3		Grade 4		Death*		Late Death	
	E	U	E	U	E	U	E	U	E	U	E	U
Hematologic							5d	5d	5d	5d		
Non-Hematologic							5d	5d	5d	5d		
Ocular							5d	5d	5d	5d		
Allergic							5d	5d	5d	5d		
Hospitalization					5d	5d	5d	5d	5d	5d		

*This includes all deaths within 30 days of the last dose of treatment with the investigational agent.
U = unexpected adverse event; E = expected adverse event
5d = 5 days; 24h = 24 hours

Contact Information:
UVA HITC OCS
Director
(434) 982-1008
Fax: (434) 982-3276
Box 801457, 1352 Jordan Hall
Charlottesville, VA 22908

Reporting to the CC CTO, UVA IRB, GCRC, and the FDA by the UVA HITC OCS:

The UVA HITC OCS, in conjunction with the CC CTO and the Principal Investigator (PI), is responsible for reporting AEs to the UVA IRB, GCRC, and FDA, per the following guidelines. If new findings affect the safety of participants enrolled in this trial, Investigators at collaborating institutions will be notified; safety reports should be provided to each Institutional Review Board according to the local policy and procedures.

3d — Report the AE by phone/fax to the CC CTO, UVA IRB, and FDA within 3 days, followed by a written report within 10 working days

10d — Submit a written report of the AE to the CC CTO, UVA IRB, and FDA within 10 working days

Attribution of Possible, Probable, or Definite

Adverse event classification	Grade 1		Grade2		Grade 3		Grade 4		Death*		Late Death	
	E	U	E	U	E	U	E	U	E	U	E	U
Hematologic				10d		3d	10d	3d	10d	3d	10d	3d
Non-Hematologic				10d		3d	10d	3d	10d	3d	10d	3d
Ocular		3d		3d		3d	10d	3d	10d	3d	10d	3d
Allergic				3d		3d	10d	3d	10d	3d	10d	3d
Hospitalization				10d	10d	3d	10d	3d	10d	3d	10d	3d

Attribution of Unrelated or Unlikely

Adverse event classification	Grade 1		Grade2		Grade 3		Grade 4		Death*		Late Death	
	E	U	E	U	E	U	E	U	E	U	E	U

Hematologic							10d	3d	10d	3d		
Non-Hematologic							10d	3d	10d	3d		
Ocular							10d	3d	10d	3d		
Allergic							10d	3d	10d	3d		
Hospitalization					10d	10d	10d	3d	10d	3d		

*This includes all deaths within 30 days of the last dose of treatment with the investigational agent.
U = unexpected adverse event; E = expected adverse event
10d = 10 days; 24h = 24 hours

7.3 Adverse Event Review and Monitoring

7.3.1 Adverse events will be recorded using paper AE forms, the UVA CC database, and participant toxicity diaries.

At each clinic visit, each participant will be completely evaluated by a licensed clinician. Routine disease-directed physical exam including performance status, blood collection, re-assessment for vitiligo, ophthalmologic exam, visual acuity testing, color vision exam, and examination of the skin and nodal basins for evidence of metastasis will be performed.

Participants will keep a daily diary of toxicities for days -8 through 80 and for 30 days after each booster vaccine is administered. The diaries will be reviewed by a research clinician prior to the next scheduled vaccine. During clinic visits, participants will also be asked about subjective symptoms including headache, malaise, fatigue, dyspnea, nausea, rash, diarrhea, abdominal discomfort, peripheral nerve pain, visual changes, appetite, tremors, night sweats, and ability to concentrate. Additional toxicities will be captured from laboratory tests. For each AE, date of onset, duration, grade, and attribution will be noted in the participant's study chart, will be recorded on CRFs, and will be entered into the CC CTO database.

After each vaccination, participants will be observed for AEs for at least 20 minutes. Follow-up phone calls will be made per the judgment of the research clinicians with regard to individual participant need. At least one member of the research team at the UVA HITC will be available at the following number 24 hours a day, 7 days a week to assist with participant questions and/or problems from other institutions: (434)-982-3500 (#1986).

In the event of an AE, appropriate action will be taken to ensure adequate care for the participant. If the participant is still on protocol, treatment delay or withdrawal from the protocol will be considered according to the protocol guidelines. If there are frequent major AEs on this protocol, the protocol will be closed based on the stopping guidelines included in section 7.4.

7.3.2 Individual AEs will be reviewed by the principal investigator, nurse practitioner(s), clinical research coordinator(s), and the Melanoma Team.

7.3.3 Adverse events are captured at each clinic visit. This data is reported to the CRC using CRFs and a toxicity diary. The CRC reviews these AEs

when data is entered into the CC CTO database. Data must be keyed into the CC CTO database within 30 days of reporting.

Serious AEs experienced by participants will be reviewed weekly during the Melanoma Team meeting. This weekly meeting will occur at least 40 times in a calendar year. Those present at the weekly meeting include the PI, sub-investigators, protocol development staff, biostatisticians, research nurses, research coordinators, laboratory specialists, and laboratory research managers. These meetings also include the review of individual participants to assess whether they are protocol candidates, whether AEs warrant discontinuation, and whether existing protocols should be continued or closed.

Each participating institution is expected to review AEs occurring at the individual institution on a bi-weekly basis. At least one representative from each institution will participate in a monthly teleconference to discuss AEs occurring at all institutions.

At multi-institutional study sites, participant data (CRFs, laboratory data, and participant diaries) will be submitted via FAX to the HITC OCS within 2 weeks from each participant visit. The HITC OCS will generate queries for late data at least quarterly. Sites will be given two weeks from the date of the query to remit late data. If late data are not submitted to meet this requirement, the HITC OCS will impose a halt in patient accrual <u>at the site in violation</u> until late data has been received and reviewed. Upon receipt, study personnel have 30 days in which to enter information from these documents into the CC CTO database.

7.3.4 The following laboratory values will be recorded in the CC CTO database, graded using the CTCAE v3.0 (if a grading category exists), and reported as described in Section 7.2.2.3:

1. Alk Phosphatase
2. ALT (GPT)
3. ANA
4. AST (GOT)
5. Basophil #
6. BIlirubin, total
7. Calcium
8. Creatinine
9. Eosinophil #
10. Glucose
11. Hepatitis C
12. β-HCG
13. HGB
14. HIV
15. HLA type
16. LDH
17. Lymph #
18. Monocyte #
19. Neutrophil #
20. PLT

21. Potassium
22. RF
23. Sodium
24. Urinalysis
25. WBC

Any abnormal laboratory values captured which are not included in the above list, but are gradeable according to CTCAE v3.0, will be recorded in the CC CTO database and reported as described in Section 7.2.2.3. The exception to this is the GFR lab value, which will not be captured even if abnormal since this is a calculated lab value of minimal clinical relevance.

7.4 Adverse Event Stopping Guidelines

Based on results from previous vaccinations with 12-MP and 6-MHP preparations, and published experience with cyclophosphamide at the dose planned, significant toxicity is not anticipated. The study will be monitored continuously for treatment-related adverse events within each vaccine preparation by the study PIs and by two formal interim analyses of safety data by arm for the independent CC DSMC. Data from the UVA-Mel39 vaccine trial (using 12-MP and tetanus peptide) indicate an 8% (2/25, 95% CI (1,23%)) rate of treatment-related unexpected grade 3 adverse events (dyspnea). For each arm, the treatment regimen will be considered safe as long as the number of participants experiencing an unexpected treatment-related adverse event (DLT) does not exceed 33%. Any arm for which the adverse event data indicate that more than 33% of patients accrued are known to have experienced a DLT will be closed to further accrual, except that 1 DLT observed out of the first 3 patients accrued to a study arm will be acceptable and will not result in the closing of that arm. The UVA CC DSMC will receive AE reports monthly stratified by study arm; review of DLTs will occur at this time.

Adverse events will be described and coded based upon the NCI CTCAE v3.0. A DLT is defined as any unexpected adverse event that is possibly, probably or definitely related and:

1. ≥ grade I ocular adverse events,
2. ≥ grade II allergic reactions,
3. ≥ grade III non-hematologic toxicities, and
4. ≥ grade III hematologic toxicities.

7.5 Responsibility

7.5.1 The University of Virginia Cancer Center Data and Safety Monitoring Committee (DSMC) will provide oversight of the conduct of this study. The CC DSMC will report to the UVA Protocol Review Committee (PRC).

In addition, each multi-institution site will be monitored to verify that all regulatory procedures have been followed, drug is accounted for, and the data is correct. The study monitor will either be an employee of the HITC or an authorized auditing agency. Each participant study binder including all CRFs and source documents will be reviewed after the week 7 and week 52 visit. Regulatory and pharmacy documentation will

be reviewed every 6 months. The monitor will work with each site to make sure all CRFs are completed appropriately, supporting documentation is available, clarifications are provided, and proper corrections are made.

7.5.2 The DSMC will review the following:

- All adverse events
- Audit results
- Application of study designed stopping/decision rules
- Whether the study accrual pattern warrants continuation/action
- Protocol violations
- Endpoint data

7.5.3 The CC DSMC will meet every month for aggregate review of data.

7.5.4 The CC DSMC will send reports to the PI, PRC and UVA IRB regarding the outcome of these reviews every six months and will notify the PRC, UVA IRB, and PI if immediate action is required. After receipt from the CC DSMC, the PI will forward all reports to the participating institutions by FAX. These reports will also be forwarded to the GCRC by the HITC upon receipt.

7.6 Endpoint Data

7.6.1 Endpoint data will be collected using HITC IML data forms, participant-specific binders, and the HITC laboratory database.

7.6.2 The HITC laboratory database, which has password-restricted access, is stored on the UVA Health System Computing Services secure server.

8.0 EVALUATION OF IMMUNOLOGIC RESULTS

The following laboratory criteria will be used to judge whether beneficial or detrimental effects have occurred. In general, immunological evaluations will be performed in batch.

8.1 ELIspot assay

Peripheral blood lymphocytes will be evaluated by ELIspot assay for the number of peptide-reactive T-cells.

8.2 Tetramer assay

Peripheral blood lymphocytes will be evaluated by flow cytometry after incubation with MHC-peptide tetramers for the number of peptide-reactive T-cells.

8.3 Enzyme-Linked Immunosorbent Assay (ELISA)

The quantity of IL-7 and IL-15 will be measured in serum pre- and post-chemotherapy. Cytokine profiles (IFNγ, TNFα, IL-2, IL-4, IL-5, IL-10) of $CD4^+$ T cells will be analyzed and the cells characterized as T_h1 or T_h2 type cells.

8.4 Proliferation assay

Responses to the class II MHC-restricted peptides will be evaluated using a proliferation assay.

8.5 Evaluation of tumor

Tumor tissue collected prior to enrollment or at the time of progression will be evaluated by routine histology and immunohistochemistry. In addition, *in vitro* evaluations of tumor tissue and tumor infiltrating lymphocytes may be completed.

9.0 STATISTICAL CONSIDERATIONS

This is a multicenter phase I/II study with randomization to one of four vaccine regimens in participants with resected melanoma. The primary goals are to determine safety of the treatment regimens and determine if magnitude of immune response can be increased by the inclusion of class II MHC-restricted helper peptides with and without the addition of cyclophosphamide. Secondary goals are to assess response rate, persistence of immune response, DTH response, and disease-free survival.

The study is designed to assess a partial ordering, specifically D greater than B or C, and B or C greater than A. The study is designed with interim safety assessments although sample size determination is based upon the difference of cumulative immune response measured in the PBL over the six vaccines compared to baseline counts. With this design there are four main comparisons of interest and the study will be designed to test these comparisons at the two-sided 2.5% significance level (10% overall) with 90% power at the alternative. Participants will be stratified by HLA-type and participating institution using the following categories:

HLA-type Stratification Categories:

1.) HLA - A_1^+
2.) HLA - A_2^+ / HLA - A_1^- / HLA – A_3^-
3.) HLA – A_3^+ / HLA - A_1^-

Participating Institution Stratification Categories:

1.) University of Virginia
2.) Non-UVA

Participant randomization will be based on a random assignment within strata with varying block sizes.

9.1 <u>Accrual</u>

Maximum accrual to the study is estimated to be 173 participants in order to accrue the target 160 eligible participants required to meet the study objectives. Maximum accrual is adjusted for a 5% ineligibility rate and, since this is a multicenter study, for the time delay between consent work-up and study enrollment. Once target accrual has been reached the later criteria allows for those within the screening process (signed consent but are having pre-study procedures completed to determine eligibility) to be enrolled. This allowance is not to exceed 5 participants. Based upon accrual rates to Mel 43, participant accrual is estimated at 98 per year (48 per year at UVA; 50 per year cumulative from the other institutions). Assuming UVa accrues approximately 86 of the 173 patients, accrual to this study should be completed in less than 2 years. All participants will be followed for a minimum of two years.

9.2 <u>Safety</u>

For each arm, the treatment regimen will be considered safe as long as the number of participants experiencing an unexpected treatment-related adverse event does not exceed the pre-specified safety boundary (> 33% of patients accrued to that arm experiencing a DLT). Any arm for which the adverse event data indicates that the safety boundary has been crossed will be closed to further accrual. The supporting data and specific guidelines for safety are given in Section 7.4.

9.3 Immunogenicity Endpoints

Immune response will be measured over several time points in the blood and will be based on results from the ELIspot assay. Several measures will be used to describe immune response. The maximum negative control will be subtracted from all counts. Negative counts are set to zero (control counts exceed number or responding T-cells).

a) Cumulative difference: the maximum over time of [sum over all the immunizing peptides of difference (post-pre)]
b) Cumulative ratio: the maximum (plus 1) over time of [sum over all the immunizing peptides of ratio (post/pre - 1)]
c) Maximum difference: maximum difference (post-pre) over all the immunizing peptides over all times
d) Maximum ratio: maximum ratio (post/pre) over all the immunizing peptides. A ratio less than one is set to one to indicate no response (fold response to baseline is less than one).
   Note: results from previous analyses indicate a square root and natural logarithm transformation is required to stabilize the variance estimates for measures of differences and ratios, respectively.

9.4 Sample Size Determination and Hypothesis Testing

Sample size determination is based on cumulative difference and cumulative ratio ("a" and "b" above) within a participant over the first six vaccines. Data from UVA-Mel39 with a 4 peptide and the 12 peptide vaccines both with GM-CSF, indicated a 60% and a 44% improvement in cumulative difference and cumulative ratio for the 4 peptide vaccine compared with the 12 peptide vaccine, respectively. Preliminary data from Mel 43 with the 12 peptide vaccine without GM-CSF indicates a mean cumulative difference (using a square root stabilizing transformation) of 47.6 with a standard deviation of 17.2 and a cumulative ratio (using a natural logarithm stabilizing transformation) of 3.8 with a standard deviation of 1.2.

For this study, evidence of at least a difference of approximately $1500/10^5$ counts for cumulative difference (30% increase) between any of the comparisons would be considered evidence of differing immune response. Assuming a two-sample t-test with a two-sided 2.5% type I error (10% over all comparisons) and 90% power would require 40 participants per arm. Assuming this minimum detectable difference among the four main comparisons, adjustment of the sample size for a 5% ineligibility rate and, since this is a multicenter study, for the time delay between consent work-up and study enrollment, this results in a target accrual goal of 173 participants (43 per arm).

Assuming arm A of the current study will result in a similar estimate of the cumulative ratio from the preliminary data on Mel 43 and using the estimated pooled standard deviation of 1.5, with 40 eligible participants per arm, we will be able to detect a 32% increase (to 5) with a two-sided 2.5% type I error t-test and 90% power.

Data from E1684 indicates a median disease-free survival of 1 year for

participants with resected melanoma (88). Assuming a minimum follow-up of 2 years with no censoring prior to the minimum follow-up and 40 participants per arm we will be able to estimate median disease-free survival within 16%.

9.5 Analyses

Primary analyses will be based on eligible participants. Primary assessment of immunologic endpoints will be based upon responses in the blood to the first six vaccinations. For hypothesis testing, participants who discontinue protocol therapy prior to collection of all blood samples for severe allergic reactions or adverse events, disease progression, or noncompliance will be considered an immune response failure if no response is observed in the evaluable samples (even if the number of evaluable samples is zero).

Appropriate (usually natural logarithm and square root) transformation of the data to stabilize the variance estimates will be employed. A likelihood ratio test will be used to test the two partial orderings of primary interest (94). For the 1st ordering, the null hypothesis that the means satisfy the order restriction is H_o: $\mu_A \leq \mu_B \leq \mu_D$ versus the alternative that places no restrictions on the means is H_a: that at least one pair is different. For the 2nd ordering, the null hypothesis is H_o: $\mu_A \leq \mu_C \leq \mu_D$ versus the alternative H_a: that at least one pair is different. For primary endpoints, if either null is rejected then two sample t-tests will be used to compare arms for the four main comparisons of interest. Response within allele type also will be summarized. Point estimates and 95% confidence intervals will be calculated for all summary parameters. For participants who display an immune response, repeated measure models will be used to assess differences in immune response patterns over time. Measurements in the blood taken after vaccines administered at weeks 26 and 52 will be used to assess the persistence of response. In responding participants, repeated measures models will be used to determine if response patterns decline or remain stable during the booster phase.

The study was powered for ordering of the groups with regard to immunologic outcome. The anticipated orderings are D > B > A or D > C > A. This takes into account the hypothesized advantages (for CD8 T cell responses) of adding melanoma helper peptides and cyclophosphamide, individually, or together. However, the outcome may well differ from what is predicted. At final analysis, it will be possible to order the four groups for disease-free survival. If the ordering for immunologic outcomes and for disease-free survival is the same, then this will provide preliminary data to support that treatment-related immunologic effects may have an impact or an association with treatment-related clinical effects. If the two orderings differ, this may reflect minor differences that are not of statistically significant impact, or may reflect disparity between biologic effect and clinical outcome. All subjects will be followed for disease progression and disease-free survival will be measured from study entry to disease progression or death from any cause. The method of Feltz and Dykstra (1985) will be used to assess the specified orderings among the arms and disease-free survival (95). In addition, Cox's proportional hazard models with immune response as a time dependent covariate will be used to describe the association between immune response and disease-free survival.

Other methods such as those of Song, Davidian and Tsiatis (2002) will be used to assess the effect of vaccination on the joint distribution of disease-free survival and immune response (96).

APPENDICES

Appendix 1: Clinical trial flow diagram and X-page

Appendix 2: AJCC Staging System

Appendix 3: ECOG Performance Status

Appendix 4: New York Heart Association Disease Classification

Appendix 5: Lot Testing

Appendix 6: Vaccine Preparation

Appendix 7: NCI Common Terminology Criteria for Adverse Events v3.0

Appendix 8: Immunologic Studies

Appendix 1: Clinical trial flow diagram and X-page

1) Obtain informed consent

2) Participant qualification, baseline evaluation, and screening

   a) History
   b) Physical exam
   c) Blood draws

3) Register with Clinical Trials Office

4) Day -8:
   a) Interval history
   b) Physical exam
   c) Blood draw
   d) Delayed type hypersensitivity placed

5) Days -7, -6: Delayed type hypersensitivity read

6) Day -4 (Groups B and D):

   a) Interval history
   b) Physical exam (vital signs and weight only)
   c) Blood draws
   d) Administration of cyclophosphamide

7) Days 1, 8, 15, 22, 29, 36, 43, 50 and weeks 12, 26, 39, 52

   a) Interval history
   b) Physical exam
   c) Blood draws (days 1, 8, 15, 22, 29, 36, 50 and weeks 12, 26, 39 and 52)
   d) Vaccination (days 1, 8, 15, 29, 36, 43 and weeks 12, 26, 39, 52)
   e) Delayed type hypersensitivity placed (day 85)
   f) Delayed type hypersensitivity read (days 86, 87)

8) Post-treatment follow-up (months 13 and 24)

   a) Interval history
   b) Physical exam
   c) Blood draws (month 24)

Studies & Tests	Pre	Active Treatment														Follow-up	
	Day	-8	-4	1	8	15	22	29	36	43	50	85	183	274	365	Months	
	Week	-2	-1	0	1	2	3	4	5	6	7	12	26	39	52	13	24
Informed consent *	X																
Class I & Class II HLA-typing *	X																
Pathology review *	X																
CBC with differential †	X	X#	X@	X	X	X	X	X				X			X		
Comprehensive chemistry †	X***, #	X#	X@	X				X				X			X		
LDH †	X#	X#		X				X				X	X	X	X		X
HGBA1C †	X																
ALT and AST													X	X			X
Urinalysis †	X																
β-HCG ‡	X																
HIV / Hepatitis C !	X																
Chest x-ray / CT †, ^	X											X	X	X	X		X
Abdominal CT †, ^	X														X		X
Pelvic CT †, ^	X														X		X
Head MRI / CT †, ^	X														X		X
History & physical †	X#	X#	X@	X	X	X	X	X	X	X	X	X	X	X	X	X	X
120cc green top tubes †	X																
75cc green top tubes				X	X	X	X		X		X		X		X		X
20cc red top tubes †	X			X	X	X	X		X		X		X		X		X
Anti-nuclear antibody / Rh factor		X%						X							X		
Cyclophosphamide (Groups B & D)			X@														
Vaccination				X	X	X		X	X	X		X	X	X	X		
DTH placed		X**										X**					
Participant diary distributed		X	X	X	X	X	X	X	X	X	X	X	X	X	X		

* Any point prior to randomization
† Pre-study within 6 weeks of randomization
‡ Within 2 weeks of randomization (for childbearing women)
! Within 6 months of randomization
** DTH placed on day -8 is read by a research clinician on days -7 and -6. DTH placed on week 12 (day 85) is read by a research clinician on days 86 and 87
*** To include fasting glucose
@Groups B and D only; only vital signs and weight required for history and physical
^ New abnormalities of AST, ALT, or LDH levels, or new nodules on CXR will warrant more complete staging with CT scans of the chest, abdomen and pelvis and with CT or MRI of the brain as appropriate.
# History & physical, comprehensive chemistry, LDH, and CBC with differential scheduled for Day -8 are not required if same tests were performed ≤ 7 days prior
% Anti-nuclear antibody / Rh factor scheduled for Day -8 may be performed at any time ≤ 7 days prior

Appendix 2: AJCC Staging System

Melanoma TNM Classification

T Classification	Thickness	Ulceration Status
T1	≤ 1.0 mm	a: without ulceration and level II/III
		b: with ulceration or level IV/V
T2	1.01 – 2.0 mm	a: without ulceration
		b: with ulceration
T3	2.01 – 4.0 mm	a: without ulceration
		b: with ulceration
T4	> 4.0 mm	a: without ulceration
		b: with ulceration

N Classification	# of Metastatic Nodes	Nodal Metastatic Mass
N1	1 node	a: micrometastasis*
		b: macrometastasis†
N2	2 – 3 nodes	a: micrometastasis*
		b: macrometastasis†
		c: in transit met(s)/satellite(s) without metastatic nodes
N3	4 or more metastatic nodes, or matted nodes, or in transit met(s)/satellites(s) with metastatic node(s)	

M Classification	Site	Serum Lactate Dehydrogenase
M1a	Distant skin, subcutaneous or nodal mets	Normal
M1b	Lung metastases	Normal
M1c	All other visceral metastases	Normal
	Any distant metastatsis	Elevated

* Micrometastases are diagnosed after sentinel or elective lymphadenectomy.

† Macrometastases are defined as clinically detectable nodal metastases confirmed by therapeutic lymphadenectomy or when nodal metastasis exhibits gross extracapsular extension.

Stage Groupings for Cutaneous Melanoma

	Clinical Staging			Pathologic Staging		
	**T**	**N**	**M**	**T**	**N**	**M**
**0**	Tis	N0	M0	Tis	N0	M0
**IA**	T1a	N0	M0	T1a	N0	M0
**IB**	T1b<br>T2a	N0<br>N0	M0<br>M0	T1b<br>T2a	N0<br>N0	M0<br>M0
**IIA**	T2b<br>T3a	N0<br>N0	M0<br>M0	T2b<br>T3a	N0<br>N0	M0<br>M0
**IIB**	T3b<br>T4a	N0<br>N0	M0<br>M0	T3b<br>T4a	N0<br>N0	M0<br>M0
**IIC**	T4b	N0	M0	T4b	N0	M0
**III‡**	Any T	N1<br>N2<br>N3	M0			
**IIIA**				T1-4a<br>T1-4a	N1a<br>N2a	M0<br>M0
**IIIB**				T1-4b<br>T1-4b<br>T1-4a<br>T1-4a<br>T1-4a/b	N1a<br>N2a<br>N1b<br>N2b<br>N2c	M0<br>M0<br>M0<br>M0<br>M0
**IIIC**				T1-4b<br>T1-4b<br>Any T	N1b<br>N2b<br>N3	M0<br>M0<br>M0
**IV**	Any T	Any N	Any M1	Any T	Any N	Any M1

* Clinical staging includes microstaging of the primary melanoma and clinical/radiologic evaluation for metastases. By convention, it should be used after complete excision of the primary melanoma with clinical assessment for regional and distant metastases.

† Pathologic staging includes microstaging of the primary melanoma and pathologic information about the regional lymph nodes after partial or complete lymphadenectomy. Pathology stage 0 or stage 1A patients are the exception; they do not require pathologic evaluation of their lymph nodes.

‡ There are no stage III subgroups for clinical staging.

Staging for Mucosal Melanomas
This system is based on the staging of cutaneous melanomas.

Stage IIB: Clinically localized primary melanoma > 4mm thick
Stage III: Lymph node metastases
Stage IV: Distant metastases

Appendix 3: ECOG Performance Status

Grade	Performance
0	Fully active, able to carry on all pre-disease performance without restriction
1	Restricted in physically strenuous activity but ambulatory and able to carry out work of a light or sedentary nature, e.g. light house work, office work
2	Ambulatory and capable of all selfcare, but unable to carry out any work activities. Up and about more than 50% of waking hours
3	Capable of only limited selfcare, confined to bed or chair more than 50% of waking hours
4	Completely disabled. Cannot carry on any selfcare. Totally confined to bed or chair
5	Dead

* As published in Am. J. Clin. Oncol.: Oken, M.M., Creech, R.H., Tormey, D.C., Horton, J., Davis, T.E., McFadden, E.T., Carbone, P.P.: Toxicity And Response Criteria Of The Eastern Cooperative Oncology Group. Am J Clin Oncol 5:649-655, 1982.

Appendix 4: New York Heart Association Disease Classification

Functional Capacity	Objective Assessment
**Class I.** Patients with cardiac disease but without resulting limitation of physical activity. Ordinary physical activity does not cause undue fatigue, palpitation, dyspnea, or anginal pain.	No objective evidence of cardiovascular disease.
**Class II.** Patients with cardiac disease resulting in slight limitation of physical activity. They are comfortable at rest. Ordinary physical activity results in fatigue, palpitation, dyspnea, or anginal pain.	Objective evidence of minimal cardiovascular disease
**Class III.** Patients with cardiac disease resulting in marked limitation of physical activity. They are comfortable at rest. Less than ordinary activity causes fatigue, palpitation, dyspnea, or anginal pain.	Objective evidence of moderately severe cardiovascular disease.
**Class IV.** Patients with cardiac disease resulting in inability to carry on any physical activity without discomfort. Symptoms of heart failure or the anginal syndrome may be present even at rest. If any physical activity is undertaken, discomfort is increased.	Objective evidence of severe cardiovascular disease.

* The Criteria Committee of the New York Heart Association. Nomenclature and Criteria for Diagnosis of Diseases of the Heart and Great Vessels. 9th ed. Boston, Mass: Little, Brown & Co; 1994:253-256

Appendix 5: Lot Testing

A. Preparation of the synthetic melanoma and tetanus peptides

All peptides were synthesized under GMP conditions by Multiple Peptide Systems (San Diego, CA). Certificates of analysis and technical summaries for each of the peptides are included in the chemistry and manufacturing portion of the IND application.

Peptide preparation and vialing will be performed under GMP conditions by Clinalfa (Merck Biosciences AG, Laufelfingen, Switzerland). Documentation relating to the procedures used to prepare and vial the peptides will be included in the Chemistry and Manufacturing Section of the IND application.

B. Quality Assurance Testing

Prepared peptides will be subjected to the following tests:

1. Identity. Identity will be confirmed by structural studies. The individual peptides are tested for identity by mass spectrometry (to define molecular mass and amino acid sequence) and HPLC (to confirm purity) in a GMP laboratory (Multiple Peptide Systems).

   Before mixing, the amino acid sequence of each individual peptide preparation is reconfirmed by mass spectrometry or co-elution in a GMP laboratory (Clinalfa).

   After combining the peptides, the mixture will be subjected to HPLC in a GMP laboratory (Clinalfa).

2. Purity. Purity is assessed before and after vialing the peptide mixtures. Before vialing the peptide mixtures, each synthetic peptide is evaluated for the presence of a single dominant species by high pressure liquid chromatography (HPLC) in a GMP laboratory (Multiple Peptide Systems). Purity of each peptide component exceeds 90% (94%-98%). Variants of the original peptide may include incomplete products of synthesis, minor degradation products due to oxidation of methionine residues, and dimerization of cysteine-containing peptides. After vialing the peptide mixture, purity will be reconfirmed by HPLC in a GMP laboratory (Clinalfa).
3. Trifluoroacetic acid (TFA). The amount of total fluorine in each peptide preparation will be less than 0.5% or 5000 ppm as determined by Multiple Peptide Systems.
4. Potency. Peptides are synthesized under GMP conditions and the net peptide content calculated for each. The amounts of each peptide (mcg quantities) added to the vaccine vials are calculated based on the net peptide content of the original stock of lyophilized peptides.
5. Pyrogenicity. Pyrogenicity testing will be conducted by Clinalfa in accordance with USP guidelines.
6. General Safety. General safety testing will be conducted by Clinalfa in accordance with USP guidelines.
7. Sterility. Sterility testing will be conducted by Clinalfa in accordance with USP guidelines.

8. Stability. The peptide preparations will be assayed for stability at months 3, 6, 12, 24, and 36. The following analyses will be performed to confirm stability.
   a. HPLC: HPLC will be performed to confirm purity. An optical comparison to previous HPLC data will be performed. Ideally, the purity of each peptide component will exceed 90% (94%-98%). Variants of the original peptide may include incomplete products of synthesis, minor degradation products due to oxidation of methionine residues, and dimerization of cysteine-containing peptides. Such minor variants will be tolerated as long as the peptide represents at least 75% of the intended peptide species. With liquid formulations of this peptide preparation, we have observed dimerization of the cysteine-containing peptide, yet this peptide retained immunogenicity even when as little as 10% remains as monomer. For the present study, dimerization of the DAEKSDICTDEY peptide will be acceptable as long as at least 25% remains as monomer by HPLC. Because measures of peptide quantity are subject to variability, a peptide lot will be rejected only if two sequential measures fail to meet the criterion stated above. If a second measure is required, the lot will be held until the second reading is obtained and shown to be acceptable.
   b. Sterility. One vial of peptide will be submitted to the Clinical Microbiology Laboratory at the University of Virginia or Microbiology Research Associates, Inc. (Acton, MA). Sterility testing will be conducted in accord with FDA guidelines in 21 CFR part 610.12.

Appendix 6: Vaccine Preparation

Note: All vaccines are to be made in a certified, sterile laminar flow hood.

Group A: 12 MP + Tetanus Peptide Vaccine

For vaccinations on days 1, 8, 15, 29, 36, and 43 and weeks 12, 26, 39, and 52:
3 ml of emulsion will be prepared. 2 ml of this emulsion will be administered subcutaneously and intradermally, at two vaccination sites. The remaining 1 ml will be discarded.

2 ml contains 100 mcg each of the 12 peptides listed in Table 1 and 200 mcg of tetanus peptide emulsified in Montanide ISA-51 adjuvant.

Materials
Gloves (sterile)
2-3 sterile gauze packets
70% ethanol
1 – 12 MP – “12 Melanoma Peptides from MDP and CTA”
1 – Tet – “Peptide Tet”
1 – Sterile water for injection
1 – Sterile field
1 – 3-way stopcock (or fluid connector with double female luer locks)
2 – 5 cc glass syringes
1 – Forceps/clamp
9 – Alcohol swabs, 70%
3 – 3 cc plastic syringe
3 – 25-gauge 5/8” needle
1 – 3 ml vial of Montanide ISA-51
2 – 19-gauge1 ½” needle
1 – Small container with de-ionized water
1 – Ziploc bag
95% Ethanol
1 – 50 ml conical tube (sterile)

General Preparation
1. Remove 12 MP and Tet vials from the freezer. Thaw at room temperature (1-2 minutes).
2. Turn on blower in hood.
3. While wearing gloves, clean the hood with sterile gauze pads and 70% ethanol.
4. Lay out the sterile field in the hood.
5. Open the stopcock. Turn the ‘off’ lever to the right (over the male connector). Place on the sterile field.
6. Open the packages containing the glass syringes. Insert the pistons into the barrels, keeping them sterile. Place the syringes on the sterile field.
7. Open the packages of sterile gauze packets. Allow gauze to fall onto sterile field.
8. Lay all other required items inside the hood (not on the sterile field).
9. Wipe forceps or clamp with an alcohol swab.

10. Lift the seal top on the 12 MP and Tet vials using forceps. Remove the protective cover to expose the stopper on the sterile water.
11. Wipe the rubber stopper of the 12 MP, Tet, and sterile water using a new alcohol swab for each vial.
12. Place a 19-gauge needle on a plastic 3 cc syringe. Uncap the needle and withdraw 1.5 ml of sterile water.
13. Inject the 1.5 ml of sterile water into the 12 MP vial. Swirl until dissolved.
14. Using the same 3 cc plastic syringe and needle, withdraw all of the 12 MP solution (1.5 ml).
15. Inject the 1.5 ml of the 12 MP solution into the Tet vial. Swirl until dissolved.
16. Using the same 3 cc plastic syringe and needle, withdraw all of the peptide solution (1.5 ml).
17. Hold one 5 cc glass syringe inverted, with the piston pulled out to approximately 3 ml. Inject the peptide solution into the glass syringe from the 3 cc plastic syringe.
18. Remove the cap from one of the female connectors on the stopcock. Attach the glass syringe containing the peptide solution to the stopcock.
19. Lay the 5 cc glass syringe containing the peptide solution and stopcock on the sterile field.

<u>Montanide Preparation</u>

20. Wipe the neck of the vial of Montanide ISA-51 with an alcohol swab. Snap off the top of the vial.
21. Place a 19-gauge needle on the second 5 cc glass syringe. Uncap the needle and withdraw 1.5 ml of Montanide ISA-51. Inject any excess onto the sterile gauze pad.
22. Remove the cap over the second female connector on the stopcock.
23. Remove the needle from the 5 cc glass syringe containing the Montanide ISA-51.
24. Attach the stopcock and syringe with the peptide solution to the 5 cc glass syringe containing the 1.5 ml of Montanide ISA-51.

<u>Mixing & Testing</u>

25. Hold the syringe-stopcock-syringe apparatus, using your thumbs to hold the pistons.
26. Firmly force the peptide solution into the Montanide ISA-51. Rapidly force the solution back and forth between the syringes for 10 minutes. During mixing, a noticeable difference can be detected once the emulsion is stable.
27. Force all but a few drops of the emulsion into one of the glass syringes.
28. Remove the near empty syringe from the stopcock. Lay the full syringe and stopcock on the sterile field.
29. Attach a 25-gauge needle to the nearly empty syringe.
30. Test the emulsion: add 2 drops (approximately 100-200 μl) of the emulsion to a container of de-ionized water. If the drops disperse in the water, mix for an additional 5 minutes and recheck as described. This test may be repeated twice.

<u>Preparing the vaccine for injection</u>

31. Attach a 3 cc plastic syringe to the open connector, where the nearly empty 5 cc glass syringe was removed.
32. Force 1.2 ml of the emulsion into the 3 cc plastic syringe.

33. Remove the 3 cc syringe from the stopcock. Lay the glass syringe on the sterile field.
34. Attach a 25-gauge needle to the 3 cc plastic syringe.
35. Inject excess emulsion (0.2 ml) onto a sterile gauze pad. Tap on the syringe to void any bubbles.
36. Recap the needle and pull back on the piston slightly.
37. Affix the patient information label to the syringe, and add an additional label inside of the Ziploc bag.
38. Place the syringe into the Ziploc bag for transport, and attach an additional label to the upper right corner of the form.
39. Repeat steps 31 through 38 for two syringes with 1.0 ml of emulsion in each.

Documentation & Clean-up

40. Dispose of the syringes, needles, and glass vials in a contaminated materials container. Dispose of all other packaging in a regular trashcan.
41. Remove the pistons from the syringe barrels. Soak the glass barrels and pistons in 95% ethanol for at least 24 hours. Send syringes for cleaning, packaging, and sterilization.

    The remaining vaccine should be discarded.

The prepared vaccine should be refrigerated until just prior to administration. Ideally, the vaccine should be administered within 1-2 hours after mixing. The vaccine must be administered within 4 hours of mixing. If the vaccine is not administered within 4 hours after mixing, it should be discarded.

Group B: Cyclophosphamide + 12 MP + Tetanus Peptide Vaccine

For administration of Cyclophosphamide (Cytoxan®) on day -4:
Cyclophosphamide will be mixed by the investigational pharmacist in accord with the manufacturer's directions. 300 mg/m^2 of cyclophosphamide will be administered once intravenously in 250 ml of saline over 30-60 minutes.

For vaccinations on days 1, 8, 15, 29, 36, and 43 and weeks 12, 26, 39, and 52:
3 ml of emulsion will be prepared. 2 ml of this emulsion will be administered subcutaneously and intradermally, at two vaccination sites. The remaining 1 ml will be discarded.

2 ml contains 100 mcg each of the 12 peptides listed in Table 1 and 200 mcg of tetanus peptide emulsified in Montanide ISA-51 adjuvant.

Materials
Gloves (sterile)
2-3 sterile gauze packets
70% ethanol
1 – 12 MP – "12 Melanoma Peptides from MDP and CTA"
1 – Tet – "Peptide Tet"
1 – Sterile water for injection
1 – Sterile field
1 – 3-way stopcock (or fluid connector with double female luer locks)
2 – 5 cc glass syringes
1 – Forceps/clamp
9 – Alcohol swabs, 70%
3 – 3 cc plastic syringe
3 – 25-gauge 5/8" needle
1 – 3 ml vial of Montanide ISA-51
2 – 19-gauge1 ½" needle
1 – Small container with de-ionized water
1 – Ziploc bag
95% Ethanol
1 – 50 ml conical tube (sterile)

General Preparation
1. Remove 12 MP and Tet vials from the freezer. Thaw at room temperature (1-2 minutes).
2. Turn on blower in hood.
3. While wearing gloves, clean the hood with sterile gauze pads and 70% ethanol.
4. Lay out the sterile field in the hood.
5. Open the stopcock. Turn the 'off' lever to the right (over the male connector). Place on the sterile field.
6. Open the packages containing the glass syringes. Insert the pistons into the barrels, keeping them sterile. Place the syringes on the sterile field.
7. Open the packages of sterile gauze packets. Allow gauze to fall onto sterile field.
8. Lay all other required items inside the hood (not on the sterile field).
9. Wipe forceps or clamp with an alcohol swab.

10. Lift the seal top on the 12 MP and Tet vials using forceps. Remove the protective cover to expose the stopper on the sterile water.
11. Wipe the rubber stopper of the 12 MP, Tet, and sterile water using a new alcohol swab for each vial.
12. Place a 19-gauge needle on a plastic 3 cc syringe. Uncap the needle and withdraw 1.5 ml of sterile water.
13. Inject the 1.5 ml of sterile water into the 12 MP vial. Swirl until dissolved.
14. Using the same 3 cc plastic syringe and needle, withdraw all of the 12 MP solution (1.5 ml).
15. Inject the 1.5 ml of the 12 MP solution into the Tet vial. Swirl until dissolved.
16. Using the same 3 cc plastic syringe and needle, withdraw all of the peptide solution (1.5 ml).
17. Hold one 5 cc glass syringe inverted, with the piston pulled out to approximately 3 ml. Inject the peptide solution into the glass syringe from the 3 cc plastic syringe.
18. Remove the cap from one of the female connectors on the stopcock. Attach the glass syringe containing the peptide solution to the stopcock.
19. Lay the 5 cc glass syringe containing the peptide solution and stopcock on the sterile field.

<u>Montanide Preparation</u>

20. Wipe the neck of the vial of Montanide ISA-51 with an alcohol swab. Snap off the top of the vial.
21. Place a 19-gauge needle on the second 5 cc glass syringe. Uncap the needle and withdraw 1.5 ml of Montanide ISA-51. Inject any excess onto the sterile gauze pad.
22. Remove the cap over the second female connector on the stopcock.
23. Remove the needle from the 5 cc glass syringe containing the Montanide ISA-51.
24. Attach the stopcock and syringe with the peptide solution to the 5 cc glass syringe containing the 1.5 ml of Montanide ISA-51.

<u>Mixing & Testing</u>

25. Hold the syringe-stopcock-syringe apparatus, using your thumbs to hold the pistons.
26. Firmly force the peptide solution into the Montanide ISA-51. Rapidly force the solution back and forth between the syringes for 10 minutes. During mixing, a noticeable difference can be detected once the emulsion is stable.
27. Force all but a few drops of the emulsion into one of the glass syringes.
28. Remove the near empty syringe from the stopcock. Lay the full syringe and stopcock on the sterile field.
29. Attach a 25-gauge needle to the nearly empty syringe.
30. Test the emulsion: add 2 drops (approximately 100-200 μl) of the emulsion to a container of de-ionized water. If the drops disperse in the water, mix for an additional 5 minutes and recheck as described. This test may be repeated twice.

<u>Preparing the vaccine for injection</u>

31. Attach a 3 cc plastic syringe to the open connector, where the nearly empty 5 cc glass syringe was removed.
32. Force 1.2 ml of the emulsion into the 3 cc plastic syringe.
33. Remove the 3 cc syringe from the stopcock. Lay the glass syringe on the sterile field.
34. Attach a 25-gauge needle to the 3 cc plastic syringe.

35. Inject excess emulsion (0.2 ml) onto a sterile gauze pad. Tap on the syringe to void any bubbles.
36. Recap the needle and pull back on the piston slightly.
37. Affix the patient information label to the syringe, and add an additional label inside of the Ziploc bag.
38. Place the syringe into the Ziploc bag for transport, and attach an additional label to the upper right corner of the form.
39. Repeat steps 31 through 38 for two syringes with 1.0 ml of emulsion in each.

Documentation & Clean-up

40. Dispose of the syringes, needles, and glass vials in a contaminated materials container. Dispose of all other packaging in a regular trashcan.
41. Remove the pistons from the syringe barrels. Soak the glass barrels and pistons in 95% ethanol for at least 24 hours. Send syringes for cleaning, packaging, and sterilization.

The remaining vaccine should be discarded.

The prepared vaccine should be refrigerated until just prior to administration. Ideally, the vaccine should be administered within 1-2 hours after mixing. The vaccine must be administered within 4 hours of mixing. If the vaccine is not administered within 4 hours after mixing, it should be discarded.

Group C: 12 MP + 6 MHP Peptide Vaccine

For vaccinations on days 1, 8, 15, 29, 36, and 43 and weeks 12, 26, 39, and 52: 3 ml of emulsion will be prepared. 2 ml of this emulsion will be administered subcutaneously and intradermally, at two vaccination sites. The remaining 1 ml will be discarded.

2 ml contains 100 mcg each of the 12 peptides listed in Table 1 and 200 mcg each of the 6 peptides listed in Table 2 emulsified in Montanide ISA-51 adjuvant.

<u>Materials</u>
Gloves (sterile)
2-3 sterile gauze packets
70% ethanol
1 – 12 MP – “12 Melanoma Peptides from MDP and CTA”
1 – 6 MHP 300 – “6 Class II MHC-Restricted Melanoma-Associated Peptides” (300 mcg/peptide/vial) or 1 – 6 MHP 600 – “6 Class II MHC-Restricted Melanoma-Associated Peptides” (600 mcg/peptide/vial)
1 – Sterile water for injection
1 – Sterile field
1 – 3-way stopcock (or fluid connector with double female luer locks)
2 – 5 cc glass syringes
1 – Forceps/clamp
9 – Alcohol swabs, 70%
3 – 3 cc plastic syringe
3 – 25-gauge 5/8” needle
1 – 3 ml vial of Montanide ISA-51
2 – 19-gauge 1 ½” needle
1 – Small container with de-ionized water
1 – Ziploc bag
95% Ethanol
1 – 50 ml conical tube (sterile)

<u>General Preparation</u>
1. Remove 12 MP and 6 MHP vials from the freezer. Thaw at room temperature (1-2 minutes).
2. Turn on blower in hood.
3. While wearing gloves, clean the hood with sterile gauze pads and 70% ethanol.
4. Lay out the sterile field in the hood.
5. Open the stopcock. Turn the ‘off’ lever to the right (over the male connector). Place on the sterile field.
6. Open the packages containing the glass syringes. Insert the pistons into the barrels, keeping them sterile. Place the syringes on the sterile field.
7. Open the packages of sterile gauze packets. Allow gauze to fall onto sterile field.
8. Lay all other required items inside the hood (not on the sterile field).
9. Wipe forceps or clamp with an alcohol swab.
10. Lift the seal top on the 12 MP and 6 MHP vials using forceps. Remove the protective cover to expose the stopper on the sterile water.

11. Wipe the rubber stopper of the 12 MP, 6 MHP, and sterile water using a new alcohol swab for each vial.

Peptide Preparation-

If you are using 6 MHP 300 (300 mcg/peptide/vial) follow steps 12-16. If you are using 6 MHP 600 (600 mcg/peptide/vial) follow steps 12a-16a.

Using 6 MHP 300 (300 mcg/peptide/vial)

12. Place a 19-gauge needle on a plastic 3 cc syringe. Uncap the needle and withdraw 1.5 ml of sterile water.
13. Inject the 1.5 ml of sterile water into the 12 MP vial. Swirl until dissolved.
14. Using the same 3 cc plastic syringe and needle, withdraw all of the 12 MP solution (1.5 ml).
15. Inject the 1.5 ml of the 12 MP solution into the 6 MHP 300 vial. Swirl until dissolved.
16. Using the same 3 cc plastic syringe and needle, withdraw all of the peptide solution (1.5 ml).

Using 6 MHP 600 (600 mcg/peptide/vial)

12a. Place a 19-gauge needle on a plastic 3 cc syringe. Uncap the needle and withdraw 3.0 ml of sterile water.

13a. Inject the 3.0 ml of sterile water into the 6 MHP 600 vial. Swirl until dissolved.

14a. Using the same 3 cc plastic syringe and needle, withdraw half of the 6 MHP 600 solution (1.5 ml).
Note: The remaining portion of the 6 MHP 600 vaccine (1.5 mL) should be stored in the original container at -80 °C, and labeled “For Laboratory Use Only.”

15a. Inject the 1.5 ml of the 6 MHP 600 solution into the 12 MP vial. Swirl until dissolved.

16a. Using the same 3 cc plastic syringe and needle, withdraw all of the peptide solution from the 12 MP vial (1.5 ml).

Injecting the Peptide Solution into the Glass Syringes

17. Hold one 5 cc glass syringe inverted, with the piston pulled out to approximately 3 ml. Inject the peptide solution into the glass syringe from the 3 cc plastic syringe.
18. Remove the cap from one of the female connectors on the stopcock. Attach the glass syringe containing the peptide solution to the stopcock.
19. Lay the 5 cc glass syringe containing the peptide solution and stopcock on the sterile field.

Montanide Preparation

20. Wipe the neck of the vial of Montanide ISA-51 with an alcohol swab. Snap off the top of the vial.
21. Place a 19-gauge needle on the second 5 cc glass syringe. Uncap the needle and withdraw 1.5 ml of Montanide ISA-51. Inject any excess onto the sterile gauze pad.
22. Remove the cap over the second female connector on the stopcock.
23. Remove the needle from the 5 cc glass syringe containing the Montanide ISA-51.
24. Attach the stopcock and syringe with the peptide solution to the 5 cc glass syringe containing the 1.5 ml of Montanide ISA-51.

Mixing & Testing

25. Hold the syringe-stopcock-syringe apparatus, using your thumbs to hold the pistons.
26. Firmly force the peptide solution into the Montanide ISA-51. Rapidly force the solution back and forth between the syringes for 10 minutes. During mixing, a noticeable difference can be detected once the emulsion is stable.
27. Force all but a few drops of the emulsion into one of the glass syringes.
28. Remove the near empty syringe from the stopcock. Lay the full syringe and stopcock on the sterile field.
29. Attach a 25-gauge needle to the nearly empty syringe.
30. Test the emulsion: add 2 drops (approximately 100-200 μl) of the emulsion to a container of de-ionized water. If the drops disperse in the water, mix for an additional 5 minutes and recheck as described. This test may be repeated twice.

Preparing the vaccine for injection

31. Attach a 3 cc plastic syringe to the open connector, where the nearly empty 5 cc glass syringe was removed.
32. Force 1.2 ml of the emulsion into the 3 cc plastic syringe.
33. Remove the 3 cc syringe from the stopcock. Lay the glass syringe on the sterile field.
34. Attach a 25-gauge needle to the 3 cc plastic syringe.
35. Inject excess emulsion (0.2 ml) onto a sterile gauze pad. Tap on the syringe to void any bubbles.
36. Recap the needle and pull back on the piston slightly.
37. Affix the patient information label to the syringe, and add an additional label inside of the Ziploc bag.
38. Place the syringe into the Ziploc bag for transport, and attach an additional label to the upper right corner of the form.
39. Repeat steps 31 through 38 for two syringes with 1.0 ml of emulsion in each.

Documentation & Clean-up

40. Dispose of the syringes, needles, and glass vials in a contaminated materials container. Dispose of all other packaging in a regular trashcan.
41. Remove the pistons from the syringe barrels. Soak the glass barrels and pistons in 95% ethanol for at least 24 hours. Send syringes for cleaning, packaging, and sterilization.

The remaining vaccine should be discarded.

The prepared vaccine should be refrigerated until just prior to administration. Ideally, the vaccine should be administered within 1-2 hours after mixing. The vaccine must be administered within 4 hours of mixing. If the vaccine is not administered within 4 hours after mixing, it should be discarded.

Group D: Cyclophosphamide + 12 MP + 6 MHP Peptide Vaccine

For administration of Cyclophosphamide (Cytoxan®) on day -4:
Cyclophosphamide will be mixed by the investigational pharmacist in accord with the manufacturer's directions. 300 mg/m^2 of Cyclophosphamide will be administered once intravenously in 250 ml of saline over 30-60 minutes.

For vaccinations on days 1, 8, 15, 29, 36, and 43 and weeks 12, 26, 39, and 52:
3 ml of emulsion will be prepared. 2 ml of this emulsion will be administered subcutaneously and intradermally, at two vaccination sites. The remaining 1 ml will be discarded.

2 ml contains 100 mcg each of the 12 peptides listed in Table 1 and 200 mcg each of the 6 peptides listed in Table 2, emulsified in Montanide ISA-51 adjuvant.

<u>Materials</u>
Gloves (sterile)
2-3 sterile gauze packets
70% ethanol
1 – 12 MP – "12 Melanoma Peptides from MDP and CTA"
1 – 6 MHP 300 – "6 Class II MHC-Restricted Melanoma-Associated Peptides" (300 mcg/peptide/vial) or 1 – 6 MHP 600 – "6 Class II MHC-Restricted Melanoma-Associated Peptides" (600 mcg/peptide/vial)
1 – Sterile water for injection
1 – Sterile field
1 – 3-way stopcock (or fluid connector with double female luer locks)
2 – 5 cc glass syringes
1 – Forceps/clamp
9 – Alcohol swabs, 70%
3 – 3 cc plastic syringe
3 – 25-gauge 5/8" needle
1 – 3 ml vial of Montanide ISA-51
2 – 19-gauge 1 ½" needle
1 – Small container with de-ionized water
1 – Ziploc bag
95% Ethanol
1 – 50 ml conical tube (sterile)

<u>General Preparation</u>
1. Remove 12 MP and 6 MHP vials from the freezer. Thaw at room temperature (1-2 minutes).
2. Turn on blower in hood.
3. While wearing gloves, clean the hood with sterile gauze pads and 70% ethanol.
4. Lay out the sterile field in the hood.
5. Open the stopcock. Turn the 'off' lever to the right (over the male connector). Place on the sterile field.
6. Open the packages containing the glass syringes. Insert the pistons into the barrels, keeping them sterile. Place the syringes on the sterile field.
7. Open the packages of sterile gauze packets. Allow gauze to fall onto sterile field.
8. Lay all other required items inside the hood (not on the sterile field).

9. Wipe forceps or clamp with an alcohol swab.
10. Lift the seal top on the 12 MP and 6 MHP vials using forceps. Remove the protective cover to expose the stopper on the sterile water.
11. Wipe the rubber stopper of the 12 MP, 6 MHP, and sterile water using a new alcohol swab for each vial.

Peptide Preparation-
If you are using 6 MHP 300 (300 mcg/peptide/vial) follow steps 12-16. If you are using 6 MHP 600 (600 mcg/peptide/vial) follow steps 12a-16a.
Using 6 MHP 300 (300 mcg/peptide/vial)
12. Place a 19-gauge needle on a plastic 3 cc syringe. Uncap the needle and withdraw 1.5 ml of sterile water.
13. Inject the 1.5 ml of sterile water into the 12 MP vial. Swirl until dissolved.
14. Using the same 3 cc plastic syringe and needle, withdraw all of the 12 MP solution (1.5 ml).
15. Inject the 1.5 ml of the 12 MP solution into the 6 MHP 300 vial. Swirl until dissolved.
16. Using the same 3 cc plastic syringe and needle, withdraw all of the peptide solution (1.5 ml).

Using 6 MHP 600 (600 mcg/peptide/vial)
12a. Place a 19-gauge needle on a plastic 3 cc syringe. Uncap the needle and withdraw 3.0 ml of sterile water.
13a. Inject the 3.0 ml of sterile water into the 6 MHP 600 vial. Swirl until dissolved.
14a. Using the same 3 cc plastic syringe and needle, withdraw half of the 6 MHP 600 solution (1.5 ml).
Note: The remaining portion of the 6 MHP 600 vaccine (1.5 mL) should be stored in the original container at -80 °C, and labeled “For Laboratory Use Only.”
15a. Inject the 1.5 ml of the 6 MHP 600 solution into the 12 MP vial. Swirl until dissolved.
16a. Using the same 3 cc plastic syringe and needle, withdraw all of the peptide solution from the 12 MP vial (1.5 ml).

Injecting the Peptide Solution into the Class Syringes
17. Hold one 5 cc glass syringe inverted, with the piston pulled out to approximately 3 ml. Inject the peptide solution into the glass syringe from the 3 cc plastic syringe.
18. Remove the cap from one of the female connectors on the stopcock. Attach the glass syringe containing the peptide solution to the stopcock.
19. Lay the 5 cc glass syringe containing the peptide solution and stopcock on the sterile field.

Montanide Preparation
20. Wipe the neck of the vial of Montanide ISA-51 with an alcohol swab. Snap off the top of the vial.
21. Place a 19-gauge needle on the second 5 cc glass syringe. Uncap the needle and withdraw 1.5 ml of Montanide ISA-51. Inject any excess onto the sterile gauze pad.
22. Remove the cap over the second female connector on the stopcock.
23. Remove the needle from the 5 cc glass syringe containing the Montanide ISA-51.
24. Attach the stopcock and syringe with the peptide solution to the 5 cc glass syringe containing the 1.5 ml of Montanide ISA-51.

Mixing & Testing

25. Hold the syringe-stopcock-syringe apparatus, using your thumbs to hold the pistons.
26. Firmly force the peptide solution into the Montanide ISA-51. Rapidly force the solution back and forth between the syringes for 10 minutes. During mixing, a noticeable difference can be detected once the emulsion is stable.
27. Force all but a few drops of the emulsion into one of the glass syringes.
28. Remove the near empty syringe from the stopcock. Lay the full syringe and stopcock on the sterile field.
29. Attach a 25-gauge needle to the nearly empty syringe.
30. Test the emulsion: add 2 drops (approximately 100-200 μl) of the emulsion to a container of de-ionized water. If the drops disperse in the water, mix for an additional 5 minutes and recheck as described. This test may be repeated twice.

Preparing the vaccine for injection

31. Attach a 3 cc plastic syringe to the open connector, where the nearly empty 5 cc glass syringe was removed.
32. Force 1.2 ml of the emulsion into the 3 cc plastic syringe.
33. Remove the 3 cc syringe from the stopcock. Lay the glass syringe on the sterile field.
34. Attach a 25-gauge needle to the 3 cc plastic syringe.
35. Inject excess emulsion (0.2 ml) onto a sterile gauze pad. Tap on the syringe to void any bubbles.
36. Recap the needle and pull back on the piston slightly.
37. Affix the patient information label to the syringe, and add an additional label inside of the Ziploc bag.
38. Place the syringe into the Ziploc bag for transport, and attach an additional label to the upper right corner of the form.
39. Repeat steps 31 through 38 for two syringes with 1.0 ml of emulsion in each.

Documentation & Clean-up

40. Dispose of the syringes, needles, and glass vials in a contaminated materials container. Dispose of all other packaging in a regular trashcan.
41. Remove the pistons from the syringe barrels. Soak the glass barrels and pistons in 95% ethanol for at least 24 hours. Send syringes for cleaning, packaging, and sterilization.

The remaining vaccine should be discarded.

The prepared vaccine should be refrigerated until just prior to administration. Ideally, the vaccine should be administered within 1-2 hours after mixing. The vaccine must be administered within 4 hours of mixing. If the vaccine is not administered within 4 hours after mixing, it should be discarded.

Appendix 7: NCI Common Terminology Criteria for Adverse Events v3.0
(http://ctep.cancer.gov/forms/CTCAEv3.pdf)

Appendix 8: Immunologic Studies

Delayed Type Hypersensitivity Testing

DTH responses following cutaneous challenge with tumor antigen preparations can be used to monitor the tumor-specific immunity in immunized participants. Prior to the first vaccination, participants will be tested for DTH using a standard small panel of recall antigens that will include tuberculin, tetanus toxoid (when available), trichophyton, and candida. At this time, they will also be tested for a DTH response following challenge with the peptides (100 mcg each) to be used in the immunization protocol. The tests will be placed on the forearm of the arm not to be used for vaccination. If an arm site is not available due to axillary dissections or vaccinations in the arms, then the tests will be placed on a posterior shoulder, buttock, abdomen, or hip. The DTH will be read twice for induration and erythema: at 24 and at 48 hours by a research clinician. The tests will be repeated six weeks following completion of the immunization protocol (day 85). Pre- and post-treatment responses will be compared.

The following grading system will be used to evaluate induration: 1) (-) no induration, 2) (+/-) equivocal induration, 3) (+) ≥ 5 mm induration, 4) (++) ≥ 10 mm induration, 5) (+++) ≥ 20 mm induration. An increase of ≥ 2 levels of induration from pre-immune testing to post-immune testing will be considered an indicator of a positive DTH response to immunization. Responses to control antigens will be assessed to establish immunocompetence.

Preparation of Antigens:

12 Melanoma Peptides (12-MP):
A vial of lyophilized class I MHC-restricted peptides (0.15 mg of each peptide, 1.8 mg total) will be reconstituted using 0.15 ml of sterile water. 0.1 ml of this peptide solution containing 0.1 mg of each peptide (1.2 mg total) will be administered intradermally for DTH testing.

6 Helper Peptides (6-MHP):
A vial of lyophilized helper peptides (0.3 mg of each peptide, 1.8 mg total) will be reconstituted using 0.3 ml of sterile water. 0.1 ml of this peptide solution containing 0.1 mg of each peptide (0.6 mg total) will be administered intradermally for DTH testing.

Tetanus Peptide (Peptide-tet):
A vial of lyophilized tetanus peptide (0.3 mg) will be reconstituted using 0.3 ml of sterile water. 0.1 ml of this peptide solution containing 0.1 mg of tetanus peptide will be administered intradermally for DTH testing.

Tuberculin:
Tuberculin will be purchased from the University of Virginia Pharmacy in individual vials containing 5TU/0.1 ml (Aventis Pasteur, Swiftwater, PA). Administer 0.1 ml (5TU) of this solution intradermally.

Tetanus:
Tetanus toxoid will be purchased from Aventis Pasteur (Swiftwater, PA). The package insert states that each 0.5 ml of the sterile suspension [alum-precipitated toxoid in an isotonic sodium chloride solution containing sodium phosphate] is formulated to contain

5 Lf (flocculation units) of tetanus toxoid. This solution will be diluted 1:5 with buffered saline solution, and 0.1 ml (0.2 Lf) of this diluted solution will be administered intradermally for DTH testing.

Trichophyton:
Trichophyton will be purchased from Hollister-Stier (Spokane, WA). The package insert states the sterile extracts are supplied in dropper vials containing, in addition to the extract allergens and antigens, 50% (v/v) glycerin, 0.5% sodium chloride, and 0.275% sodium bicarbonate. The strength of the trichophyton solution is 1:10 w/v; this solution will be diluted 1:200 with buffered saline, and 0.1 ml of this diluted solution will be administered intradermally for DTH testing.

Candida:
Candida will be purchased from Hollister-Stier (Spokane, WA). The package insert states the sterile extracts are supplied in dropper vials containing, in addition to the extract allergens and antigens, 50% (v/v) glycerin, 0.5% sodium chloride, and 0.275% sodium bicarbonate. The strength of the candida solution is 1:10 weight to volume (w/v); this solution will be diluted 1:200 with buffered saline, and 0.1 ml of this diluted solution will be administered intradermally for DTH testing.

ELIspot assay:
PBL will be evaluated by ELIspot assay for the number of peptide-reactive T-cells per 10^5 cells. PBL will be evaluated before vaccination and at several time points during and after the vaccination regimen. The ELIspot assay measures the number of cells releasing INFγ specifically in response to the vaccination peptides, as recorded by the number of chromogen-defined spots counted directly in the assay wells after exposure of participant lymphocytes to antigen in the presence of solid-phase antibody to IFNγ. The number of spots is compared to two negative controls, one of which is generated by stimulation with an irrelevant peptide. Responder T cells stimulated with PMA, ionomycin, PHA, and/or anti-CD3 will be used as a positive control. Assays will be performed primarily on lymphocytes sensitized once *in vitro*. PBL will be sensitized with the peptide mixture on day 0, and will be assayed using individual peptides at day 14. Participants with reactivity after *in vitro* sensitization will be retested using cryopreserved lymphocytes that have not been sensitized or cultured *ex vivo*.

This ELIspot assay will be performed as follows:
1. Prewet plates with 70% methanol before adding antibody. Dilute capture antibody to 8 mcg/ml in PBS. Coat the wells of Multioscreen-IP sterile plates (Millipore, Bedford, MA) with 50 mcl diluted antibody per well. Antibody is Endogen anti-IFNγ monoclonal antibody # M-700A at 1 mg/ml.
2. Incubate plate overnight at 4°C, then for one hour at room temperature.
3. Flick coating antibody out and wash plate 3X with sterile PBS to remove unbound coating antibody. Block plates with RPMI with 10% human AB serum, Pen-Strep, and Glutamine for 2 hours in incubator at 37°C, 5% CO2. Flick out media and replace with fresh just before adding cells.
4. Add responders to the wells at 75,000 or 25,000 per well in 100 mcl media, mix with 75,000 irradiated stimulator cells alone or stimulator cells pulsed with peptide (40 mcg/ml). Final volume is 300 mcl. Incubate plate at 37°C, 5% CO_2 for 18-20 hours.
5. Wash plate 6X with deionized H_2O + 0.01% Tween 20 with a 5 minute soak between washes.

6. Add biotinylated secondary antibody (50 mcl/well, Endogen M-701B, 1.25 mcg/ml in PBS supplemented with 5% of FBS and 0.05% Tween 20) and incubate for 2 hours at room temperature.
7. Wash plate as described above and add 50 mcl/well of streptavidin conjugated with alkaline phosphatase (13043E, Pharmigen), 1:2500 dilution in PBS + 5% FBS + 0.05% Tween 20. Incubate 1 hour at room temperature.
8. Wash plate as described and develop spots with 50 mcl/well of 1-step NBP/BCIP substrate (Pierce) for 30-45 minutes. Wash substrate solution off with water as soon as spots are developed. Dry plate overnight at room temperature.
9. Count spots using ELIspot reader (Biosys).

For measures of T cell response without *in vitro* sensitization, the ELIspot assay will be performed the same way, except that $CD8^+$ cells will be isolated first by negative selection on an antibody bead column.

Definition of positive response for the ELIspot assays:

ELIspot assay – PBL sensitized once *in vitro*
Among participants who have positive immune responses, the number of responding T-cells varies considerably; therefore, immune response is summarized as a measure of fold change. For ELIspot assays performed on PBL sensitized to peptide mixture once *in vitro*, the response to peptide will be considered positive if the following criteria are met:

1) the ratio of T cell response to an experimental peptide ($T_{exp\ post}$) to T cell response to a control peptide ($T_{con\ post}$) divided by the ratio of pre-vaccination T cell response to an immunizing peptide ($T_{exp\ pre}$) to pre-vaccination T cell response to control ($T_{con\ pre}$) is at least 2,

   $(T_{exp\ post} \div T_{con\ post}) \div (T_{exp\ pre} \div T_{con\ pre}) \geq 2$

   If $(T_{exp\ pre} \div T_{con\ pre}) < 1$, it will be converted to 1 for calculation purposes.

2) the actual number of spots counted for an experimental peptide ($T_{exp\ post}$) is at least 30 (avg spots per 10^5cells) greater than the number of spots counted for a negative control ($T_{con\ post}$), and

3) the number of spots counted for an experimental peptide ($T_{exp\ post}$) minus 1 SD is greater than the number of spots counted for the highest negative control ($T_{con\ post}$) plus 1 SD. Depending upon participant allele type, each participant may generate a T-cell response against more than one of the synthetic peptides.

Note: The threshold for the number of spots over background (30) represents 30 spots per 100,000 lymphocytes, of which approximately 20% are $CD8^+$ cells. Thus, 30 spots per 100,000 lymphocytes represents approximately 30 spots per 20,000 $CD8^+$ cells (0.15%).

ELIspot assay – fresh PBL *ex vivo*
For ELIspot assays performed with cryopreserved fresh lymphocytes, CD8 cell separation will be performed (negative selection) first. The response to peptide will be considered positive if the following criteria are met:

1) the ratio of T cell response to an experimental peptide ($T_{exp\ post}$) to T cell response to a control peptide ($T_{con\ post}$) divided by the ratio of pre-vaccination T cell response to an immunizing peptide ($T_{exp\ pre}$) to pre-vaccination T cell response to control ($T_{con\ pre}$) is at least 2,

   $(T_{exp\ post} \div T_{con\ post}) \div (T_{exp\ pre} \div T_{con\ pre}) \geq 2$

   If $(T_{exp\ pre} \div T_{con\ pre}) < 1$, it will be converted to 1 for calculation purposes.

2) the actual number of spots counted for an experimental peptide ($T_{exp\ post}$) is at least <u>10</u> (avg spots per 10^5 CD8 cells) greater than the number of spots counted for a negative control ($T_{con\ post}$), and

3) the number of spots counted for an experimental peptide ($T_{exp\ post}$) minus 1 SD is greater than the number of spots counted for the highest negative control ($T_{con\ post}$) plus 1 SD. Depending upon participant allele type, each participant may generate a T-cell response against more than one of the synthetic peptides.

Note: The threshold for the number of spots over background (10) represents 10 spots per 100,000 $CD8^+$ lymphocytes. Thus, 10 spots per 100,000 $CD8^+$ lymphocytes represents approximately 0.01%. Background responses tend to be lower in these assays than in assays done after one stimulation.

Tetramer staining

PBL will be evaluated for tetramer binding for assessment of immune response of lymphocyte subpopulations to tumor derived peptide. PBL will be evaluated before vaccination and at several time points during and after the vaccination regimen. In these studies, approximately 200,000 events will be acquired by a FACS Calibur and analyzed by Flowjo software. An irrelevant tetramer will be used as a negative control. Cultured peptide-specific CTL (as determined by Elispot assays) will be used as a positive control.

This assay will be performed as follows:
Assay is performed in polypropylene tubes (Becton Dickinson cat# 352063). Controls for compensation: CD8 (FITC) alone (Beckman Coulter, cat#PN6603861), CD4 (PE) alone (BD Pharmingen, cat#555347), CD3 (APC) alone (BD Pharmingen, cat#555342), 7AAD alone.

1. Aliquot antibodies into master-mix tube, (tetramer sterile!).
2. Pellet cells (10 min 300xg, 18°C) in FACS tube & tip carefully, blotting on towel.
3. Resuspend in 200 mcl PBS.
4. Add antibodies and tetramers.
5. Incubate at room temperature for 30 minutes in the dark.
6. Add 3 ml PBS. Pellet cells (10min 300xg, 18°C). Tip carefully, blotting on towel. Flick to resuspend.
7. Add 100 mcl of diluted 7AAD Solution* to each sample
8. Incubate at 4°C for 20 minutes in dark.
9. Add100 mcl PBS to sample.
10. Pellet cells (10min 300xg, 18°C), tip carefully, blotting on towel.
11. Resuspend in 500 mcl Final Solution**.
12. Store in dark at 4°C. Acquire Flow Cytometry data by FACS Calibur within three days. Software analysis by Flowjo.

*	7AAD Solution:	999 mcl PBS plus 1 mcl of 7AAD stock. 7AAD Stock: 1 mg 7-AAD powder (Calbiochem, cat #129935, San Diego, CA) + 50 mcl absolute methanol directly to the vial. Mix well. Add 950 mcl 1X PBS.
**	Final Solution:	1% paraformaldehyde + 5 mcl/ml stock AD. Stock AD: 1 mg AD powder (Calbiochem, cat#114666), add 50 mcl ice-cold absolute ETOH, vortex, add 950 mcl PBS.
***	Buffer	PBS (Gibco, cat#14190-144)

Proliferation assay

PBL will be evaluated by a proliferation assay to determine whether T cell peptide specific populations are present. PBL will be evaluated before vaccination and at several time points during and after the vaccination regimen. Proliferation will be measured by the incorporation of [^{3}H] thymidine. The specificity of responding T cells will be confirmed by using various negative controls, which may include "no peptide" and an irrelevant peptide that was not incorporated into the vaccine mixture. Responder T cells stimulated with PMA, ionomycin, PHA, and/or anti-CD3 will be used as positive controls.

The proliferation assay will be performed as follows:

1. Samples (approximately 2 x 10^5/well) are cultured in complete media containing 10% human AB serum without any exogenous antigens, with irrelevant class II peptides, or with vaccine-specific peptides.
2. Cells are plated in triplicate or quadruplicate per condition.
3. On day 5 of culture, cells are labeled with [^{3}H] thymidine (approximately 1 mcCi/well; ICN. Costa Mesa, CA) for approximately 8 hours.
4. Plates are harvested and evaluated for [^{3}H] thymidine incorporation.
5. Peptide-specific proliferation is determined as a difference between [^{3}H] thymidine incorporation in experimental wells and the highest negative control.
6. Stimulation indices are calculated.

Cytokine Production Assay

For those participants whose lymphocytes proliferate in response to stimulation with the immunizing peptides, cytokine profiles of their $CD4^+$ T cells will be analyzed and characterized as T_h1 or T_h2 type cells. $CD8^+$ T cells will be depleted from PBL and SIN samples using separation columns (Vancouver, British Columbia, Canada). Cytokine production will be measured using the Bio-Plex Multiplex Cytokine Assay (Bio-rad, Hercules, CA). Cytokines measured may include IL-2, IL-4, IL-5, IL-10, TNF-α, and IFNγ. The specificity of responding T cells will be confirmed by using various negative controls, which may include "no peptide" and an irrelevant peptide that was not incorporated into the vaccine mixture. Responder T cells stimulated with PMA, ionomycin, PHA, and/or anti-CD3 will be used as positive controls.

In addition, IL-7 and IL-15 levels will be measured in the serum pre- and post-chemotherapy (Groups B and D) using the same kit.

The cytokine production assays will be performed in accord with the manufacturer's instructions.

Reference List

(1) Cormier JN, Abati A, Fetsch P, Hijazi YM, Rosenberg SA, Marincola FM, Topalian SI. Comparative analysis of the in vivo expression of tyrosinase, MART-1/Melan-A, and gp100 in metastatic melanoma lesions: implications for immunotherapy. Journal of Immunotherapy 1997; 21(1):27-31.

(2) de Vries TJ, Fourkour A, Wobbes T, Verkroost G, Ruiter DJ, van Muijen GN. Heterogeneous expression of immunotherapy candidate proteins gp100, MART-1, and tyrosinase in human melanoma cell lines and in human melanocytic lesions. Cancer Res 1997; 57(15):3223-3229.

(3) Brasseur F, Rimoldi D, Lienard D, Lethe B, Carrel S, Arienti F, Suter L, Vanwijck R, Bourlond A, Humblet Y. Expression of MAGE genes in primary and metastatic cutaneous melanoma. Int J Cancer 1995; 63(3):375-380.

(4) Thurner B, Haendle I, Roder C, Dieckmann D, Keikavoussi P, Jonuleit H, Bender A, Maczek C, Schreiner D, von den Driesch P, Brocker EB, Steinman RM, Enk A, Kampgen E, Schuler G. Vaccination with mage-3A1 peptide-pulsed mature, monocyte-derived dendritic cells expands specific cytotoxic T cells and induces regression of some metastases in advanced stage IV melanoma. J Exp Med 1999; 190(11):1669-1678.

(5) Riker A, Cormier J, Panelli M, Kammula U, Wang E, Abati A, Fetsch P, Lee KH, Steinberg S, Rosenberg S, Marincola F. Immune selection after antigen-specific immunotherapy of melanoma. Surgery 1999; 126(2):112-120.

(6) Shankaran V, Ikeda H, Bruce AT, White JM, Swanson PE, Old LJ, Schreiber RD. IFNgamma and lymphocytes prevent primary tumour development and shape tumour immunogenicity. Nature 2001; 410(6832):1107-1111.

(7) Fearon ER, Pardoll DM, Itaya T, Golumbek P, Levitsky HI, Simons JW, Karasuyama H, Vogelstein B, Frost P. Interleukin-2 production by tumor cells bypasses T helper function in the generation of an antitumor response. Cell 1990; 60:397-403.

(8) Golumbek PT, Lazenby AJ, Levitsky HI, Jaffee LM, Karasuyama H, Baker M, Pardoll DM. Treatment of established renal cancer by tumor cells engineered to secrete interleukin-4. Science 1991; 254:713-716.

(9) Dranoff G, Jaffee E, Lazenby A, Golumbek P, Levitsky H, Brose K, Jackson V, Hamada H, Pardoll D, Mulligan RC. Vaccination with irradiated tumor cells engineered to secrete murine granulocyte-macrophage colony-stimulating factor stimulates potent, specific, and long-lasting anti-tumor immunity. Proc Natl Acad Sci USA 1993; 90(8):3539-3543.

(10) Lin K-Y, Guarnieri FG, Stavelyey-O'Carroll KF, Levitsky HI, August JT, Pardoll DM, Wu T-C. Treatment of established tumors with a novel vaccine that enhances major histocompatibility class II presentation of tumor antigen. Cancer Res 1996; 56:21-26.

(11) Kast WM, Offringa R, Peters PJ, Voordouw AC, Meloen RH, van der Eb AJ, Melief CJ. Eradication of adenovirus E1-induced tumors by E1A-specific cytotoxic T lymphocytes. Cell 1989; 59:603-614.

(12) Greenberg PD, Riddell SR. Principles for adoptive T cell therapy of human viral diseases. Annual Review in Immunology 1995; 13:545-586.

(13) Renkvist N, Castelli C, Robbins PF, Parmiani G. A listing of human tumor antigens recognized by T cells. Cancer Immunol Immunother 2001; 50(1):3-15.

(14) Kawakami Y, Robbins PF, Wang RF, Parkhurst M, Kang X, Rosenberg SA. The use of melanosomal proteins in the immunotherapy of melanoma. J Immunotherapy 1998; 21(4):237-246.

(15) Nestle FO, Alijagic S, Gilliet M, Sun Y, Grabbe S, Dummer R, Burg G, Schadendorf D. Vaccination of melanoma patients with peptide- or tumor lysate-pulsed dendritic cells [see comments]. Nat Med 1998; 4(3):328-332.

(16) Rosenberg SA, Yang JC, Schwartzentruber DJ, Hwu P, Marincola FM, Topalian SI, Restifo NP, Dudley ME, Schwarz SI, Spiess PJ, Wunderlich JR, Parkhurst MR,

Kawakami Y, Seipp CA, Einhorn JH, White DE. Immunologic and therapeutic evaluation of a synthetic peptide vaccine for the treatment of patients with metastatic melanoma [see comments]. Comment in: Nat Med 1998 Mar;4(3):269-70. Nat Med 1998; 4(3):321-327.

(17) Slingluff CL, Jr., Petroni GR, Yamshchikov GV, Barnd DL, Eastham S, Galavotti H, Patterson JW, Deacon DH, Hibbitts S, Teates D, Neese PY, Grosh WW, Chianese-Bullock KA, Woodson EM, Wiernasz CJ, Merrill P, Gibson J, Ross M, Engelhard VH. Clinical and immunologic results of a randomized phase II trial of vaccination using four melanoma peptides either administered in granulocyte-macrophage colony-stimulating factor in adjuvant or pulsed on dendritic cells. J Clin Oncol 2003; 21(21):4016-4026.

(18) Jaeger E, Bernhard H, Romero P, Ringhoffer M, Arand M, Karbach J, Ilsemann C, Hagedorn M, Knuth A. Generation of cytotoxic T-cell responses with synthetic melanoma-associated peptides in vivo: implications for tumor vaccines with melanoma-associated antigens. Int J Cancer 1996; 66(2):162-9.

(19) Hu X, Chakraborty NG, Sporn JR, Kurtzman SH, Ergin MT, Mukherji B. Enhancement of cytolytic T lymphocyte precursor frequency in melanoma patients following immunization with the MAGE-1 peptide loaded antigen presenting cell-based vaccine. Cancer Res 1996; 56(11):2479-83.

(20) Chaux P, Luiten R, Demotte N, Vantomme V, Stroobant V, Traversari C, Russo V, Schultz E, Cornelis GR, Boon T, van der Bruggen P. Identification of five MAGE-A1 epitopes recognized by cytolytic T lymphocytes obtained by in vitro stimulation with dendritic cells transduced with MAGE-A1. J Immunol 1999; 163(5):2928-2936.

(21) Kawakami Y, Robbins PF, Wang X, Tupesis JP, Parkhurst MR, Kang X, Sakaguchi K, Appella E, Rosenberg SA. Identification of new melanoma epitopes on melanosomal proteins recognized by tumor infiltrating T lymphocytes restricted by HLA-A1, -A2, and -A3 alleles. J Immunol 1998; 161(12):6985-6992.

(22) Wang RF, Johnston SL, Zeng G, Topalian Sl, Schwartzentruber DJ, Rosenberg SA. A breast and melanoma-shared tumor antigen: T cell responses to antigenic peptides translated from different open reading frames. J Immunol 1998; 161(7):3598-3606.

(23) Huang LQ, Brasseur F, Serrano A, De Plaen E, van der Bruggen P, Boon T, Van Pel A. Cytolytic T lymphocytes recognize an antigen encoded by MAGE-A10 on a human melanoma. J Immunol 1999; 162(11):6849-6854.

(24) Sette A, Sidney J. HLA supertypes and supermotifs: a functional perspective on HLA polymorphism. Current Opinion in Immunology 1900; 10(4):478-482.

(25) Bullock TNJ, Colella TA, Engelhard VH. The density of peptides displayed by dendritic cells affects immune responses to human tyrosinase and gp100 in HLA-A2 transgenic mice. J Immunol 2000; 164:2354-2361.

(26) Kayaga J, Souberbielle BE, Sheikh N, Morrow WJ, Scott-Taylor T, Vile R, Chong H, Dalgleish AG. Anti-tumour activity against B16-F10 melanoma with a GM-CSF secreting allogeneic tumour cell vaccine. [erratum appears in Gene Ther 1999 Nov;6(11):1905]. Gene Therapy 1999; 6(8):1475-1481.

(27) Kahn M, Sugawara H, McGowan P, Okuno K, Nagoya S, Hellstrom KE, Hellstrom I, Greenberg P. CD4+ T cell clones specific for the human p97 melanoma-associated antigen can eradicate pulmonary metastases from a murine tumor expressing the p97 antigen. J Immunol 1991; 146(9):3235-3241.

(28) Weiss WR, Sedegah M, Berzofsky JA, Hoffman SL. The role of CD4+ T cells in immunity to malaria sporozoites. J Immunol 1993; 151(5):2690-2698.

(29) Hung K, Hayashi R, Lafond-Walker A, Lowenstein C, Pardoll D, Levitsky H. The central role of $CD4^+$ T cells in the antitumor immune response. Journal of Experimental Medicine 188, 2357-2368. 1998.

(30) Matsui S, Ahlers JD, Vortmeyer AO, Terabe M, Tsukui T, Carbone DP, Liotta LA, Berzofsky JA. A model for CD8+ CTL tumor immunosurveillance and regulation of tumor escape by CD4 T cells through an effect on quality of CTL. J Immunol 1999; 163(1):184-193.

(31) Slingluff CLJ, Yamshchikov G, Neese P, Galavotti H, Eastham S, Engelhard VH,

Kittlesen D, Deacon D, Hibbitts S, Grosh WW, Petroni G, Cohen R, Wiernasz C, Patterson JW, Conway BP, Ross WG. Phase I trial of a melanoma vaccine with gp100(280-288) peptide and tetanus helper peptide in adjuvant: immunologic and clinical outcomes. Clin Cancer Res 2001; 7(10):3012-3024.

(32) Berd D, Maguire HC, Jr., Mastrangelo MJ. Induction of cell-mediated immunity to autologous melanoma cells and regression of metastases after treatment with a melanoma cell vaccine preceded by cyclophosphamide. Cancer Res 1986; 46(5):2572-2577.

(33) Berd D, Maguire HC, Jr., Mastrangelo MJ. Potentiation of human cell-mediated and humoral immunity by low-dose cyclophosphamide. Cancer Res 1984; 44(11):5439-5443.

(34) Proietti E, Greco G, Garrone B, Baccarini S, Mauri C, Venditti M, Carlei D, Belardelli F. Importance of cyclophosphamide-induced bystander effect on T cells for a successful tumor eradication in response to adoptive immunotherapy in mice. J Clin Invest 1998; 101(2):429-441.

(35) Machiels JP, Reilly RT, Emens LA, Ercolini AM, Lei RY, Weintraub D, Okoye FI, Jaffee EM. Cyclophosphamide, doxorubicin, and paclitaxel enhance the antitumor immune response of granulocyte/macrophage-colony stimulating factor-secreting whole-cell vaccines in HER-2/neu tolerized mice. Cancer Res 2001; 61(9):3689-3697.

(36) Sahasrabudhe DM, deKernion JB, Pontes JE, Ryan DM, O'Donnell RW, Marquis DM, Mudholkar GS, McCune CS. Specific immunotherapy with suppressor function inhibition for metastatic renal cell carcinoma. J Biol Response Mod 1986; 5(6):581-594.

(37) Matar P, Rozados VR, Gervasoni SI, Scharovsky GO. Th2/Th1 switch induced by a single low dose of cyclophosphamide in a rat metastatic lymphoma model. Cancer Immunol Immunother 2002; 50(11):588-596.

(38) Matar P, Rozados VR, Gonzalez AD, Dlugovitzky DG, Bonfil RD, Scharovsky OG. Mechanism of antimetastatic immunopotentiation by low-dose cyclophosphamide. Eur J Cancer 2000; 36(8):1060-1066.

(39) Schiavoni G, Mattei F, Di Pucchio T, Santini SM, Bracci L, Belardelli F, Proietti E. Cyclophosphamide induces type I interferon and augments the number of CD44(hi) T lymphocytes in mice: implications for strategies of chemoimmunotherapy of cancer. Blood 2000; 95(6):2024-2030.

(40) Awwad M, North RJ. Cyclophosphamide (Cy)-facilitated adoptive immunotherapy of a Cy-resistant tumour. Evidence that Cy permits the expression of adoptive T-cell mediated immunity by removing suppressor T cells rather than by reducing tumour burden. Immunology 1988; 65(1):87-92.

(41) Berd D, Mastrangelo MJ. Effect of low dose cyclophosphamide on the immune system of cancer patients: depletion of CD4+, 2H4+ suppressor-inducer T-cells. Cancer Res 1988; 48(6):1671-1675.

(42) Hoon DS, Foshag LJ, Nizze AS, Bohman R, Morton DI. Suppressor cell activity in a randomized trial of patients receiving active specific immunotherapy with melanoma cell vaccine and low dosages of cyclophosphamide. Cancer Res 1990; 50(17):5358-5364.

(43) North RJ. Cyclophosphamide-facilitated adoptive immunotherapy of an established tumor depends on elimination of tumor-induced suppressor T cells. J Exp Med 1982; 155(4):1063-1074.

(44) Dudley ME, Wunderlich JR, Yang JC, Hwu P, Schwartzentruber DJ, Topalian SI, Sherry RM, Marincola FM, Leitman SF, Seipp CA, Rogers-Freezer L, Morton KE, Nahvi A, Mavroukakis SA, White DE, Rosenberg SA. A phase I study of nonmyeloablative chemotherapy and adoptive transfer of autologous tumor antigen-specific T lymphocytes in patients with metastatic melanoma. J Immunother 2002; 25(3):243-251.

(45) Dudley ME, Wunderlich JR, Robbins PF, Yang JC, Hwu P, Schwartzentruber DJ, Topalian SI, Sherry R, Restifo NP, Hubicki AM, Robinson MR, Raffeld M, Duray P, Seipp CA, Rogers-Freezer L, Morton KE, Mavroukakis SA, White DE, Rosenberg SA. Cancer regression and autoimmunity in patients after clonal repopulation with antitumor

lymphocytes. Science 2002; 298(5594):850-854.
(46) Berd D, Maguire HC, Jr., Mastrangelo MJ. Impairment of concanavalin A-inducible suppressor activity following administration of cyclophosphamide to patients with advanced cancer. Cancer Res 1984; 44(3):1275-1280.
(47) Berd D, Mastrangelo MJ. Effect of low dose cyclophosphamide on the immune system of cancer patients: reduction of T-suppressor function without depletion of the CD8+ subset. Cancer Res 1987; 47(12):3317-3321.
(48) Prlic M, Jameson SC. Homeostatic expansion versus antigen-driven proliferation: common ends by different means? Microbes Infect 2002; 4(5):531-537.
(49) Khaled AR, Durum SK. Lymphocide: cytokines and the control of lymphoid homeostasis. Nat Rev Immunol 2002; 2(11):817-830.
(50) Goldrath AW. Maintaining the status quo: T-cell homeostasis. Microbes Infect 2002; 4(5):539-545.
(51) Schluns KS, Lefrancois L. Cytokine control of memory T-cell development and survival. Nat Rev Immunol 2003; 3(4):269-279.
(52) Altman JD, Moss PAH, Goulder PJR, Barouch DH, McHeyzer-Williams MG, Bell JI, McMichael AJ, avis MM. Phenotypic analysis of antigen-specific T lymphocytes [published erratum appears in Science 1998 Jun 19;280(5371):1821]. Science 1996; 274(5284):94-6.
(53) Lee PP, Yee C, Savage PA, Fong L, Brockstedt D, Weber JS, Johnson D, Swetter S, Thompson J, Greenberg PD, Roederer M, Davis MM. Characterization of circulating T cells specific for tumor-associated antigens in melanoma patients. Nat Med 1999; 5(6):677-685.
(54) Herr W, Schneider J, Lohse AW, Meyer zum Buschenfelde KH, Wolfel T. Detection and quantification of blood-derived CD8+ T lymphocytes secreting tumor necrosis factor alpha in response to HLA-A2.1-binding melanoma and viral peptide antigens. J Immunol Methods 1996; 191(2):131-42.
(55) Lalvani A, Brookes R, Hambleton S, Britton WJ, Hill AV, McMichael AJ. Rapid effector function in CD8+ memory T cells. J Exp Med 1997; 186(6):859-865.
(56) Reynolds SR, Oratz R, Shapiro RL, Hao P, Yun Z, Fotino M, Vukmanovic S, Bystryn JC. Stimulation of CD8+ T cell responses to MAGE-3 and Melan A/MART-1 by immunization to a polyvalent melanoma vaccine. Int J Cancer 1997; 72(6):972-976.
(57) Scheibenbogen C, Lee KH, Mayer S, Stevanovic S, Moebius U, Herr W, Rammensee HG, Keilholz U. A sensitive ELISPOT assay for detection of CD8+ T lymphocytes specific for HLA class I-binding peptide epitopes derived from influenza proteins in the blood of healthy donors and melanoma patients. Clin Cancer Res 1997; 3(2):221-226.
(58) Tanchot C, Guillaume S, Delon J, Bourgeois C, Franzke A, Sarukhan A, Trautmann A, Rocha B. Modifications of CD8+ T cell function during in vivo memory or tolerance induction. Immunity 1998; 8(5):581-590.
(59) Yamshchikov GV, Barnd DL, Eastman S, Galavotti HS, Patterson JW, Deacon DH, Teates D, Neese P, Grosh WW, Petroni G, Engelhard VH, Slingluff CL, Jr. Evaluation of peptide vaccine immunogenicity in draining lymph nodes and peripheral blood of melanoma patients. Int J Cancer 2001; 92:703-711.
(60) Yamshchikov G, Thompson L, Ross WG, Galavotti H, Aquila W, Deacon D, Caldwell J, Patterson JW, Hunt DF, Slingluff CL, Jr. Analysis of a natural immune response against tumor antigens in a melanoma survivor: lessons applicable to clinical trial evaluations. Clin Cancer Res 2001; 7(3 Suppl):909s-916s.
(61) Bast RC, Jr., Reinherz EL, Maver C, Lavin P, Schlossman SF. Contrasting effects of cyclophosphamide and prednisolone on the phenotype of human peripheral blood leukocytes. Clin Immunol Immunopathol 1983; 28(1):101-114.
(62) Berd D, Sato T, Maguire HC, Jr., Kairys J, Mastrangelo MJ. Immunopharmacologic analysis of an autologous, hapten-modified human melanoma vaccine. J Clin Oncol 2004; 22(3):403-415.
(63) Slingluff CL, Jr., Petroni GR, Yamshchikov GV, Hibbitts S, Grosh WW, Chianese-Bullock KA, Bissonette EA, Barnd DL, Deacon DH, Patterson JW, Parekh J, Neese PY,

Woodson EM, Wiernasz CJ, Merrill P. Immunologic and clinical outcomes of vaccination with a multiepitope melanoma peptide vaccine plus low-dose interleukin-2 administered either concurrently or on a delayed schedule. J Clin Oncol 2004; 22(22):4474-4485.
(64) Rosenberg SA. A new era for cancer immunotherapy based on the genes that encode cancer antigens. [Review] [79 refs]. Immunity 1999; 10(3):281-287.
(65) Salgaller ML, Marincola FM, Cormier JN, Rosenberg SA. Immunization against epitopes in the human melanoma antigen gp100 following patient immunization with synthetic peptides. Cancer Res 1996; 56(20):4749-4757.
(66) Parkhurst MR, Salgaller ML, Southwood S, Robbins PF, Sette A, Rosenberg SA, Kawakami Y. Improved induction of melanoma-reactive CTL with peptides from the melanoma antigen gp100 modified at HLA-A*0201-binding residues. J Immunol 1996; 157(6):2539-2548.
(67) Cox AL, Skipper J, Chen Y, Henderson RA, Darrow TL, Shabanowitz J, Engelhard VH, Hunt DF, Slingluff CL, Jr. Identification of a peptide recognized by five melanoma-specific human cytotoxic T cell lines. Science 1994; 264(5159):716-9.
(68) Skipper JC, Kittlesen DJ, Hendrickson RC, Deacon DD, Harthun Nl, Wagner SN, Hunt DF, Engelhard VH, Slingluff CL, Jr. Shared epitopes for HLA-A3-restricted melanoma-reactive human CTL include a naturally processed epitope from Pmel-17/gp100. J Immunol 1996; 157(11):5027-5033.
(69) Kawakami Y, Eliyahu S, Jennings C, Sakaguchi K, Kang X, Southwood S, Robbins PF, Sette A, Appella E, Rosenberg SA. Recognition of multiple epitopes in the human melanoma antigen gp100 by tumor-infiltrating T lymphocytes associated with in vivo tumor regression. J Immunol 1995; 154(8):3961-8.
(70) Kittlesen DJ, Thompson LW, Gulden PH, Skipper JC, Colella TA, Shabanowitz JA, Hunt DF, Engelhard VH, Slingluff CL, Jr. Human melanoma patients recognize an HLA-A1-restricted CTL epitope from tyrosinase containing two cysteine residues: implications for tumor vaccine development. J Immunol 1998; 160(5):2099-2106.
(71) Skipper JC, Hendrickson RC, Gulden PH, Brichard V, Van Pel A, Chen Y, Shabanowitz J, Wolfel T, Slingluff CL, Jr., Boon T, Hunt DF, Engelhard VH. An HLA-A2-restricted tyrosinase antigen on melanoma cells results from posttranslational modification and suggests a novel pathway for processing of membrane proteins. J Exp Med 1996; 183(2):527-534.
(72) Traversari C, van der Bruggen P, Luescher IF, Lurquin C, Chomez P, Van Pel A, De Plaen E, Amar-Costesec A, Boon T. A nonapeptide encoded by human gene MAGE-1 is recognized on HLA-A1 by cytolytic T lymphocytes directed against tumor antigen MZ2-E. J Exp Med 1992; 176(5):1453-1457.
(73) Mukherji B, Chakraborty NG, Yamasaki S, Okino T, Yamase H, Sporn JR, Kurtzman SK, Ergin MT, Ozols J, Meehan J. Induction of antigen-specific cytolytic T cells in situ in human melanoma by immunization with synthetic peptide-pulsed autologous antigen presenting cells. Proc Natl Acad Sci USA 1995; 92(17):8078-8082.
(74) Gaugler B, Van den Eynde B, van der Bruggen P, Romero P, Gaforio JJ, De Plaen E, Lethe B, Brasseur F, Boon T. Human gene MAGE-3 codes for an antigen recognized on a melanoma by autologous cytolytic T lymphocytes. J Exp Med 1994; 179(3):921-930.
(75) Valmori D. Use of HLA-A2/peptide tetramers for the analysis of CD8+ T-cell responses to CT antigens in melanoma patients. Cancer Vaccines Meeting (October 2-4, 2000), sponsored by the Cancer Research Institute , S12. 2000.
(76) Shirai M, Pendleton CD, Ahlers J, Takeshita T, Newman M, BerzofskyJ.A. Helper-cytotoxic T lymphocyte (CTL) determinant linkage required for priming of anti-HIV CD8+ CTL in vivo with peptide vaccine constructs. J Immunol 1994; 152:549-556.
(77) Demotz S. Delineation of several DR-restricted tetanus toxin T cell epitopes. J Immunol 1989; 142:394-402.
(78) Topalian Sl, Gonzales MI, Parkhurst M, Li YF, Southwood S, Sette A, Rosenberg SA, Robbins PF. Melanoma-specific $CD4^+$ T cells recognize nonmutated HLA-DR-restricted

tyrosinase epitopes. J Exp Med 1996; 183:1965-1971.
(79) Kobayashi H, Kokubo T, Sato K, Kimura S, Asano K, Takahashi H, Iizuka H, Miyokawa N, Katagiri M. CD4+ T cells from peripheral blood of a melanoma patient recognize peptides derived from nonmutated tyrosinase. Cancer Res 1998; 58:296-301.
(80) Touloukian CE, Leitner WW, Topalian Sl, Li YF, Robbins PF, Rosenberg SA, Restifo NP. Identification of a MHC class II-restricted human gp100 epitope using DR4-IE transgenic mice. J Immunol 2000; 164(7):3535-3542.
(81) Halder T, Pawelec G, Kirkin AF, Zeuthen J, Meyer HE, Kun L, Kalbacher H. Isolation of novel HLA-DR restricted potential tumor-associated antigens from the melanoma cell line FM3. Cancer Res 1997; 57:3238-3244.
(82) Li K, Adibzadeh M, Halder T, Kalbacher H, Heinzel S, Müller C, Zeuthen J, Pawelec G. Tumor-specific MHC-class-II-restricted responses after in vitro sensitization to synthetic peptides corresponding to gp100 and Annexin II eluted from melanoma cells. Cancer Immunol Immunother 1998; 47:32-38.
(83) Manici S, Sturniolo T, Imro MA, Hammer J, Sinigaglia F, Noppen C, Spagnoli G, Mazzi B, Bellone M, Dellabona P, Protti MP. Melanoma cells present a MAGE-3 epitope to $CD4^+$ cytotoxic T cells in association with histocompatibility leukocyte antigen DR11. J Exp Med 1999; 189:871-876.
(84) Chaux P, Vantomme V, Stroobant V, Thielemans K, Corthals J, Luiten R, Eggermont AMM, Boon T, van der Bruggen P. Identification of MAGE-3 epitopes presented by HLA-DR molecules to CD4+ T lymphocytes. J Exp Med 1999; 189:767-777.
(85) Scalzo AA, Elliott SL, Cox J, Gardner J, Moss DJ, Suhrbier A. Induction of protective cytotoxic T cells to murine cytomegalovirus by using a nonapeptide and a human-compatible adjuvant (Montanide ISA 720). Journal of Virology 1995; 69(2):1306-1309.
(86) Fernandez IM, Snijders A, Benaissa-Trouw BJ, Harmsen M, Snippe H, Kraaijeveld CA. Influence of epitope polarity and adjuvants on the immunogenicity and efficacy of a synthetic peptide vaccine against Semliki Forest virus. Journal of Virology 1993; 67(10):5843-8.
(87) Ahlers JD, Dunlop N, Alling DW, Nara Pl, Berzofsky JA. Cytokine-in-adjuvant steering of the immune response phenotype to HIV-1 vaccine constructs: granulocyte-macrophage colony-stimulating factor and TNF-alpha synergize with IL-12 to enhance induction of cytotoxic T lymphocytes. J Immunol 1997; 158(8):3947-58.
(88) Kirkwood JM, Strawderman MH, Ernstoff MS, Smith TJ, Borden EC, Blum RH. Interferon alfa-2b adjuvant therapy of high-risk resected cutaneous melanoma: the Eastern Cooperative Oncology Group Trial EST 1684 [see comments]. J Clin Oncol 1996; 14(1):7-17.
(89) Kirkwood JM, Ibrahim JG, Sondak VK, Richards J, Flaherty LE, Ernstoff MS, Smith TJ, Rao U, Steele M, Blum RH. High- and low-dose interferon alfa-2b in high-risk melanoma: first analysis of intergroup trial E1690/S9111/C9190. J Clin Oncol 2000; 18(12):2444-2458.
(90) Kirkwood JM, Ibrahim JG, Sosman JA, Sondak VK, Agarwala SS, Ernstoff MS, Rao U. High-dose interferon alfa-2b significantly prolongs relapse-free and overall survival compared with the GM2-KLH/QS-21 vaccine in patients with resected stage IIB-III melanoma: results of intergroup trial E1694/S9512/C509801. J Clin Oncol 2001; 19(9):2370-2380.
(91) Kawakami Y. Immunobiology of human melanoma antigens MART-1 and gp100 and their use for Immuno-gene therapy. Int Rev Immunol 1997; 142:173-192.
(92) Balch CM, Buzaid AC, Soong SJ, Atkins MB, Cascinelli N, Coit DG, Fleming ID, Gershenwald JE, Houghton AJ, Kirkwood JM, McMasters KM, Mihm MF, Morton Dl, Reintgen DS, Ross MI, Sober A, Thompson JA, Thompson JF. Final version of the American Joint Committee on Cancer staging system for cutaneous melanoma. [Review] [83 refs]. J Clin Oncol 2001; 19(16):3635-3648.
(93) Mosteller RD. Simplified calculation of body-surface area. N Engl J Med 1987; 317(17):1098.
(94) Robertson T, Wright FT, Dykstra RL. Ordered Restricted Statistical Inference. New

York: John Wiley & Sons, 1988.
(95) Feltz CJ, Dykstra RL. Maximum-Likelihood Estimation of the Survival Functions of N-Stochastically Ordered Random-Variables. Journal of the American Statistical Association 1985; 80(392):1012-1019.
(96) Song X, Davidian M, Tsiatis AA. A semiparametric likelihood approach to joint modeling of longitudinal and time-to-event data. Biometrics 2002; 58(4):742-753.
