# Supplementary material for: Multipeptide vaccines for melanoma in the adjuvant setting: long-term survival outcomes and post-hoc analysis of a randomized phase II trial
Source: Nat Commun. 2024 Mar 22;15:2570. doi: 10.1038/s41467-024-46877-6 (PMC10959948; doi:10.1038/s41467-024-46877-6)
Supplement: Supplementary file 3 — Reporting Summary [file 41467_2024_46877_MOESM3_ESM.pdf]

nature portfolio

Corresponding author(s): Craig L. Slingluff Jr.

Last updated by author(s): Mar 6, 2024

Reporting Summary

Nature Portfolio wishes to improve the reproducibility of the work that we publish. This form provides structure for consistency and transparency in reporting. For further information on Nature Portfolio policies, see our Editorial Policies and the Editorial Policy Checklist.

Statistics

For all statistical analyses, confirm that the following items are present in the figure legend, table legend, main text, or Methods section.

n/a	Confirmed	
☐	☒	The exact sample size (*n*) for each experimental group/condition, given as a discrete number and unit of measurement
☐	☒	A statement on whether measurements were taken from distinct samples or whether the same sample was measured repeatedly
☐	☒	The statistical test(s) used AND whether they are one- or two-sided<br>*Only common tests should be described solely by name; describe more complex techniques in the Methods section.*
☐	☒	A description of all covariates tested
☐	☒	A description of any assumptions or corrections, such as tests of normality and adjustment for multiple comparisons
☐	☒	A full description of the statistical parameters including central tendency (e.g. means) or other basic estimates (e.g. regression coefficient) AND variation (e.g. standard deviation) or associated estimates of uncertainty (e.g. confidence intervals)
☐	☒	For null hypothesis testing, the test statistic (e.g. *F*, *t*, *r*) with confidence intervals, effect sizes, degrees of freedom and *P* value noted<br>*Give P values as exact values whenever suitable.*
☒	☐	For Bayesian analysis, information on the choice of priors and Markov chain Monte Carlo settings
☒	☐	For hierarchical and complex designs, identification of the appropriate level for tests and full reporting of outcomes
☒	☐	Estimates of effect sizes (e.g. Cohen's *d*, Pearson's *r*), indicating how they were calculated

Our web collection on statistics for biologists contains articles on many of the points above.

Software and code

Policy information about availability of computer code

Data collection	Outcome data were stored in OnCore software and entered into an Excel file for this analysis.
Data analysis	No custom algorithms or software were used. Fisher's exact tests larger than 2x2 were calculated using R version 4.2.2 and RStudio v2022.12.0 (Build 353). All other statistical analyses were performed using MedCalc® Statistical Software version 22.016 (MedCalc Software Ltd, Ostend, Belgium; https://www.medcalc.org; 2023).

For manuscripts utilizing custom algorithms or software that are central to the research but not yet described in published literature, software must be made available to editors and reviewers. We strongly encourage code deposition in a community repository (e.g. GitHub). See the Nature Portfolio guidelines for submitting code & software for further information.

Data

Policy information about availability of data

All manuscripts must include a data availability statement. This statement should provide the following information, where applicable:

- Accession codes, unique identifiers, or web links for publicly available datasets
- A description of any restrictions on data availability
- For clinical datasets or third party data, please ensure that the statement adheres to our policy

Source data are now provided with this paper and include individual de-identified participant data. The clinical trial protocol is also provided as Supplementary text.

We have revised the Data Availability statement to specify this.

Research involving human participants, their data, or biological material

Policy information about studies with human participants or human data. See also policy information about sex, gender (identity/presentation), and sexual orientation and race, ethnicity and racism.

Reporting on sex and gender	Impact of sex on outcomes is a primary focus of this manuscript.
Reporting on race, ethnicity, or other socially relevant groupings	Melanoma is a disease that primarily afflicts fair-skinned individuals. All participants were white, and only 1.2% were Hispanic. The numbers were not large enough for meaningful assessment of their impact.
Population characteristics	167 eligible participants from 3 centers, with median age 58 years. Details are in the manuscript.
Recruitment	Participants were recruited at 3 centers with informed consent, and with no known bias. We have specified in the manuscript that they were randomized 1:1:1:1 among the 4 treatment arms and that randomization was stratified to treating institution and HLA type.
Ethics oversight	We have added information here as paragraph 1 of the Methods, which now states:<br><br>"This research study complies with all relevant ethical regulations: the clinical trial MEL44 was performed with approval of the institutional review boards (IRB) at the 3 participating institutions (University of Virginia, MD Anderson Cancer Center at the University of Texas – Houston, and Fox Chase Cancer Center), with the University of Virginia at the lead institution (IRB-HSR #11491). It was also performed with FDA approval (IND #12191) and is registered with ClinicalTrials.gov (NCT00118274). Participants gave informed consent prior to participating in this trial. The study design and conduct complied with all relevant regulations regarding the use of human study participants and was conducted in accordance with the criteria set by the Declaration of Helsinki, as represented by the IRB-HSR approval at the University of Virginia and the other two participating institutions, and monitoring by the University of Virginia School of Medicine Clinical Trials Office and the University of Virginia Cancer Center Data Safety Monitoring Committee."

Note that full information on the approval of the study protocol must also be provided in the manuscript.

Field-specific reporting

Please select the one below that is the best fit for your research. If you are not sure, read the appropriate sections before making your selection.

- [x] Life sciences
- [ ] Behavioural & social sciences
- [ ] Ecological, evolutionary & environmental sciences

For a reference copy of the document with all sections, see nature.com/documents/nr-reporting-summary-flat.pdf

Life sciences study design

All studies must disclose on these points even when the disclosure is negative.

Sample size	The original study design calculations were based on assessing the primary immunologic endpoint, and those details were reported in our JCO paper in 2011, cited in the present manuscript. The relevant section from that 2011 paper was:<br><br>"This was an open-label, multicenter phase I/II study with random assignment to MELITAC 12.1 or MELITAC 12.6, with or without CY pretreatment (Fig 1). Primary goals were to test safety and immunogenicity. The study was designed to assess a partial ordering of immune response magnitudes among the four study arms, specifically arm D (12.6 + CY) greater than B (12.1 + CY) or C (12.6), and B or C greater than A (12.1). The study included interim safety assessments. Sample size determination was based on differences in cumulative immune response measured in the peripheral blood mononuclear cells (PBMCs) over six vaccines compared with baseline. We were interested in detecting at least a 30% increase in the four main comparisons of interest. The study was designed to test these comparisons at the two-sided 2.5% significance level (10% overall) with 90% power at the alternative, requiring 40 participants per arm (total, 160 eligible). Maximum accrual was adjusted up to 173 participants to allow for 5% ineligibility and 3% overenrollment. Patients were accrued at three participating institutions, were stratified by HLA type and institution (Table 1), and were randomly assigned within strata with various block sizes. Randomization lists were generated by the study statisticians and stored in the database, with arm assignment being released to the study coordinator only at the time of registration. The study design is presented schematically in Figure 1."<br><br>We have not replicated that text in the present paper to avoid plagiarism, but we have cited it in our text related to study design and sample size. However, we have stated in Results (2nd paragraph):<br>"For the original clinical trial protocol, the primary outcomes were safety and CD8 T cell response to 12MP, which have been reported10, while a secondary endpoint was disease-free survival (DFS). For the present ad hoc analyses of long-term followup, overall survival was the primary outcome, and DFS (here more accurately referred to as RFS) was a secondary outcome. "<br><br>Also, in the first paragraph of Methods, we have stated (slightly modified):<br>"The study was originally designed and powered to assess safety and immune responses10. Target enrollment was 40 participants per arm (total 160). At final analysis, 167 eligible participants were enrolled and treated10. For the present analysis of long-term clinical outcome, the sample size was the population of 167 eligible participants. "

Data exclusions	3 participants were found to be ineligible and are excluded from clinical outcome analysis.<br><br>These excluded patients were noted in our original 2011 report. The present analysis is of the 167 eligible patients that were noted in the original report.
Replication	Replication of this clinical trial was not feasible because of the resources involved. The trial was funded by an R01 grant from the NIH and equivalent funds would have been required to repeat it in a comparable population. Also, another factor prevented replication was reported, in our original report, that the CD8 T cell response to 12MP was lower in Arms C and D than in Arms A and B; thus, those data did not support ethics of repeating the study at that time. The finding now of enhanced overall survival for those arms in the present report represents new data to support future replication in a similar or related trial. However the data here represent about 15 years of data; so waiting to repeat the data would represent an undue delay.
Randomization	Participants were randomized 1:1:1:1 among 4 study arms, with stratification by institution and Class I HLA type.<br>This is stated in the manuscript.
Blinding	This was an open-label study. Neither participants nor the study team were blinded.

Reporting for specific materials, systems and methods

We require information from authors about some types of materials, experimental systems and methods used in many studies. Here, indicate whether each material, system or method listed is relevant to your study. If you are not sure if a list item applies to your research, read the appropriate section before selecting a response.

Materials & experimental systems

n/a	Involved in the study	
☒	☐	Antibodies
☒	☐	Eukaryotic cell lines
☒	☐	Palaeontology and archaeology
☒	☐	Animals and other organisms
☐	☒	Clinical data
☒	☐	Dual use research of concern
☒	☐	Plants

Methods

n/a	Involved in the study	
☒	☐	ChIP-seq
☒	☐	Flow cytometry
☒	☐	MRI-based neuroimaging

Clinical data

Policy information about clinical studies
All manuscripts should comply with the ICMJE guidelines for publication of clinical research and a completed CONSORT checklist must be included with all submissions.

Clinical trial registration	This trial was registered with ClinicalTrials.gov as NCT00118274
Study protocol	The full clinical trial protocol is now submitted with this manuscript as Supplemental text..
Data collection	Data were collected on case report forms & recorded in the UVA Cancer Center Clinical Trials Office Database and OnCore Database. We have added to the text of the manuscript that participants were enrolled from May 2005 to February 2008. The manuscript and the protocol explain that they were on-study for 2 years (so into 2010). Their visits on this protocol and their case-report forms were all at their treating institutions (which are specified as the University of Virginia, MD Anderson Cancer Center, and Fox Chase Cancer Center). Patients were followed then annually for survival and recurrence, which we have now specified in the Methods as "All participants were followed for survival and disease recurrence by clinicians and clinical research staff at their treating institutions."
Outcomes	Overall survival (OS) and Recurrence-free survival (RFS) are reported in this manuscript. We specify in the method: "OS was measured from study entry to last known follow-up or date of death. RFS was measured from study entry to date of disease recurrence, including new primary melanomas, or date of last known disease status."
