# Supplementary material for: Addendum: Multipeptide vaccines for melanoma in the adjuvant setting: long-term survival outcomes and post-hoc analysis of a randomized phase II trial
Source: Nat Commun. 2025 Sep 5;16:8236. doi: 10.1038/s41467-025-63690-x (PMC12413449; doi:10.1038/s41467-025-63690-x)
Supplement: Supplementary file 1 — Addendum Figures and Tables [file 41467_2025_63690_MOESM1_ESM.pdf]

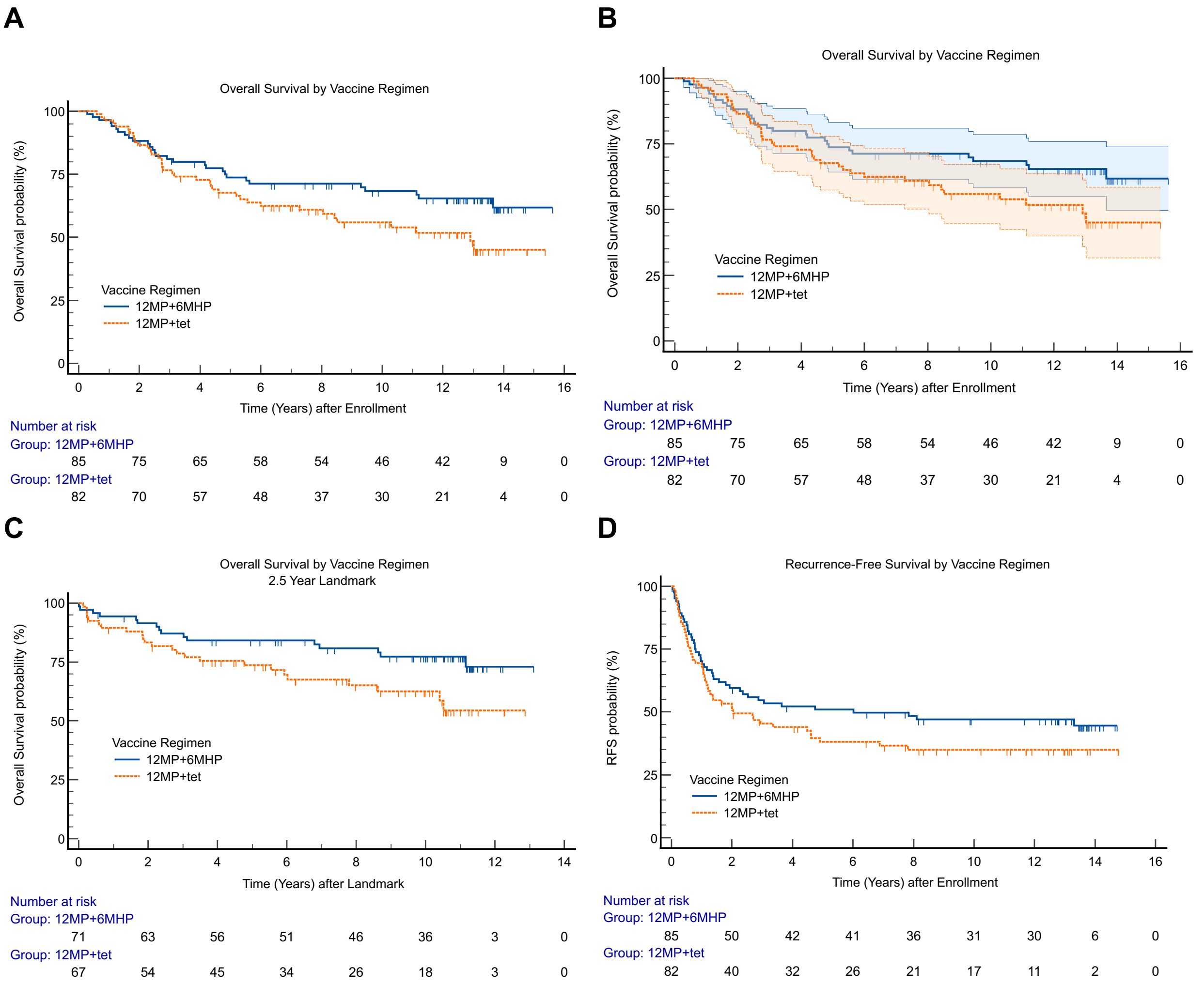

Fig. 1 | Overall survival and recurrence-free survival by vaccine regimen. A Kaplan–Meier curves for OS for all eligible participants (n=167) by vaccine regimen (HR 0.64 95% CI: 0.40-1.04, p=0.07) and **B** with 95% CI shown in shaded regions. **C** Landmark analysis for OS at 2.5 years for eligible participants (n=138) by vaccine regimen (HR 0.51, 95% CI: 0.27-0.95; p=0.04). **D** RFS for all eligible participants (n=167) by vaccine regimen (HR 0.75, 95% CI: 0.50-1.12; p=0.16). P values are from two-tailed logrank tests. Adjustments were not made for multiple comparisons. Source data are provided as a Source Data file.

A

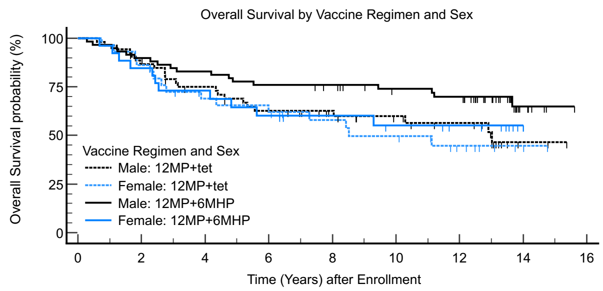

Number at risk

Group	0	2	4	6	8	10	12	14	16
Group: Male: 12MP+tet	53	45	37	30	23	18	14	2	0
Group: Female: 12MP+tet	29	25	20	18	14	12	7	2	0
Group: Male: 12MP+6MHP	59	53	48	44	42	36	34	7	0
Group: Female: 12MP+6MHP	26	22	17	14	12	10	8	2	0

B

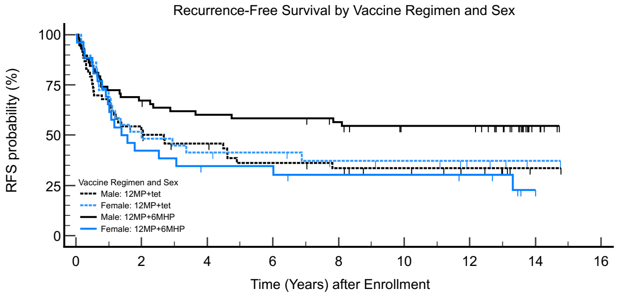

Number at risk

Group	0	2	4	6	8	10	12	14	16
Group: Male: 12MP+tet	53	25	20	15	13	10	7	1	0
Group: Female: 12MP+tet	29	15	12	11	8	7	4	1	0
Group: Male: 12MP+6MHP	59	39	34	33	30	25	25	5	0
Group: Female: 12MP+6MHP	26	11	8	8	6	6	5	1	0

C

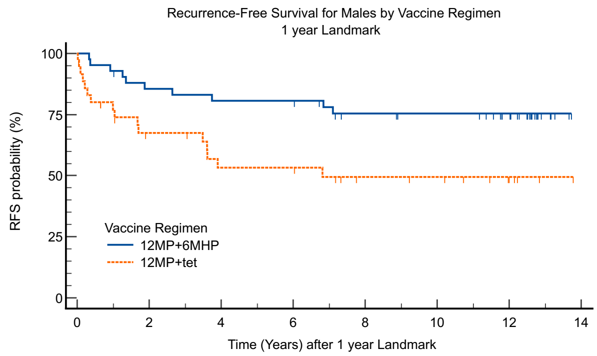

Number at risk

Group	0	2	4	6	8	10	12	14
Group: 12MP+6MHP	42	35	33	33	27	25	19	0
Group: 12MP+tet	36	20	15	15	10	9	4	0

Fig. 2. Overall survival and recurrence-free survival by vaccine regimen and sex. **A** OS for vaccine regimen and sex (p=0.17 overall): for males/6MHP vs males/tet, HR 0.58 [95% CI: 0.32–1.04]; vs female/tet, HR 0.50 [95% CI: 0.25–1.00]; vs. females/6MHP, HR 0.60 [95% CI: 0.29–1.25], **B** RFS for vaccine regimen and sex (p=0.08 overall): for males/6MHP vs males/tet, HR 0.57 [95% CI: 0.35–0.94]; vs female/tet, HR 0.63 [95% CI 0.35–1.12]; vs. females/6MHP, HR 0.50 [95% CI: 0.27–0.93], **C** Landmark analysis for RFS at 1 year for males (n=78) by vaccine regimen (HR 0.37, 95% CI: 0.17-0.81; p=0.01). P values are from two-tailed logrank tests. Adjustments were not made for multiple comparisons. Source data are provided as a Source Data file.

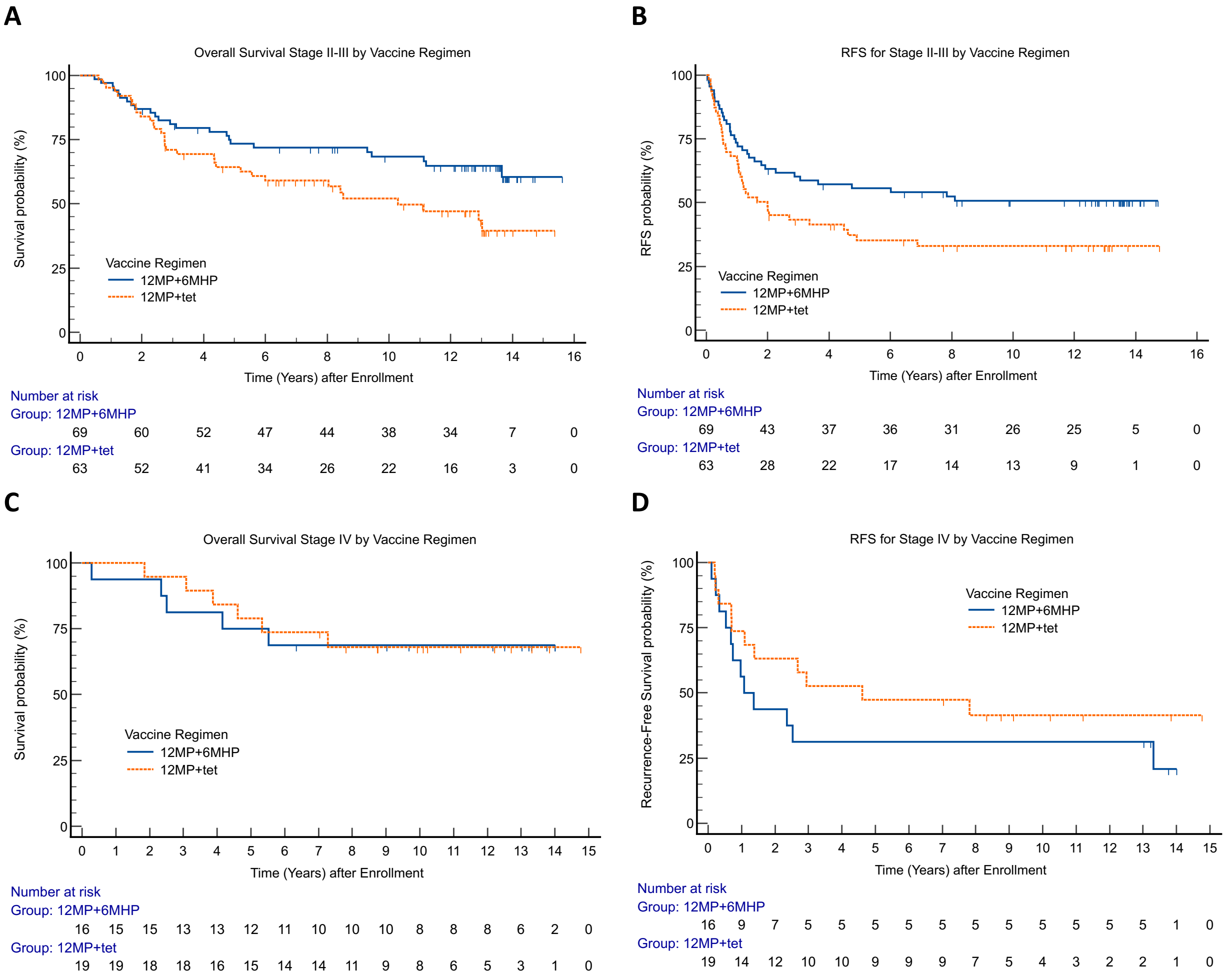

Fig. 3. Overall survival and recurrence-free survival by vaccine regimen and AJCC v8 stage. **A** OS by vaccine regimen for stage II-III participants (HR 0.56, 95% CI: 0.33-0.96; p=0.03), **B** RFS by vaccine regimen for stage II-III participants (HR 0.61, 95% CI: 0.38-0.98; p=0.04). **C** OS by vaccine regimen for stage IV participants (HR 1.05, 95% CI: 0.32-3.46; p=0.93), **D** RFS by vaccine regimen for stage IV participants (HR 1.60, 95% CI: 0.69-3.71; p=0.27). P values are from two-tailed logrank tests. Adjustments were not made for multiple comparisons. Source data are provided as a Source Data file.

Table 1. Cox regression model for overall survival for intention-to-treat population (n = 167)

Initial model (p < 0.0001, Chi-squared 37.3), OS all eligible patients (n = 167) including study arm and 6 clinical covariates				
**Covariate**	***Detail***	***p value***	***HR***	***95% CI***
Age	≤ 40 vs > 40 years	**0.002***	0.045	0.006 to 0.331
Sex	Female vs male	**0.024***	1.834	1.083 to 3.103
Study Arm	A (12MP+tet) vs D (12MP+6MHP+Cy)	**0.016***	2.436	1.185 to 5.009
	B (12MP+Tet+Cy) vs. D (12MP+6MHP+Cy)	0.308	1.466	0.703 to 3.055
	C (12MP+6MHP) vs. D (12MP+6MHP+Cy)	0.337	1.436	0.686 to 3.005
Advanced disease status	Recurrence vs initial diagnosis	0.228	0.727	0.433 to 1.220
LDH level	High vs normal or not done	0.221	2.146	0.632 to 7.288
AJCC stage, v8	Stage IV vs II-III	0.211	0.640	0.318 to 1.287
ECOG PS score	1 vs 0	0.418	1.347	0.655 to 2.767
Refined model (p < 0.0001, Chi-squared 33.8) OS all eligible patients (n = 167) Beginning with study arm, vaccine regimen and Cy arms, with stepwise refinement				
**Covariate**	***Detail***	***p value***	***HR***	***95% CI***
Age	≤ 40 vs > 40 years	**0.003***	0.052	0.007 to 0.380
Sex	Female vs male	**0.034***	1.707	1.029 to 2.834
Study Arm	A (12MP+tet) vs D (12MP+6MHP+Cy)	**0.019***	2.239	1.100 to 4.559
	B (12MP+Tet+Cy) vs. D (12MP+6MHP+Cy)	0.314	1.427	0.700 to 2.923
	C (12MP+6MHP) vs. D (12MP+6MHP+Cy)	0.345	1.346	0.647 to 2.801
AJCC stage, v8	Stage IV vs II-III	*0.052*$^{\#}$	0.525	0.274 to 1.004

Abbreviations: HR, hazard ratio; CI, confidence interval; LDH, lactate dehydrogenase; AJCC, American Joint Committee on Cancer (8th edition); ECOG PS, Eastern Cooperative Oncology Group Performance Status; 12MP, 12 class I MHC-restricted melanoma peptides; tet, tetanus toxoid helper peptide; 6MHP, six melanoma-specific helper peptides; Cy, cyclophosphamide

*Significant, $p < 0.05$ (bolded); $^{\#}$ $0.1 > p > 0.05$ (italics); Adjustments were not made for multiple comparisons.

Table 2. Cox regression models for overall survival for 2.5-year landmark (n = 138)

Refined model (p = 0.0003, Chi-squared = 25.5): OS for 2.5y landmark (n = 138) with study Arm				
**Covariate**	***Detail***	***p value***	***HR***	***95% CI***
Study Arm	A (12MP+Tet) vs. D (12MP+6MHP+Cy)	**0.011***	3.797	1.357 to 10.63
	B (12MP+Tet+Cy) vs. D (12MP+6MHP+Cy)	**0.022***	3.209	1.183 to 8.705
	C (12MP+6MHP) vs. D (12MP+6MHP+Cy)	0.132	2.218	0.786 to 6.256
Age	≤ 40 vs > 40 years	**0.021***	0.094	0.013 to 0.698
ECOG PS score	1 vs 0	**0.036***	2.562	1.066 to 6.157
Advanced disease	Recurrence vs initial diagnosis	**0.047***	0.520	0.273 to 0.992
Refined model (p = 0.0001, Chi-squared = 22.97): OS for 2.5y landmark (n = 138) with vaccine regimen and Cy arms				
**Covariate**	***Detail***	***p value***	***HR***	***95% CI***
Vaccine Regimen†	12MP+tet vs. 12MP+6MHP	**0.015***	2.238	1.168 to 4.289
Age	≤ 40 vs > 40 years	**0.025***	0.102	0.014 to 0.747
ECOG PS score	1 vs 0	**0.042***	2.351	1.031 to 5.360
Advanced disease	Recurrence vs initial diagnosis	*0.060*#	0.542	0.286 to 1.026

Abbreviations: HR, hazard ratio; CI, confidence interval; ECOG PS, Eastern Cooperative Oncology Group Performance Status; 12MP, 12 class I MHC-restricted melanoma peptides; tet, tetanus toxoid helper peptide; 6MHP, six melanoma-specific helper peptides; Cy, cyclophosphamide

* Significant, $p < 0.05$ (bolded); # $0.1 > p > 0.05$ (italics); Adjustments were not made for multiple comparisons.

† Vaccine regimen 12MP + tet includes study arms A+B; vaccine regimen 12MP + 6MHP includes study arms C+D

References Cited

1. Ninmer EK, Zhu H, Chianese-Bullock KA, von Mehren M, Haas NB, Ross MI, Dengel LT, Slingluff CL, Jr. Multipeptide vaccines for melanoma in the adjuvant setting: long-term survival outcomes and post-hoc analysis of a randomized phase II trial. Nature communications. 2024;15(1):2570. Epub 20240322. doi: 10.1038/s41467-024-46877-6. PubMed PMID: 38519525; PMCID: PMC10959948.
